# Characteristics of maternity waiting homes and the women who use them: Findings from a baseline cross-sectional household survey among SMGL-supported districts in Zambia

**DOI:** 10.1371/journal.pone.0209815

**Published:** 2018-12-31

**Authors:** Jody R. Lori, Carol J. Boyd, Michelle L. Munro-Kramer, Philip T. Veliz, Elizabeth G. Henry, Jeanette Kaiser, Gertrude Munsonda, Nancy Scott

**Affiliations:** 1 School of Nursing, University of Michigan, Ann Arbor, MI, United States of America; 2 School of Public Health, Boston University, Boston, MA, United States of America; 3 Africare-Zambia, Lusaka, Zambia; Johns Hopkins School of Public Health, UNITED STATES

## Abstract

**Objective:**

Maternity waiting homes (MWHs) have been identified as one solution to decrease maternal morbidity and mortality by bringing women living in hard-to-reach areas closer to a hospital or health center that provides emergency obstetric care. The objective of this study was to obtain data on current MWH characteristics and the women who use them as well as women’s perceptions and experiences with MWHs among seven Saving Mothers Giving Life (SMGL) supported districts in Zambia.

**Methods:**

A cross-sectional household survey design was used to collect data from 2381 mothers who delivered a child in the past 13 months from catchment areas associated with 40 health care facilities in seven districts. Multi-stage random sampling procedures were employed with probability proportionate to population size randomly selected. Logistic regression models, Chi-square, and independent t-tests were used to analyze the data.

**Results:**

Women who lived 15–24 km from a health care facility were more likely to use a MWH when compared to women who lived 9.5–9.9 km from the nearest facility (AOR: 1.722, 95% CI: 1.450, 2.045) as were women who lived 25 km or more (AOR: 2.098, 95% CI: 1.176, 3.722.881). Women who were not married had lower odds of utilizing a MWH when compared to married women (AOR: 0.590, 95% CI: 0.369, 0.941). Over half of mothers using a MWH prior to delivery reported problems at the MWH related to boredom (42.4%), management oversight (33.3%), safety (33.4%), and quality (43.7%). While the study employs a robust design, it is limited by its focus in Saving Mothers Giving Life districts.

**Conclusion:**

MWHs, which currently take many forms in Zambia, are being used by over a third of women delivering at a health facility in our study. Although over half of women using the existing MWHs noted crowdedness and nearly a third reported problems with the physical quality of the building as well as with their interaction with staff, these MWHs appear to be bridging the distance barrier for women who live greater than 9.5 km from a health care facility.

## Introduction

Providing shelter near an obstetric facility for women living in remote geographic areas prior to delivery is not a new concept [1]. Descriptions of Maternity Waiting Homes (MWHs) date back to the 1950’s [2,3] with examples of their use on multiple continents [4–6]. In a recent Maternal Health Lancet Series, Campbell et al. [7] identified MWHs as one solution to decrease maternal morbidity and mortality by bringing women living in hard-to-reach areas closer to a hospital or health center that provides Emergency Obstetric Care (EmOC).

Maternity Waiting Homes, sometimes called Mothers’ Shelters or Maternity Waiting Shelters, are facilities that house women in the last few weeks of pregnancy with easy access to a health care facility capable of providing EmOC [8]. Once labor begins, women can easily transfer to the health care facility for delivery, thereby providing access to a skilled provider for intrapartum care. Multiple models of MWHs are described in the literature from risk-based models (i.e. women with risk factors are encouraged to stay in a MWH to decrease poor outcomes) [9] to models that follow a national strategy (i.e., any pregnant woman is allowed to stay at a MWH prior to delivery) [10]. The concept of risk-based models influenced some of the early development of MWHs based on the premise that selecting women with risk factors and advising them on the use of a MWH prior to delivery could help prevent poor outcomes, consequently playing an important role [8]. Models that do not use a risk approach often are intended to improve access for women living the farthest distance from the health facility, allowing women to reach the facility in time for delivery [8,10].

In 2015, as part of a coordinated evaluation effort, the Maternity Homes Alliance for Zambia was formed between the government of Zambia, donors, implementing partners, university evaluators, and the Saving Mothers Giving Lives (SMGL) project, to provide robust data for decision-makers on the effectiveness of MWHs in Zambia as an intervention for improving maternal and newborn health outcomes. All partners supported the concept of MWHs as a health system strengthening effort. During a 2014 formative evaluation of existing MWHs in rural Zambia, we found MWHs varied from a space within the clinic for women to sleep at night while sitting outside during clinic hours, to make-shift structures with poor amenities and few beds [11,12,13].

Based on our formative data findings and to provide generalizable data, the Maternity Homes Alliance reached a consensus on a minimum core model for MWHs in rural Zambia with criteria within three domains: (i) infrastructure, equipment, & supplies, (ii) policies, management and finances, and (iii) linkages and services. At the time of the survey, 20 MWHs were being constructed or refurbished to meet the minimum core model near a rural health facility capable of providing Basic Emergency Obstetric and Newborn Care (BEmONC). An additional four MWHs were under construction near a district level hospital with Comprehensive Emergency Obstetric and Newborn Care (CEmONC) capacity. These newly constructed or refurbished MWHs will allow the Maternity Homes Alliance to evaluate whether the minimum core MWH model increases access to high quality intrapartum care among mothers living farthest (defined as greater than 9.5 km from a health care facility). Development of the minimum core model responded to community input from our formative research to improve the physical structure of MWHs, strengthen local governance and ownership, and maximize the sustainability of the model over time [11,12].

The purpose of this study is to report on baseline data obtained prior to the opening of the minimum core model MWHs in three provinces in Zambia. We asked participants (i.e., mothers who gave birth during the previous 13 months) about where they delivered, whether they used a MWH, and their perceptions (i.e. quality and safety) as well as their experiences with existing MWHs in their districts. We also gathered data on the characteristics of the existing MWHs used and the women who chose to use them.

## Methods

A cross-sectional household survey was used to collect data from the 40 study cluster catchment areas involved in the study (20 health care facilities identified to receive a minimum core model MWH and 20 comparison facilities). A team of local residents, hired as research assistants, literate in the appropriate local languages and English, and with previous experience collecting quantitative data for research studies, were trained in human subjects’ protection and qualitative and quantitative data collection methods during a 5-day training. Data were captured electronically using SurveyCTO Collect Software installed on encrypted tablets.

The survey was pre-tested among 50 respondents (women who had delivered a baby within the past year) recruited from a local clinic. All four languages were represented (Bembe, Nyanja Tonga, and Tumbuka). Adjustments were made in response to the pre-test, mainly changing more formal translations into the vernacular. No major changes were required. When possible, questions on the survey were drawn or adapted from existing instruments [14].

### Study setting

Zambia is a land locked country in sub-Saharan Africa. At the time of the survey, there were 10 provinces and 74 districts. According to WHO, the current population is 16 million with a maternal mortality ratio of 224 per 100,000 live births [14]. While the country has had marked improvements in maternal health, a woman's lifetime risk of maternal death remains high at 1 in 79. It is for these reasons that Zambia was chosen as one of two original countries in sub-Saharan Africa to take part in a five-year public-private partnership aimed at accelerating reduction in maternal and newborn mortality [15]. Launched in 2012, SMGL takes a health systems approach to improve access to clean, safe childbirth services and timely emergency care for pregnant women [16].

Seven SMGL districts (Choma, Kalomo, Lundazi, Mansa, Nyimba, Pemba, and Chembe) in three provinces (Eastern, Luapula, and Southern) are targeted for this study. Although they are now part of SMGL districts, at the time of data collection, SMGL activities had not commenced in Choma or Pemba. To ensure facilities included in the study are resourced appropriately to adequately manage obstetric complications, 40 rural health facilities, located within 2 hours of travel time to a CEmONC referral facility were selected from a list of eligible facilities that met the following inclusion criteria: (i) capable of performing a minimum of 5 of the 7 BEmONC signal functions and (ii) providing intrapartum care to a minimum of 150 women per year; or (i) staffed with at least one skilled birth attendant on staff, (ii) routinely providing active management of third stage of labor, and (iii) having no stock outs of oxytocin or magnesium sulfate in the last 12 months. We chose 12 months to ensure facilities had stability in commodities and human resources. In 4 districts health facilities were randomly chosen, while in the remaining 3 districts health facilities meeting criteria were purposively sampled from eligible facilities with input from district health teams. Selection and assignment of study clusters is described in detail elsewhere [17].

### Study sample

The survey was conducted in the 40 study cluster catchment areas in the seven SMGL districts over three weeks in March 2016. These data will later become part of a robust evaluation study of 20 sites receiving the minimum core model MWH and 20 comparison sites. For the survey, multi-stage random sampling procedures were employed in the seven districts with probability proportionate to population size randomly selected. The sample frame of clusters included villages located more than 9.5 km from the health care facility within their catchment area along the most direct route, identified through geo-coding. Details of the sampling frame and protocol for this study are reported elsewhere [17]. In the second stage of sampling, all households within the selected villages were listed and then randomly selected.

The sample consisted of women who met the following inclusion criteria: (i) had delivered in the last 13 months (to obtain recent delivery data and reduce recall bias), (ii) 15 years of age or older, and (iii) lived in a village that was 9.5 km or farther from one of the health care facilities included in our sample. To ensure a representative sample of the target population, a multi-stage random sampling procedure was used: (i) villages 9.5 km or greater from the health care facility along the most direct route were identified within each of the seven districts, (ii) households within each village were randomly ordered and approached to contact an eligible respondent (i.e., mother who has recently delivered), and (iii) if more than one eligible respondent was in the household, one of these respondents was randomly sampled.

### Data collection

Participants from eligible households were recruited, consented, and enrolled in the study. The research assistant recorded the geo-location of the village center to determine distance to the nearest health care facility. Eligible participants provided written informed consent, which was documented in writing or with a fingerprint and witness signature prior to beginning the survey. If participants were under the age of 18 years, child assent and guardian or husband (if over the age of 18 years) was obtained. Each household survey took approximately 45 minutes. The final sample included 2381 mothers who had delivered a child in the last 13 months for a response rate of 86.9%. Of the women who were eligible but did not respond, 280 mothers were unavailable, 60 refused participation, and 20 mothers withdrew after beginning the survey or had incomplete surveys and were dropped from the study. Participants received a small token of appreciation (chitenge, a local fabric) in acknowledgment of their time. Ethical approval was obtained from Boston University Institutional Review Board (IRB), University of Michigan IRB, and the ERES Converge Research Ethics Committee in Zambia.

### Data analysis

The analytic strategy for the current study was to: (i) provide descriptive statistics for the study sample, (ii) examine key characteristics of mothers and households who used a MWH for their most recent delivery, (iii) examine the prevalence of MWH utilization for women delivering in the year prior to the start of the study, and (iv) examine perceived characteristics of MWHs among women who delivered at these locations across the seven districts included in this study.

For the analyses, STATA 14.0 was used to estimate the models outlined above [18]. All logistic regression models provide adjusted odds ratios (AOR) and 95% confidence intervals (95% CI) and accounted for the multi-stage sampling procedure. Moreover, Chi-square and independent means t-tests were used to assess differences between individual districts (when compared to the combined group of respondents in remaining districts to maintain a large enough sample to make meaningful comparisons). Missing data were handled using listwise deletion.

## Results

### Demographics

The demographic characteristics of the mothers who participated in the survey are shown in Table 1. The majority of mothers were between the ages of 20 and 29 years of age, had some primary education, were protestant, married, had been pregnant two or more times, had two or more live births, and indicated delivering in some type of health care facility with respect to their most recent birth within the past 13 months, with 31.5% utilizing a MWH for any reason before or after their most recent delivery.

**Table 1:** Descriptive statistics for mothers who participated in the Maternity Homes Alliance survey in Zambia (n = 2369).

**Mother's Characteristics**	No MWH Use n = 1622 (68.5%)	Used MWH n = 747 (31.5%)			**Household Characteristics**	No MWH Use n = 1622 (68.5%)	Used MWH n = 747 (31.5%)	
**Woman's Age**	%/Mean (SD)	%/Mean (SD)	Sig.^a^		**Distance to facility (km)**	%/Mean (SD)	%/Mean (SD)	Sig.^a^
*Mean age*	26.3 (7.02)	25.7 (6.84)	*		*Mean distance*	14.7 (8.18)	17.0 (11.3)	***
15 to 19	16.7% (n = 271)	20.8% (n = 154)			9.5–9.9 km	13.5% (n = 219)	10.7% (n = 80)	***
20 to 24	31.8% (n = 515)	32.5% (n = 241)			10 to 11.9 km	31.5% (n = 509)	25.2% (n = 188)	***
25 to 29	19.6% (n = 317)	18.2% (n = 135)			12 to 14.9 km	27.8% (n = 449)	23.6% (n = 176)	***
30 to 34	16.4% (n = 266)	15.4% (n = 114)			15 to 24.9 km	20.0% (n = 324)	22.7% (n = 206)	***
35 and older	15.4% (n = 249)	13.2% (n = 98)			25 or more km	7.2% (n = 117)	12.8% (n = 95)	***
**Highest Level of Education**					**Household headship**			
No Education	16.2% (n = 263)	13.2% (n = 98)			Male	75.4% (n = 1223)	79.1% (n = 591)	*
Some Primary	40.1% (n = 650)	42.2% (n = 313)			Female	9.7% (n = 158)	9.9% (n = 74)	*
Completed Primary	19.2% (n = 312)	21.6% (n = 160)			Unknown for study	14.9% (n = 241)	11.0% (n = 82)	*
Some Secondary	22.9% (n = 372)	21.4% (n = 159)			**Household Size**			
Completed Secondary	1.5% (n = 24)	1.6% (n = 12)			*Mean household size*	7.00 (3.54)	6.95 (3.66)	
**Religion**					1 to 3 people	10.9% (n = 777)	13.1% (n = 777)	
Catholic	12.5% (n = 202)	11.3% (n = 84)			4 to 6 people	40.2% (n = 777)	38.2% (n = 777)	
Protestant	71.0% (n = 1152)	72.4% (n = 537)			7 or more people	49.0% (n = 777)	48.7% (n = 777)	
Other	16.5% (n = 268)	16.3% (n = 121)			**Number of children**			
**Marital Status**					*Mean number of children*	3.98 (2.37)	3.95 (2.49)	
Married	86.7% (n = 1407)	90.8% (n = 675)	**		1 to 3 children	46.0% (n = 742)	47.9% (n = 355)	
Not married	13.3% (n = 215)	9.2% (n = 68)	**		4 to 6 children	42.6% (n = 687)	39.9% (n = 296)	
**Number of times Pregnant**					7 or more children	11.4% (n = 184)	12.1% (n = 90)	
*Mean number of pregnancies*	3.92 (2.58)	3.74 (2.45)			**Housing Characteristics**			
1 time	21.1% (n = 343)	22.0% (n = 164)			Non-improved water source	57.0% (n = 924)	54.3% (n = 405)	
2 to 3 times	30.2% (n = 490)	30.6% (n = 228)			Time to obtain water 30+ min.	17.9% (n = 235)	18.6% (n = 113)	
4 or more times	48.6% (n = 789)	47.5% (n = 354)			Non-improved toilet	90.9% (n = 1474)	88.0% (n = 657)	*
**Number of live births**					No electricity	99.6% (n = 1613)	99.7% (n = 744)	
*Mean number of births*	3.64 (2.37)	3.47 (2.30)			House flooring made of earth	87.6% (n = 1420)	88.9% (n = 664)	*
1 birth	22.6% (n = 366)	24.7% (n = 184)			Charcoal or wood cooking fuel	99.4% (n = 1613)	99.7% (n = 745)	
2 to 3 births	31.8% (n = 514)	31.8% (n = 237)			**Means of transportation**			
4 or more births	45.6% (n = 737)	43.5% (n = 324)			Bicycle	65.2% (n = 1012)	65.2% (n = 471)	
**Delivered in a health facility**	74.3% (n = 1204)	96.8% (n = 723)			Animal drawn cart	12.2% (n = 189)	15.7% (n = 113)	*
*Hospital (health facility)*	*9*.*5%* (n = 154)	*14*.*2%* (n = 106)	***		Car-truck	2.1% (n = 32)	2.2% (n = 16)	
*Rural Clinic (health facility****)***	*64*.*7%* (n = 1050)	*82*.*6%* (n = 617)	***		Motorbike	2.8% (n = 44)	3.3% (n = 24)	
*Home/Other Home*	*21*.*0%* (n = 341)	*2*.*9%* (n = 22)	***					
*On road/in transport*	*4*.*7%* (n = 76)	*0*.*3%* (n = 2)	***					

*p < .05.

**p < .01.

***p < .001.

% = Percent; (SD) = Standard Deviation; MWH = Maternity Waiting Home; n = frequency. Sample sizes may vary due to missing data across items.

^a^All analyses use χ2 (chi-square) or independent means t-tests (Fisher's Exact Test was used for analyses with small cell counts).

Use of the MWH was aggregated into three categories: 1) antenatal stays, 2) awaiting delivery, and 3) postpartum stays. The average number of nights stayed for any reason antenally was 5.8 nights (SD = 13.1), the median number of nights stayed was 2 (51 women indicating an antenatal stay). The average number of nights stayed awaiting delivery was 11.3 nights (SD = 12.7), the median number of nights stayed was 7 (698 women indicated staying while awaiting delivery). Finally, the average number of nights stayed for a postpartum was 2.0 nights (SD = 3.1), the median number of nights stayed was 1 (98 women indicated staying at the MWH postpartum).

Additionally, women who reported delivering in a hospital or rural health clinic were significantly more likely to use a MWH and mothers who reported delivering at home or on the way to the clinic were significantly less likely to report using a MWH.

The household characteristics indicate the mean distance to the nearest health care facility from the village center was roughly 15 km for the mothers who did not use a MWH prior to delivery and 17 km for those who did use a MWH. The households were predominantly headed by males and included approximately seven people per household, four of which were typically 14 years of age or younger. The vast majority of participants reported living in earthen houses with minimal accesses to electricity and plumbing. While most households had access to bicycles, very few had access to automobiles.

### Characteristics Associated with facility delivery and MWH use

Table 2 shows that household distance to the nearest health care facility had little impact on where mothers delivered. However, mothers who lived 15 km or greater were more likely to use a MWH when compared to women who lived within 9.5–9.9 km of the nearest health care facility.

**Table 2:** Logistic regression examining key mother and household characteristics to predict MWH use and delivery site.

	Delivered at any Health Facility (81.4%) (n = 2,253)				Delivered at Hospital (11.0%) (n = 2,253)				Delivered at Rural Health Clinic (70.4%) (n = 2,253)				Used a MWH (31.5%) (n = 2,245)			
	AOR	95% CI			AOR	95% CI			AOR	95% CI			**AOR**	**95% CI**		
**Distance to health care facility in catchment area (km)**																
9.5–9.9 km (reference)																
10 to 11.9 km	0.698	0.424	,	1.140	0.711*	0.548	,	0.924	0.927	0.686	,	1.252	0.973	0.702	,	1.347
12 to 14.9 km	0.627	0.380	,	1.036	0.642	0.385	,	1.069	0.889	0.549	,	1.438	1.015	0.781	,	1.319
15 to 24.9 km	0.780	0.496	,	1.227	1.017	0.724	,	1.428	0.854	0.567	,	1.287	1.722***	1.450	,	2.045
25 or more km	1.231	0.689	,	2.199	1.303	0.685	,	2.477	0.941	0.503	,	1.760	2.098*	1.176	,	3.744
**Woman's Age (continuous)**	1.009	0.995	,	1.024	1.017	0.968	,	1.068	1.001	0.973	,	1.029	0.992	0.975	,	1.009
**Highest Level of Education**																
No education																
Some Primary	1.248	0.822	,	1.895	1.009	0.707	,	1.440	1.187	0.920	,	1.531	1.198	0.886	,	1.621
Completed Primary or higher	1.460	0.969	,	2.199	0.975	0.611	,	1.555	1.337**	1.107	,	1.614	1.134	0.756	,	1.701
**Marital Status**																
Married																
Not married	0.752	0.537	,	1.054	1.004	0.339	,	2.972	0.811	0.472	,	1.391	0.590*	0.369	,	0.941
**Number of births (continuous)**	0.845***	0.772	,	0.925	0.915	0.825	,	1.016	0.914	0.820	,	1.019	0.963	0.875	,	1.059
**Means of transportation**																
Bicycle	1.214	0.969	,	1.521	0.974	0.779	,	1.218	1.166	0.952	,	1.429	0.983	0.673	,	1.437
Animal drawn cart	0.945	0.599	,	1.493	1.122	0.815	,	1.543	0.911	0.717	,	1.158	1.280	0.731	,	2.241
Car/truck	1.739*	1.050	,	2.881	1.518	0.570	,	4.043	1.090	0.565	,	2.103	0.991	0.581	,	1.689
Motorbike	1.998	0.925	,	4.316	1.696*	1.036	,	2.775	1.107	0.692	,	1.771	0.987	0.603	,	1.615

*p < .05.

**p < .01.

***p < .001.

All binary logistic regression analyses estimating the adjusted odds ratios (AOR) presented above included the following predictor variables within each model (four models in total): distance to health care facility, woman’s age, highest level of education, marital status, number of births, and means of transportation. Additionally, all analyses account for sampling design based on the seven districts selected to participate. Sample sizes vary due to missing data across the variables used in the models.

In addition, Table 2 shows key characteristics of mothers associated with health facility and MWH use in the sample. Mothers who completed primary schooling or more had slightly higher odds when compared to mothers who had no education (AOR: 1.337, 95% CI: 1.107, 1.641) of indicating they delivered at a rural clinic. Mothers who were not married had lower odds of utilizing a MWH when compared to married women (AOR: 0.590, 95% CI: 0.369, 0.941). Finally, mothers who had access to either a car/truck or motorbike had higher odds of delivering at any type of health facility (AOR: 1.739, 95% CI: 1.050, 2.881) and delivering at a hospital (AOR: 1.696, 95% CI: 1.036–2.775), respectively.

### Perceived characteristics of MWHs

Table 3 provides several of the problems noted and perceived characteristics of MWHs among women who used these facilities during their most recent delivery across the seven districts involved in the Maternity Homes Alliance initiative in Zambia. It also reflects resources women contributed and their overall impression of the MWH. It should also be highlighted that approximately one-third of mothers stayed at an existing MWH. Lundazi district had the highest percentage of mothers using a MWH prior to delivery (43.6%), while Mansa district had the lowest percentage (10.7%). Finally, post hoc logistic regression analyses within each site found no unique associations with MWH utilization with respect to key characteristics of mothers and households (i.e., same variables used in the logistic regression analyses presented in Table 2, data not shown).

**Table 3:** Perceived characteristics of maternity waiting homes (only women who indicated using a MWH) by district of residence.

	**All Districts**	**Choma**	**Kalomo**	**Lundazi**	**Mansa**	**Nyimba**	**Pemba**	**Chembe**
	(n = 747)	(n = 82)	(n = 244)	(n = 264)	(n = 54)	(n = 65)	(n = 27)	(n = 11)
**% using MWH in location**	31.5%	25.30%	43.00%	43.60%	10.80%	30.00%	35.50%	14.10%
	%/mean (SE)	%/mean (SE)	%/mean (SE)	%/mean (SE)	%/mean (SE)	%/mean (SE)	%/mean (SE)	%/mean (SE)
**% indicating Problems with MWH**								
Access to cooking area	30.10%	28.00%	46.10%***	22.70%***	25.00%	15.40%**	15.40%	30.00%
Boredom	42.40%	49.90%	47.20%	37.50%	30.80%	44.60%	44.00%	60.00%
Cleanliness	27.00%	24.10%	40.30%***	22.20%*	9.60%**	10.90%**	30.80%	30.00%
Crowdedness	53.50%	51.90%	67.70%***	48.10%*	23.10%***	50.80%	69.20%	40.00%
Cultural Appropriateness	15.20%	20.50%	20.60%**	10.30%**	15.40%	6.20%*	23.10%	11.10%
Management Oversight	33.30%	38.60%	45.70%***	26.50%**	21.20%	15.90%**	30.80%	75.00%*
Quality	43.70%	41.50%	60.60%***	39.60%	17.30%***	28.60%*	34.60%	30.00%
Safety	33.40%	33.70%	48.90%***	23.80%***	19.20%*	28.10%	30.80%	20.00%
Presence of Staff	30.70%	34.90%	44.60%***	19.50%***	19.20%	29.20%	26.90%	50.00%
Friendliness of Staff	26.60%	24.40%	39.20%***	18.80%***	11.80%*	23.40%	24.00%	50.00%
**Characteristics of MWH**								
Bed available	56.50%	56.60%	34.30%***	57.80%	98.10%***	76.90%***	96.20%***	100.00%**
Share bed	23.50%	18.10%	11.20%***	24.40%	60.40%***	41.50%***	19.20%	40.00%
Sleep Under Mosquito Net	44.10%	28.90%**	23.60%***	52.30%***	83.00%***	56.90%*	84.60%***	55.60%
Oriented to Rules (24 hours)	63.60%	55.60%	40.80%***	81.70%***	86.80%***	64.60%	61.50%	60.00%
Access to Water	93.00%	94.00%	90.10%*	95.80%*	86.80%	93.80%	100.00%	100.00%
Access to Light	69.60%	63.90%	51.10%***	79.80%***	94.30%***	70.80%	96.20%**	88.90%
Bathing Area	78.40%	86.60%	60.90%***	87.80%***	88.70%	78.50%	84.60%	100.00%
Safe Space for Belongings	60.90%	58.50%	42.10%***	73.80%***	84.90%***	58.50%	69.20%	90.00%
Attended Health Ed. Sessions	48.40%	39.00%	30.30%***	65.00%***	73.60%***	33.80%*	53.80%	80.00%*
Cooking Space	82.00%	81.90%	72.70%***	87.00%*	94.20%*	90.80%	92.30%	80.00%
Cooks Space Covered	83.10%	71.60%**	70.80%***	90.30%***	89.80%	98.30%***	87.00%	87.50%
Acquired Skills	14.00%	7.20%	6.10%***	15.50%	53.80%***	12.30%	23.10%	30.00%
Asked to Contribute Money	14.50%	6.00%*	8.60%***	26.50%***	11.30%	7.70%	3.80%	9.10%
**Contributed Resources to MWH**								
Contributed Labor to MWH	1.10%	0.00%	0.40%	2.30%*	1.90%	0.00%	0.00%	0.00%
Contributed Livestock or Poultry	0.70%	0.00%	0.40%	1.10%	1.90%	0.00%	0.00%	0.00%
Contributed Food to MWH	4.00%	0.00%	1.70%*	6.40%*	5.60%	7.70%	0.00%	0.00%
**Overall Impression of MWH**								
Recommend MWH	92.40%	91.50%	87.90%**	94.60%	96.20%	96.90%	96.20%	90.00%
Intend to use MWH in the future	88.90%	91.00%	83.05%***	90.40%	98.00%*	93.80%	96.20%	90.00%
Not Satisfied with MWH	20.10%	19.50%**	35.20%***	12.90%***	9.40%***	9.20%*	7.70%	0.00%
**Average Distance** **to Health Facility**	15.44 (.192)	13.43*** (.146)	16.81*** (.309)	18.34*** (.636)	12.66*** (.139)	13.91* (.364)	15.48 (.715)	13.76 (.354)

*p < .05.

**p < .01.

***p < .001.

MWH = maternity waiting home; % = percent, (SE) = Standard Error

All analyses use χ2 or independent means t-tests (Fisher's Exact Test was used for analyses with small cell counts). All analyses compare individual districts with the remaining districts combined. For instance, women who used mother shelters in Choma were compared to all other women who used mother shelters in the other six districts.

With respect to problems associated with MWHs, over half of mothers indicated some dissatisfaction with crowdedness at the MWH. Over one-third identified problems at the MWH related to boredom, management oversight, safety, and quality. The overwhelming majority of mothers had access to water, light, a bathing area, and appropriate cooking spaces at the maternity waiting home. However, certain aspects such as having access to a bed or mosquito nets to sleep under tended to be reported by only half of the mothers during their stay. Aspects related to beds varied significantly from district to district. Additionally, while less than a quarter of mothers indicated receiving skills during their stay at the MWH, roughly half attended some type of health education session, with some districts being more or less responsive to providing these educational resources.

Very few women reported contributing resources to the MWH such as food, labor, or livestock and poultry. Finally, mothers’ overall impressions of the MWHs were quite positive, with 79.9% of mothers being satisfied with their stay at the MWH, 92.4% indicating they would recommend the MWH to other women, and 88.9% indicating they will use a MWH again for their next delivery.

### Community mobilization and birth preparedness

The majority of women in our survey heard about MWHs from a health care worker (74.4%) or a community member (38.1%) who was a volunteer in a Safe Motherhood Action Group. These groups were established in 2003 as part of the national safe motherhood program to improve the health of communities through community-based interventions and scaled up under SMGL [19].

Overall, 82.6% of mothers had money set aside for delivery with 61.3% believing they had enough money set aside. Of those who used a MWH, 85.3% had set money aside. Among mothers that did not use a MWH, 81.4% had set money aside for delivery. Of the sample that did not use a MWH (n = 1622), 34.3% stated it was because there was no MWH available in their area, with the highest percentage from Mansa (52.9%), a district that also recorded the lowest MWH use (10.8%). See Table 4.

**Table 4:** Community mobilization and birth preparedness by district.

	All Districts	Choma	Kalomo	Lundazi	Mansa	Nyimba	Pemba	Chembe
	(n = 2381)	(n = 327)	(n = 572)	(n = 606)	(n = 503)	(n = 217)	(n = 76)	(n = 80)
	%/mean (SE)	%/mean (SE)	%/mean (SE)	%/mean (SE)	%/mean (SE)	%/mean (SE)	%/mean (SE)	%/mean (SE)
**Source of information about MWH** **(all mothers n = 2381)**								
Heard from chief	2.3%	0.0%**	0.2%***	5.00%***	3.30%	1.50%	0.00%	0.00%
Heard from family member	19.6%	23.9%	26.4%***	21.00%	10.30%***	19.10%	0.00%***	2.30%***
Heard from health care worker	74.4%	72.0%	83.2%***	82.40%*	66.90%***	81.40%*	0.00%***	7.00%***
Heard from headmen	9.0%	5.3%*	8.4%	12.50%***	11.20%	5.70%	0.00%**	2.30%
Heard from community member	23.3%	30.5%**	28.5%**	22.10%	19.10%*	23.20%	0.00%***	4.70%**
Heard from another mother	13.6%	17.7%	17.8%**	15.30%	8.50%**	8.20%*	0.00%***	2.30%*
Heard from radio	6.0%	2.9%	5.7%	8.70%**	7.90%	2.10%*	0.00%	2.30%
Heard from Safe Motherhood Action Group Member	38.1%	13.2%***	26.2%***	57.10%***	57.10%***	28.90%**	0.00%***	9.30%***
Heard from birth attendant	13.3%	14.4%	14.3%	14.60%	14.60%	9.30%	0.00%***	4.70%
**Reasons for not using a MWH (mothers who did not use MWH n = 1622)**								
No MWH	34.30%	23.40%*	28.60%*	33.30%	52.90%***	20.70%***	--	--
No permission from husband/family	1.90%	1.30%	1.90%	1.50%	3.50%	0.00%	--	--
No money	4.30%	3.90%	7.60%**	3.00%	3.50%	2.70%	--	--
Poor quality MWH	1.60%	0.00%	5.20%***	1.10%	0.00%*	0.00%	--	--

*p < .05.

**p < .01.

***p < .001.

MWH = maternity waiting home; % = percent; (SE) = Standard Error; — = no mother in the district responded to this question. All analyses use χ2 or independent means t-tests (Fisher's Exact Test was used for analyses with small cell counts). All analyses compare individual districts with the remaining districts combined. For instance, women who used mother shelters in Choma were compared to all other women who used mother shelters in the other six districts.

## Discussion

Although no standardized model of MWHs yet exists in Zambia, over a third of all women delivering at a health care facility within the seven SMGL rural districts included in the survey used a MWH prior to delivery. Household characteristics of the study population reflect those of the general population in rural Zambia [20].

Mothers who lived 15 km or greater from a health care facility were more likely to use a MWH than women who lived within 9.5–9.9 km from a health care facility. These findings are similar to those from a study of MWH use for high-risk referrals at a district level hospital in Zambia, reporting an average distance of 22 km from place of residence to hospital for 218 MWH users [9]. Our findings also corroborate those of Mramba et al. [21] who found the majority of women (n = 327) who used one MWH in Kenya lived at a distance of greater than 20 km. In contrast, a study conducted in Timor-Leste of MWH use, found the majority of women in two rural districts, 80% and 62% respectively, lived within 5 km of a health center [10]. One reason our findings may vary from these results is that our sample is only among those women living greater than 9.5 km from the health facility. Additionally, a study of 615 women referred to a MWH for high risk conditions in Ethiopia traveled an average distance of 42 km [22].

Unmarried mothers in our study had lower odds of MWH use when compared to married women. This finding could be related to the social stigma women face with premarital pregnancies in Zambia, [23] although future research is needed to examine the relationship between non-marital status and MWH use more thoroughly.

Overall, only 67% of all births take place in a health care facility throughout Zambia; [13] yet, 81% of women surveyed reported delivering at a health care facility. This high prevalence of facility use in our sample is most likely due to the significant interventions associated with the SMGL partnership in the seven districts; including community mobilization and sensitization messages encouraging facility delivery. This is further supported by the fact that the lowest number of facility deliveries out of the seven districts occurred in Pemba and Choma [24]. Both districts are in the “second phase” of the SMGL initiative and have not had sufficient time to reflect the benefits of the program. Saving Mothers, Giving Life has had unprecedented success with a reported 38% reduction in maternal mortality in target SMGL facilities in Zambia [15]. However, it should be noted that the lowest MWH use was reported from two SMGL phase one districts, Mansa and Chembe.

While overall women were satisfied with their stay at a MWH, over half noted crowdedness and nearly a third reported problems with the physical quality of the MWH as well as with their interaction with staff. Again, this reflects earlier findings that women were satisfied with their MWH stays but that areas for improvement included the physical characteristics and resources [11,25].

### Limitations

While this study employs a robust study design, it is limited by its focus on the SMGL districts and this constrains generalization. An analysis of MWH use in communities that have not been sensitized by the SMGL program may have elicited very different results. Furthermore, the initial focus was on communities 10 km or farther from a health facility; however, once geo-coding was completed it was determined that nearly 13% of the communities in the identified districts were less than 10 km from a health facility (thus households 9.5 km or farther were incorporated into the final analysis). While geo-location is commonly used to incorporate geographic and spatial data into a visual analysis, there are limitations when considering the topography of a location. Additionally, pregnancy complications and obstetric risk was not examined in the survey. Finally, the limitation of obtaining data through a cross-sectional analysis of self-reported behavior from up to 13 months prior allows for the potential of social desirability and recall bias.

## Conclusion

This study is the first to describe the prevalence of MWH use and characteristics of the women using health care facilities for birth and choosing to stay at a MWH in seven SMGL districts of rural Zambia. The results presented here show success in districts that are part of the SMGL initiative with higher rates of facility delivery than the general population of rural Zambia.

Maternity waiting homes, which currently take many forms in Zambia, are being used by over a third of women delivering at a health facility in our study. These MWHs appear to be bridging the distance barrier for women who live greater than 9.5 km from a health care facility.

Areas for further improvement include the physical structure of MWHs to accommodate more women in a high quality facility as well as the opportunity to provide educational sessions on a myriad of topics such as newborn care or family planning while women are awaiting childbirth. Additionally, our findings highlight the need to examine the stigma associated with premarital pregnancy and develop opportunities for unmarried women to participate more fully in the health care system.

Maternity waiting homes are one strategy to improve access to skilled birth attendants for women living the greatest distance from a health care facility. Women in these seven districts in Zambia have received messages promoting facility birth and are seeking care within the health system structures. As the quality of both the MWH structures and the care received at the health care facility improves, MWHs have the potential to serve many more women and contribute to the improvement of maternal and newborn outcomes in rural Zambia.

## Supporting information

S1 File Survey Questions English. Household Survey Baseline Impact Evaluation–English.
(PDF)

S2 File Survey Questions in Tonga. Household Survey Baseline Impact Evaluation–Tonga.
(PDF)

S3 File Survey Questions in Bemba. Household Survey Baseline Impact Evaluation–Bemba.
(PDF)

S4 File Survey Questions in Tumbuka. Household Survey Baseline Impact Evaluation–Tumbuka.
(PDF)

S5 File Survey Questions in Nyanja. Household Survey Baseline Impact Evaluation–Nyanja.
(PDF)
