# Supplementary material for: Characteristics of maternity waiting homes and the women who use them: Findings from a baseline cross-sectional household survey among SMGL-supported districts in Zambia
Source: PLoS One. 2018 Dec 31;13(12):e0209815. doi: 10.1371/journal.pone.0209815 (PMC6312364; doi:10.1371/journal.pone.0209815)
Supplement: S1 File — Household Survey Baseline Impact Evaluation–English. (PDF) [file pone.0209815.s001.pdf]

								SURVEY ID

Instrument ID:

The MAHMAZ Project
Baseline Impact Evaluation – Household Survey **ENGLISH**

Target Audience:
Women who have delivered a child in the last 12 months, who are ≥ 15 years of age, and who live within the study facility catchment areas

SHORT SCREEN

SS1	How many women aged 15 to 49 years usually live in this household, including those who passed away or moved away in the last 12 months?<br><br>*Write down the number of women.*	☐☐	If none, thank person and move to next household.
SS2	Have any of these women delivered a baby in the last 12 months, regardless of infant's or mother's health outcome?	YES (1)<br>NO (0)<br>DON'T KNOW (96)	If (0) or (96), thank person and move to next household
SS3	If a woman in the household is unavailable, would you be able to answer questions about her pregnancy and delivery on her behalf?	YES (1)<br>NO (0)<br>DON'T KNOW (96)	
	**INTERVIEWER: IF YOU HAVE ANSWERED YES TO SS1 AND YES TO SS2, THEN PROCEED WITH THE INFORMED CONSENTING PROCESS. PLACE THE UNIQUE ID STICKER ON THE INSTRUMENT AND ON THE HOUSEHOLD CONSENT FORM A.**		
	Confirm consent was granted<br><br>*Draw a check mark if consent was granted.*		
	**IF CONSENT WAS GRANTED, PLACE A SECOND UNIQUE ID STICKER ON THE PAPER VERSION OF THE INSTRUMENT.**		

SURVEY ID

MODULE A. LOCATION

INSTRUCTIONS: Complete before administering the rest of the survey

NO.	FIELD	CODE	RESPONSE
A1	Province	EASTERN (1)<br>SOUTHERN (2)<br>LUAPULA (3)	
A2	District	CHOMA (1)<br>KALOMO (2)<br>PEMBA (3)<br>LUNDAZI (4)<br>NYIMBA (5)<br>MANSA (6)<br>CHEMBE (7)	
A3	Health Facility Catchment Area	CHOMA DISTRICT<br>CHOMA GENERAL (801001)<br>MANGUNZA (801019)<br>MACHA MISSION (801002)<br>MASUKU MISSION (801021)<br>MBABALA (801022)<br>MOCHIPAPA (801023)<br>SIMAKUTU (801043)<br>KALOMO DISTRICT<br>CHIFUSA HC (804023)<br>CHILALA HC (804024)<br>DIMBWE HC (804019)<br>HABULILE HC (804032)<br>KALOMO DISTRICT HOSPITAL (804002)<br>KANCHELE HC (804014)<br>MAWAYA HC (804034)<br>MOONDE HP (804042)<br>MUKWELA HC (804020)<br>SIACHITEMA HC (804013)<br>PEMBA DISTRICT<br>JEMBO (801413)<br>MUZOKA (801419)<br>NYIMBA DISTRICT<br>CHIPEMBE RHC (307010)<br>HOFMEYR ZONAL HC (307011)<br>KACHOLOLA RHC (307012)<br>MKOPEKA RHC (307016)<br>NYIMBA DISTRICT HOSPITAL (307001)<br>MANSA DISTRICT<br>FIMPULU (403017)<br>KABUNDA (403018)<br>LUBENDE (403041)<br>MANO (403026)<br>MANSA GENERAL HOSPITAL (403001)<br>MIBENGE (403029)<br>MUSAILA (403030)<br>MUTITI (403031)	

		MUWANGUNI (403032)<br>CHEMBE DISTRICT<br>KUNDAMFUMU (403023)<br>LUKOLA (403037)<br>LUNDAZI DISTRICT<br>CHIKOMENI (405026)<br>KAMSARO (305034)<br>KAPICHILA (305023)<br>LUKWISIZI (305040)<br>LUNDAZI HOSPITAL (305032)<br>LUSUNTHA (305021)<br>MWASE LUNDAZI ZONAL (305011)<br>NKHANGA (305046)<br>NYANGWE (305020)<br>PHIKAMALAZA (305031)<br>ZUMWANDA (305024)	
A4	Village Name<br><br>*Write in the name of the village.*		
**GPS COORDINATES, TAKE 1**			
A5	Latitude		
A6	Longitude		
**GPS COORDINATES, TAKE 2**			
A7	Latitude (decimal format)		
A8	Longitude (decimal format)		
A9	Date of Interview (DD/MM/YYYY)		
A10	Start time of interview<br>(24:00 format)		

SURVEY ID

MODULE B. HOUSEHOLD ENUMERATION

INSTRUCTIONS: Confirm that the person who you are speaking with is the head of the household or the head woman of the household.

INTERVIEWER: *"I am now going to ask you some basic information about you and the members of your household. For the purposes of the following questions, let us define a household as a group of related or unrelated people who usually live together on a premise, acknowledge the same person as the head of the household, and who have a common cooking and eating arrangement."*

NO.	QUESTION	POTENTIAL RESPONSES	SKIP
B1	How old were you at your last birthday?<br><br>*Unit of response in years.*		
B2	Have you ever attended school?	YES (1)<br>NO (0)<br>DON'T KNOW (96)	If (0) or (96), skip to B4
B3	What is the highest grade you completed?<br><br>*Write grade level (ie: 03 for grade 3).*<br>*If <1 year completed, write down 00.*<br>*If >12 years completed, write down 13.*	DON'T KNOW (96)	
B4	What is your religion?	CATHOLIC (1)<br>PROTESTANT (2)<br>MUSLIM (3)<br>OTHER (SPECIFY) (4):	
B5	What tribe do you belong to?		
B6	What is your marital status?	MARRIED/COHABITING (1)<br>DIVORCED (2)<br>SEPARATED (3)<br>WIDOWED (4)<br>NEVER-MARRIED (5)	If (2), (3), (4), or (5), skip to B8
B7	*If respondent is the male head of household:*<br>In total, how many wives or partners do you have?<br><br>*If respondent is NOT male head of household:*<br>In total, how many wives or partners do the male head of household have?		
B8	How many boys and girls under the age of 5 usually live in your household?<br><br>*Include children who are in boarding school at the moment.*<br>*If none, write down 00.*	BOYS<br>GIRLS	

B9	How many boys and girls between 5 and 14 years of age usually live in your household?<br><br>*Include children who are in boarding school at the moment.*<br>*If none, write down 00.*	BOYS<br>GIRLS	
B10	Including yourself (if applicable), how many men and women between 15 and 49 years of age usually live in your household?	MEN<br>WOMEN	
B11	Including yourself (if applicable), how many men and women between 50 and 64 years of age usually live in your household?	MEN<br>WOMEN	
B12	Including yourself (if applicable), how many men and women 65 years or older usually live in your household?	MEN<br>WOMEN	
**INSTRUCTIONS:** Count and record the total number (B8 to B12) of household members.			
B13	Confirm with respondent: So there are a total of (number) people in your household?	YES (1)<br>NO (0)	

SURVEY ID

INSTRUCTIONS: Ask the respondent to list the names of all women aged 15-49 who usually live in the household, including those who passed away in the last 12 months. Emphasize that you are also looking for information on individuals who have passed away in the last 12 months. Fill out column A with all names provided, and then continue to answer B-F for each person before selecting a respondent.

INTERVIEWER: *"Now I would like you to list for me the names of all women who usually live in this household that are between the ages of 15 and 49 years. Please include the names of women who have passed away in the 12 months."*

TABLE 1. ROSTER OF WOMEN AGED 15-49 YEARS

	A. Please tell me the first name of all women between the ages of 15 and 49 years that usually live in this household for at least 4 days a week, **including those that have passed away in the last 12 months**.<br><br>Ensure the number includes those who would have been living there if they didn't pass away/move away in the past 12 months.<br>*The number of women in this list should be greater or equal to the number of women in* ***B10****.*	B. In the last 12 months, did (name) have a pregnancy that lasted at least 35 weeks?<br><br>*That is, was (name) pregnant at least up until ~3 weeks before her estimated delivery date?*<br><br>*Instructions: Note that this includes a delivery, still birth, neonatal death, etc.at any point within the past 12 months*<br><br>YES (1)<br>NO (0)<br>DON'T KNOW (96)<br><br>*If (0) or (96), skip to next person.*	C. Is (*name) still alive?<br><br>YES (1)<br>NO/DON'T KNOW(0)<br><br>*If (1), skip to E*<br><br>**Check list and input name*	D. Are you or somebody else willing to answer a few questions about (name)'s pregnancy on her behalf?<br><br>YES (1)<br>NO (0)<br><br>*If (0), skip to next person.*	E. Is (*name) potentially eligible to take the survey?<br><br>*If* ***B=1*** *and (****IF APPLICABLE)******D=1****, mark the box below.*<br><br>**Check list and input name*	**AFTER ALL WOMEN HAVE BEEN LISTED, TO SELECT A RESPONDENT:**<br><br>1. Roll the die<br>2. From the 1st checked box in Column E, count up to the rolled number, beginning again at the 1st checked box if needed until number is reached<br>3. Roll the die again<br>4. From the checked box you landed on after the 1st roll, count up to the 2nd rolled number, beginning again at the 1st checked box if needed until the 2nd number is reached<br>5. Select this woman<br>6. If woman selected is ALIVE, proceed to **Question B24**<br>7. If woman selected is DECEASED, proceed to Proxy Household Survey
B14					☐	
B15					☐	
B16					☐	
B17					☐	
B18					☐	
B19					☐	
B20					☐	
B21					☐	
B22					☐	
B23					☐	

SURVEY ID

NO.	QUESTION	POTENTIAL RESPONSES	SKIP
B23A	How old is (name)?		
B24	Is (name) available to take the survey?	YES (1)<br>NO (0)<br>DON'T KNOW (96)	If (1), skip to consent then proceed to B27
B25	Can we schedule a time to come back when she will be available?	YES (1)<br>NO (0)<br>DON'T KNOW (96)	If (0) or (96), resample from potentially eligible women in TABLE 1
B26	Were you able to reschedule another time?	YES (1)<br>NO (0)	If (0), resample from potentially eligible women in TABLE 1
If you are unable to reschedule a time to come back and survey the sampled woman, go back to TABLE 1 and resample another potentially eligible woman. If you are re-visiting the household a subsequent time and the woman is now available, **proceed starting with Question B27**.			

INSTRUCTIONS: Make sure to obtain consent or assent (if the sampled woman is 15, 16 or 17 years old – refer to B23A), including a signature, from the sampled woman. If the woman is not able to sign, please have the woman provide a thumbprint. These questions will determine whether or not the sampled woman is eligible to proceed to the full household survey. If she is ineligible, then re-sample from Roster Table 1. If there are no more potentially eligible women to sample from, thank the woman and move on to the next household. If she is eligible, proceed to Module C.

STOP: MAKE SURE CONSENT OR ASSENT WAS OBTAINED FROM (NAME). PLACE A THIRD UNIQUE ID STICKER ON THE CONSENT FORM B – FOR THE ELIGBLE WOMAN.

INTERVIEWER: "Thank you for agreeing to take our survey. Now I am going to ask you a few questions about your most recent delivery."

NO.	QUESTION	POTENTIAL RESPONSES	SKIP
B27	Is the baby still alive?<br><br>*Soften the question by asking: Is your baby around?*	YES (1)<br>NO (0)<br>DON'T KNOW (96)	If (1), continue to Module C<br>If (96), skip to B29
B28	When did your baby die?	BEFORE OR ON DAY OF DELIVERY (1)<br>WITHIN ONE MONTH AFTER DELIVERY (2)<br>OTHER (3)	If (2) or (3), continue to Module C
B29	Did you deliver your baby before your estimated delivery date (EDD)?	YES (1)<br>NO (0)<br>DON'T KNOW (96)	If (0), continue to Module C<br>If (96), end and re-sample from Roster Table 1
B30	How many weeks before your estimated delivery date (EDD) did you deliver your baby?	<= 3 WEEKS (1)<br>>3 WEEKS (2)<br>DON'T KNOW (96)	If (1), continue to Module C<br>If (2), end and re-sample from Roster Table 1<br>If 96, end and re-sample from Roster Table 1

SURVEY ID

MODULE C. DEMOGRAPHICS

INSTRUCTIONS: After eligible respondent has been randomly sampled from all eligible respondents, proceed with the instrument. Ensure that the woman selected to proceed with the survey has delivered a child **within the last year**. This section is to get basic demographics on the household and the respondent.

***INTERVIEWER:** "I am now going to ask you some questions about yourself and your household."*

NO.	QUESTION	POTENTIAL RESPONSES	SKIP
C1	Are you the head of the household?	YES (1)<br>NO (0)	If (1), skip to C9
C2	What is your relationship to the head of household?	SPOUSE (1)<br>CHILD (2)<br>GRANDCHILD (3)<br>NIECE (4)<br>AUNTIE/OTHER RELATIVE (5)<br>OTHER (SPECIFY) (6):	
C3	Have you ever attended school?	YES (1)<br>NO (0)<br>DON'T KNOW (96)	If (0) or (96), skip to C5
C4	What is the highest grade you completed?<br><br>*If <1 year completed, write down 00. If >12 years completed, write down 13.*	DON'T KNOW (96)	
C5	What is your religion?	CATHOLIC (1)<br>PROTESTANT (2)<br>MUSLIM (3)<br>OTHER (SPECIFY) (4):	
C6	What tribe do you belong to?		
C7	What is your marital status?	MARRIED/COHABITING (1)<br>DIVORCED (2)<br>SEPARATED (3)<br>WIDOWED (4)<br>NEVER-MARRIED (5)	If (2), (3), (4) or (5), skip to C9
C8	In total, how many wives or partners, including yourself, does your husband have?<br><br>*If don't know, write down 96.*		
C9	How many times have you ever been pregnant?		
C10	How many live births have you had?		

***INTERVIEWER:** "Now let's discuss your house."*

								SURVEY ID

NO.	QUESTION	POTENTIAL RESPONSES	SKIP
C11	What is the **main** source of drinking water for members of your household?	*PIPED WATER*<br>PIPED INTO DWELLING (1)<br>PIPED TO YARD/PLOT (2)<br>PUBLIC TAP/STANDPIPE (3)<br>TUBE WELL OR BOREHOLE (4)<br>*DUG WELL*<br>PROTECTED WELL (5)<br>UNPROTETED WELL (6)<br>*WATER FROM SPRING*<br>PROTECTED SPRING (7)<br>UNPROTECTED SPRING (8)<br>RAINWATER (9)<br>TANKER TRUCK (10)<br>CART WITH SMALL TANK (11)<br>SURFACE WATER (12)<br>(RIVER/DAM/LAKE/POND/STREAM/CANAL/ IRRIGATION CHANNEL)<br>BOTTLED WATER (13)<br>OTHER (PLEASE SPECIFY) (14):	If (13), skip to C14
C12	Where is that water source located?	IN OWN DWELLING (1)<br>IN OWN YARD/PLOT (2)<br>ELSEWHERE (3)	If (1) or (2), skip to C14
C13	How long does it take to go there, get water, and come back?	MINUTES: ☐☐☐<br>DON'T KNOW (96)	
C14	Do you do anything to the water to make it safer to drink?	YES (1)<br>NO (0)<br>DON'T KNOW (96)	
C15	What kind of toilet facility do members of your household usually use?	*FLUSH OR POUR FLUSH TOILET*<br>FLUSH TO PIPED SEWER SYSTEM (1)<br>FLUSH TO SEPTIC TANK (2)<br>FLUSH TO PIT LATRINE (3)<br>FLUSH TO SOMEWHERE ELSE (4)<br>FLUSH, DON'T KNOW WHERE (5)<br>*PIT LATRINE*<br>VENTILATED IMPROVED PIT LATRINE (6)<br>PIT LATRINE WITH SLAB (7)<br>PIT LATRINE WITHOUT SLAB/OPEN PIT (8)<br>COMPOSTING TOILET (9)<br>BUCKET TOILET (10)<br>HANGING TOILET/HANGING LATRINE (11)<br>NO FACILITY/BUSH/FIELD (12)<br>OTHER (SPECIFY) (13):	
C16	Do you share this toilet facility with other households?	YES (1)<br>NO (0)	

C17	Does your household have any of the following (item must be functioning usually):		**YES (1)**	**NO (0)**	**DON'T KNOW (96)**
	A	ELECTRICITY	☐	☐	☐
	B	SOLAR POWER	☐	☐	☐
	C	GENERATOR	☐	☐	☐

D	PARAFFIN LAMP	☐	☐	☐
E	REFRIGERATOR	☐	☐	☐
F	MICROWAVE	☐	☐	☐
G	CHARCOAL STOVE	☐	☐	☐
H	WOOD STOVE	☐	☐	☐
I	ELECTRIC STOVE	☐	☐	☐
J	BED	☐	☐	☐
K	MATTRESS	☐	☐	☐
L	CHAIR	☐	☐	☐
M	TABLE	☐	☐	☐
N	CUPBOARD	☐	☐	☐
O	SOFA	☐	☐	☐
P	CLOCK	☐	☐	☐
Q	FAN	☐	☐	☐
R	SEWING MACHINE	☐	☐	☐
S	MOSQUITO NET	☐	☐	☐
T	INTERNET	☐	☐	☐
U	WATCH	☐	☐	☐
V	BANK/SAVINGS ACCOUNT	☐	☐	☐
W	PLOUGH	☐	☐	☐
X	WHEELBARROW	☐	☐	☐
Y	GRAIN GRINDER	☐	☐	☐
Z	TRACTOR	☐	☐	☐
AA	HAMMER MILL	☐	☐	☐
BB	SHOVEL	☐	☐	☐
CC	MACHETE	☐	☐	☐
DD	PICKAXE	☐	☐	☐
EE	WATER PUMP	☐	☐	☐
FF	FARM LAND	☐	☐	☐
GG	TREES THAT PRODUCE FRUIT	☐	☐	☐
HH	RADIO	☐	☐	☐
II	TELEVISION	☐	☐	☐
JJ	MOBILE TELEPHONE	☐	☐	☐
KK	NON-MOBILE TELEPHONE	☐	☐	☐
LL	COMPUTER	☐	☐	☐
MM	CASSETTE	☐	☐	☐

	PLAYER			
NN	VCR/DVD	☐	☐	☐
OO	BICYCLE	☐	☐	☐
PP	MOTORCYCLE/MOTOR SCOOTER	☐	☐	☐
QQ	ANIMAL-DRAWN CART	☐	☐	☐
RR	CAR/TRUCK	☐	☐	☐
SS	BOAT WITH A MOTOR	☐	☐	☐
TT	BANANA BOAT	☐	☐	☐

C18	What type of fuel does your household mainly use for cooking?	ELECTRICITY (1)<br>SOLAR POWER (2)<br>LIQUID PROPANE GAS (LPG) (3)<br>NATURAL GAS (4)<br>BIOGAS (5)<br>KEROSENE (6)<br>COAL, LIGNITE (7)<br>CHARCOAL (8)<br>WOOD (9)<br>STRAW/SHRUBS/GRASS (10)<br>AGRICULTURAL CROP (11)<br>ANIMAL DUNG (12)<br>NO FOOD COOKED IN HOUSEHOLD (13)<br>OTHER (SPECIFY) (14):	If (13), skip to C20
C19	Where is cooking usually done for your household?	IN THE HOUSE (1)<br>IN A SEPARATE BUILDING (2)<br>OUTDOORS (3)<br>OTHER (SPECIFY) (4):	
C20	What material is the floor of your main dwelling made of?<br><br>*OBSERVE THE FLOOR TO CONFIRM. (If more than one material, select the one that is "most" common)*	NATURAL FLOOR<br>EARTH/SAND (1)<br>DUNG (2)<br>RUDIMENTARY FLOOR<br>WOOD PLANKS (3)<br>PALM/BAMBOO/REEDS (4)<br>FINISHED FLOOR<br>PARQUET/POLISHED WOOD (5)<br>VINYL (PVC) OR ASPHALT STRIPS (6)<br>CERAMIC/TERRAZZO TILES (7)<br>CONCRETE CEMENT (8)<br>CARPET (9)<br>OTHER (SPECIFY) (10):	
C21	What material is the roof of your main dwelling made of?<br><br>*OBSERVE THE ROOF TO CONFIRM. (If more than one material, select the one that is "most" common)*	*NATURAL ROOFING*<br>NO ROOF (0)<br>THATCH/PALM LEAF (1)<br>*RUDIMENTARY ROOFING*<br>RUSTIC MAT (2)<br>PALM/BAMBOO (3)<br>WOOD PLANKS (4)<br>CARDBOARD (5)<br>*FINISHED ROOFING*<br>METAL/IRON SHEETS (6)<br>WOOD (7)<br>CALAMINE/CEMENT FIBRE (ASBESTOS) (8)<br>CERAMIC/HARVEY TILES (9)	

SURVEY ID

		CEMENT (10)<br>ROOFING SHINGLES (11)<br>MUD TILES (12)<br>OTHER (SPECIFY) (13):	
C22	What is the primary construction material of the housing unit's exterior walls?<br><br>*OBSERVE THE WALLS TO CONFIRM. (If more than one material, select the one that is "most" common)*	*NATURAL WALLS*<br>NO WALLS (0)<br>CANE/PALM/TRUNKS (1)<br>MUD (2)<br>*RUDIMENTARY WALLS*<br>BAMBOO/POLE WITH MUD (3)<br>STONE WITH MUD (4)<br>PLYWOOD (5)<br>CARDBOARD (6)<br>REUSED WOOD (7)<br>*FINISHED WALLS*<br>CEMENT (8)<br>STONE WITH LIME/CEMENT (9)<br>BRICK (10)<br>CEMENT BLOCKS (11)<br>WOOD PLANKS (12)<br>OTHER (SPECIFY) (13):	
C23	Does any member of your household own agricultural land?	YES (1)<br>NO (0)<br>DON'T KNOW (96)	If (0) or (96), skip to C25
C24	How many lima, acres, hectares or square meters of agricultural land do members of this household own?	LIMA (1)<br>ACRES (2)<br>HECTARES (3)<br>SQUARE METERS (4)<br>DON'T KNOW (96)<br>QUANTITY	

C25	How many of the following animals does your household own?	NUMBER	NONE (00)	DON'T KNOW (96)
A	TRADITIONAL CATTLE		☐	☐
B	DAIRY CATTLE		☐	☐
C	BEEF CATTLE		☐	☐
D	HORSES/DONKEYS/MULES		☐	☐
E	GOATS		☐	☐
F	SHEEP		☐	☐
G	PIGS		☐	☐
H	CHICKENS/OTHER POULTRY		☐	☐

I	RABBITS		☐	☐
J	OTHER LIVESTOCK		☐	☐

NO.	QUESTION	POTENTIAL RESPONSES	SKIP
C26	Please indicate all of the regular sources of household income in the last 12 months.<br><br>*Select all that apply.*	SALARIED EMPLOYMENT (1)<br>SMALL BUSINESS, SHOP OR KIOSK (2)<br>SMALL HOUSEHOLD INCOME GENERATING ACTIVITY (3)<br>DOWRY (4)<br>SALE OF CROPS/ANIMALS (5)<br>SALE OF ASSETS (6)<br>REMITTANCES (CASH DONATIONS FROM FRIENDS/FAMILY) (7)<br>GOVERNMENT/NGO AID, GRANT OR OTHER FINANCIAL SUPPORT (8)<br>CASUAL DAILY WORK (9)<br>OTHER (SPECIFY) (10):	
C27	If your household wanted to borrow money from a bank or other financial service provider (not including friends or relatives), would your household be able to borrow money?	NO (0)<br>PROBABLY NOT (1)<br>PROBABLY YES (2)<br>DEFINITELY YES (3)<br>DON'T KNOW (96)	
C28	Did all pregnant women and children <5 years of age sleep under a mosquito net last night?	YES (1)<br>NO (0)<br>DON'T KNOW (96)	
C29	Can your household afford your children's school fees and school supplies?	YES (1)<br>USUALLY (2)<br>SOMETIMES (3)<br>RARELY (4)<br>OTHER (SPECIFY) (5):<br>NO (0)	
C30	In the last month, did anyone in your household go a whole day and night without eating?	YES (1)<br>NO (0)<br>DON'T KNOW (96)	
C31	Did any of the children in your household go to bed hungry last night?	YES (1)<br>NO (0)<br>DON'T KNOW (96)	
C32	Can your home withstand strong winds or severe rain without significant damage?	YES (1)<br>NO (0)<br>DON'T KNOW (96)	
C33	When it rains, does any water leak into the part of the house where the children sleep?	YES (1)<br>NO (0)<br>DON'T KNOW (96)	

SURVEY ID

MODULE D. LAST DELIVERY/MOTHERS' SHELTER

***INTERVIEWER:** "Now I'm going to ask you some questions that are specific to your most recent delivery. First, take a moment to think about the time just before your most recent delivery and your delivery itself. Then we will discuss mothers' shelters. Are you ready to begin?"*

NO.	QUESTION	POTENTIAL RESPONSES	SKIP
D1	When was your most recent delivery? (DD MONTH YYYY)<br><br>*If date not known, ask for under 5 card. If no under 5 card, write 15th*	D D M M Y Y Y Y	
D2	Before I came here today, have you ever heard of a mothers' shelter?	YES (1)<br>NO (0)<br>DON'T KNOW (96)	If (0) or (96), skip to D18
D3	From where/whom did you hear about mothers' shelters?<br><br>*Select all that apply.*	CHIEF (1)<br>HEADMEN (2)<br>HEALTH CARE WORKER (3)<br>SMAG (4)<br>TRADITIONAL BIRTH ATTENDANT (5)<br>FAMILY MEMBER (6)<br>ANOTHER MOTHER (7)<br>OTHER COMMUNITY MEMBER (8)<br>RADIO (9)<br>OTHER (SPECIFY) (10):	
D4	Thinking about the delivery we've been talking about, did you stay at a mothers' shelter for any reason at all before or after that delivery?	YES (1)<br>NO (0)	If (1), skip to D5
D4a	If NO, why?<br><br>*Select all that apply.*	NO MOTHERS SHELTER (1)<br>NO PERMISSION FROM HUSBAND OR FAMILY (2)<br>NO MONEY (3)<br>POOR QUALITY (4)<br>NOT CLEAN (5)<br>TOO CROWDED (6)<br>NOT CULTURALLY APPROPRIATE (7)<br>NOT SAFE (8)<br>DELAYS DELIVERY (9)<br>DIDN'T KNOW ABOUT MOTHERS SHELTER (10)<br>OTHER (SPECIFY) (11):	Skip to D18

INSTRUCTIONS: Ask the respondent for what reason(s) did she stay at a mothers' shelter, and then prompt her with the reasons listed below.

SURVEY ID

D5	For about how many nights did you stay at the mothers' shelter for the following reason(s)?	NUMBER OF NIGHTS	NONE (0)	DON'T KNOW (96)
A	1ST ANC VISIT	☐☐	☐	☐
B	OTHER ANC VISIT(S)	☐☐	☐	☐
C	WHILE AWAITING DELIVERY	☐☐	☐	☐
D	AFTER DISCHARGE FROM HEALTH FACILITY/ IMMEDIATELY AFTER DELIVERY	☐☐	☐	☐
E	3 DAY POST NATAL VISIT	☐☐	☐	☐
F	7-14 DAYS POST NATAL VISIT	☐☐	☐	☐
G	6 WEEK POST NATAL VISIT	☐☐	☐	☐
H	OTHER (SPECIFY)	☐☐	☐	☐

NO.	QUESTION	POTENTIAL RESPONSES	SKIP
D6	Which mothers' shelter did you stay at for the time you stayed the longest at a mothers' shelter?<br><br>*Confirm the longest number of nights the respondent stayed at a mothers' shelter.*	CHOMA DISTRICT<br>CHOMA GENERAL (801001)<br>MANGUNZA (801019)<br>MACHA MISSION (801002)<br>MASUKU MISSION (801021)<br>MBABALA (801022)<br>MOCHIPAPA (801023)<br>SIMAKUTU (801043)<br>KALOMO DISTRICT<br>CHIFUSA HC (804023)<br>CHILALA HC (804024)<br>DIMBWE HC (804019)<br>HABULILE HC (804032)<br>KALOMO DISTRICT HOSPITAL (804002)<br>KANCHELE HC (804014)<br>MAWAYA HC (804034)<br>MOONDE HP (804042)<br>MUKWELA HC (804020)<br>SIACHITEMA HC (804013)<br>PEMBA DISTRICT<br>JEMBO (801413)<br>MUZOKA (801419)<br>NYIMBA DISTRICT<br>CHIPEMBE RHC (307010)<br>HOFMEYR ZONAL HC (307011)	

		KACHOLOLA RHC (307012)<br>MKOPEKA RHC (307016)<br>NYIMBA DISTRICT HOSPITAL (307001)<br>MANSA DISTRICT<br>FIMPULU (403017)<br>KABUNDA (403018)<br>LUBENDE (403041)<br>MANO (403026)<br>MANSA GENERAL HOSPITAL (403001)<br>MIBENGE (403029)<br>MUSAILA (403030)<br>MUTITI (403031)<br>MUWANGUNI (403032)<br>CHEMBE DISTRICT<br>KUNDAMFUMU (403023)<br>LUKOLA (403037)<br>LUNDAZI DISTRICT<br>CHIKOMENI (405026)<br>KAMSARO (305034)<br>KAPICHILA (305023)<br>LUKWISIZI (305040)<br>LUNDAZI HOSPITAL (305032)<br>LUSUNTHA (305021)<br>MWASE LUNDAZI ZONAL (305011)<br>NKHANGA (305046)<br>NYANGWE (305020)<br>PHIKAMALAZA (305031)<br>ZUMWANDA (305024)<br>OTHER (SPECIFY NAME OF HEALTH FACILITY AND DISTRICT) (47):	

INTERVIEWER: *"Now I am going to ask you about your experience at the mothers' shelter. Take some time to think about your stay there. Are you ready to begin?"*

D7		During your stay at the mothers' shelter…	**YES (1)**	**NO (0)**	**DON'T KNOW (96)**
	A	WAS THERE A BED OR MATTRESS AVAILABLE TO YOU	☐	☐	☐
	B	DID YOU HAVE TO SHARE A BED/MATTRESS WITH ANOTHER PERSON AT ANY TIME	☐	☐	☐
	C	DID YOU EVER SLEEP UNDER A MOSQUITO NET AT NIGHTTIME	☐	☐	☐
	D	WERE YOU ORIENTED TO THE RULES AND PROCEDURES OF THE MOTHERS' SHELTER UPON ARRIVAL	☐	☐	☐
	E	DID YOU HAVE ACCESS TO CLEAN WATER	☐	☐	☐
	F	DID YOU HAVE A SOURCE OF LIGHT AFTER THE SUN WENT DOWN	☐	☐	☐
	G	DID YOU HAVE ACCESS TO A	☐	☐	☐

	BATHING/LAUNDRY AREA			
H	WAS THERE A SAFE SPACE TO PUT YOUR PERSONAL BELONGINGS, INCLUDING FOOD	☐	☐	☐
I	DID YOU ATTEND ANY HEALTH EDUCATION SESSIONS	☐	☐	☐

NO.	QUESTION	POTENTIAL RESPONSES	SKIP
D8	Was there a designated cooking space at the mothers' shelter?	YES (1)<br>NO (0)<br>DON'T KNOW (96)	If (0) or (96), skip to D10
D9	Was the designated cooking space covered?	YES (1)<br>NO (0)<br>DON'T KNOW (96)	
D10	Did you acquire any new skills while at the mothers' shelter?	YES (1)<br>NO (0)<br>DON'T KNOW (96)	If (0) or (96), skip to D12
D11	What skills did you acquire?		
D12	Were you asked to contribute money (kwacha) to stay at the mothers' shelter?	YES (1)<br>NO (0)<br>DON'T KNOW (96)	If (0) or (96), skip to D14
D13	How much money (kwacha) did you contribute in total?		
D14	Were you asked to contribute anything other than money to stay at the mothers' shelter?	YES (1)<br>NO (0)<br>DON'T KNOW (96)	If (0) or (96), skip to D16
D15	What did you contribute other than money?<br><br>*Select all that apply.*	LABOR (1)<br>LIVESTOCK/POULTRY (2)<br>FOOD OR OTHER AGRICULTURAL RESOURCES (3)<br>OTHER IN-KIND RESOURCES (SPECIFY) (4):<br>OTHER (SPECIFY) (5):	

D16	***INTERVIEWER:*** *"Now I'm going to ask you some common problems women face at mothers' shelters while staying there prior to delivery. As I mention each one, please tell me whether any of these were a problem for you during your stay at the mothers' shelter, and if so if they were a major or minor problem for you."*				
		**MAJOR PROBLEM (2)**	**MINOR PROBLEM (1)**	**NO PROBLEM (0)**	**UNDECIDED (96)**
A	OVERALL QUALITY OF THE MOTHERS SHELTER	☐	☐	☐	☐
B	MANAGEMENT AND OVERSIGHT	☐	☐	☐	☐
C	CLEANLINESS OF THE MOTHERS' SHELTER	☐	☐	☐	☐
D	PRESENCE OF STAFF	☐	☐	☐	☐
E	FRIENDLINESS OF STAFF WHILE STAYING AT THE MOTHERS' SHELTER	☐	☐	☐	☐
F	ACCESS TO COOKING AREA	☐	☐	☐	☐

	G CROWDEDNESS OF THE MOTHERS' SHELTER	☐	☐	☐	☐
	H SAFETY WHILE STAYING AT THE MOTHERS' SHELTER	☐	☐	☐	☐
	I BOREDOM WHILE STAYING AT THE MOTHERS' SHELTER	☐	☐	☐	☐
	J CULTURAL APPROPRIATENESS OF THE MOTHERS' SHELTER	☐	☐	☐	☐

NO.	QUESTION	POTENTIAL RESPONSES	SKIP
D17	In general, how satisfied were you with your stay at the mothers' shelter?	VERY SATISFIED (1)<br>MORE OR LESS SATISFIED (2)<br>NOT SATISFIED (3)	
D17a	Do you intend to use mothers' shelter for future deliveries?	YES (1)<br>NO (0)<br>DON'T KNOW (96)	
D17b	Would you recommend using a mothers' shelter to friends or relatives?	YES (1)<br>NO (0)<br>DON'T KNOW (96)	

***INTERVIEWER:* "Thank you for answering questions about mothers' shelters. Now I'd like to discuss your most recent delivery again."**

D18	Who assisted during your last delivery?<br><br>*Select all that apply.*<br><br>*If respondent says NO ONE ASSSISTED, probe to determine whether any adults were present at the delivery.*	DOCTOR/CLINICAL OFFICER (1)<br>NURSE/MIDWIFE (2)<br>OTHER HEALTH FACILITY STAFF/PERSONNEL (3)<br>TRADITIONAL BIRTH ATTENDANT (4)<br>SMAG (5)<br>RELATIVE/FRIEND/AUNTIE (6)<br>NO ONE ASSISTED (7)<br>OTHER (SPECIFY) (8):	
D18a	Where do you intend to deliver your next baby?	YOUR HOME (1)<br>OTHER HOME (2)<br>HEALTH POST/FACILITY (3)<br>HOSPITAL (4)<br>OTHER (SPECIFY) (5):	
D18b	Where did you intend to deliver your last delivery?	YOUR HOME (1)<br>OTHER HOME (2)<br>HEALTH POST/FACILITY (3)<br>HOSPITAL (4)<br>OTHER (SPECIFY) (5):	
D19	Where did you deliver your last baby?	YOUR HOME (1)<br>OTHER HOME (2)<br>HEALTH POST/FACILITY (3)<br>HOSPITAL (4)<br>OTHER (SPECIFY) (5):	If (1), (2) or (5), skip to D31
**INSTRUCTIONS:** If respondent answers **OTHER (5) to Question D19**, probe to ensure this is not a health post/facility or hospital.			

								SURVEY ID

FACILITY-BASED DELIVERY			
**NO.**	**QUESTION**	**POTENTIAL RESPONSES**	**SKIP**
D20	Which health facility did you **FIRST** go to for your last delivery?<br><br>*(**INSTRUCTIONS:** if woman reports hospital, probe to ensure she did not first present at a health facility and was transferred to hospital.)*	CHOMA DISTRICT<br>CHOMA GENERAL (801001)<br>MANGUNZA (801019)<br>MACHA MISSION (801002)<br>MASUKU MISSION (801021)<br>MBABALA (801022)<br>MOCHIPAPA (801023)<br>SIMAKUTU (801043)<br>KALOMO DISTRICT<br>CHIFUSA HC (804023)<br>CHILALA HC (804024)<br>DIMBWE HC (804019)<br>HABULILE HC (804032)<br>KALOMO DISTRICT HOSPITAL (804002)<br>KANCHELE HC (804014)<br>MAWAYA HC (804034)<br>MOONDE HP (804042)<br>MUKWELA HC (804020)<br>SIACHITEMA HC (804013)<br>PEMBA DISTRICT<br>JEMBO (801413)<br>MUZOKA (801419)<br>NYIMBA DISTRICT<br>CHIPEMBE RHC (307010)<br>HOFMEYR ZONAL HC (307011)<br>KACHOLOLA RHC (307012)<br>MKOPEKA RHC (307016)<br>NYIMBA DISTRICT HOSPITAL (307001)<br>MANSA DISTRICT<br>FIMPULU (403017)<br>KABUNDA (403018)<br>LUBENDE (403041)<br>MANO (403026)<br>MANSA GENERAL HOSPITAL (403001)<br>MIBENGE (403029)<br>MUSAILA (403030)<br>MUTITI (403031)<br>MUWANGUNI (403032)<br>CHEMBE DISTRICT<br>KUNDAMFUMU (403023)<br>LUKOLA (403037)<br>LUNDAZI DISTRICT<br>CHIKOMENI (405026)<br>KAMSARO (305034)<br>KAPICHILA (305023)<br>LUKWISIZI (305040)<br>LUNDAZI HOSPITAL (305032)<br>LUSUNTHA (305021)<br>MWASE LUNDAZI ZONAL (305011)<br>NKHANGA (305046)<br>NYANGWE (305020)<br>PHIKAMALAZA (305031)<br>ZUMWANDA (305024)<br>OTHER (SPECIFY NAME OF HEALTH FACILITY AND DISTRICT) (47):	

SURVEY ID

D21	What mode of transportation did you use to get to [*name of facility*] for your last delivery?	WALKING (1)<br>BICYCLE (2)<br>CARRIED IN WHEELBARROW (3)<br>ANIMAL-DRAWN CART (4)<br>TAXI (5)<br>CAR (6)<br>MOTORCYCLE (7)<br>AMBULANCE (8)<br>OTHER (SPECIFY) (9):	
D22	Using *[mode of transportation]*, how long did it take you to get to the health facility?	HOURS MINUTES	
D23	Did you deliver your baby at the health facility you originally went to for your delivery?	YES (1)<br>NO (0)<br>DON'T KNOW (96)	If (1) or (96) skip to D27
D24	What is the name of the health facility/post or hospital you **actually** delivered your baby at?	CHOMA DISTRICT<br>CHOMA GENERAL (801001)<br>MANGUNZA (801019)<br>MACHA MISSION (801002)<br>MASUKU MISSION (801021)<br>MBABALA (801022)<br>MOCHIPAPA (801023)<br>SIMAKUTU (801043)<br>KALOMO DISTRICT<br>CHIFUSA HC (804023)<br>CHILALA HC (804024)<br>DIMBWE HC (804019)<br>HABULILE HC (804032)<br>KALOMO DISTRICT HOSPITAL (804002)<br>KANCHELE HC (804014)<br>MAWAYA HC (804034)<br>MOONDE HP (804042)<br>MUKWELA HC (804020)<br>SIACHITEMA HC (804013)<br>PEMBA DISTRICT<br>JEMBO (801413)<br>MUZOKA (801419)<br>NYIMBA DISTRICT<br>CHIPEMBE RHC (307010)<br>HOFMEYR ZONAL HC (307011)<br>KACHOLOLA RHC (307012)<br>MKOPEKA RHC (307016)<br>NYIMBA DISTRICT HOSPITAL (307001)<br>MANSA DISTRICT<br>FIMPULU (403017)<br>KABUNDA (403018)<br>LUBENDE (403041)<br>MANO (403026)<br>MANSA GENERAL HOSPITAL (403001)<br>MIBENGE (403029)<br>MUSAILA (403030)<br>MUTITI (403031)<br>MUWANGUNI (403032)	

		CHEMBE DISTRICT<br>KUNDAMFUMU (403023)<br>LUKOLA (403037)<br>LUNDAZI DISTRICT<br>CHIKOMENI (405026)<br>KAMSARO (305034)<br>KAPICHILA (305023)<br>LUKWISIZI (305040)<br>LUNDAZI HOSPITAL (305032)<br>LUSUNTHA (305021)<br>MWASE LUNDAZI ZONAL (305011)<br>NKHANGA (305046)<br>NYANGWE (305020)<br>PHIKAMALAZA (305031)<br>ZUMWANDA (305024)<br>OTHER (SPECIFY NAME OF HEALTH FACILITY AND DISTRICT) (47):	
D25	Were you referred or transferred to this facility by a health provider?	YES (1)<br>NO (0)<br>DON'T KNOW (96)	If (0) or (96), skip to D27
D26	Approximately how long did it take you to be transferred to this facility?	LESS THAN 1 HOUR (1)<br>1 TO 2 HOURS (2)<br>MORE THAN 2 HOURS (3)	
D27	Who was the primary decision maker of you delivering at a facility?	YOURSELF (1)<br>HUSBAND/PARTNER (2)<br>MOTHER/MOTHER-IN-LAW (3)<br>AUNTIE (4)<br>OTHER FAMILY MEMBER (5)<br>FRIEND (6)<br>OTHER (SPECIFY) (7):	
D28	Did you stay at the health facility for **at least 24 hours** after delivery before you were discharged?	YES (1)<br>NO (0)<br>DON'T KNOW (96)	

D29	***INTERVIEWER:*** *"I am going to list a number of health care services, and I'd like to know if you did or did not receive each of them at the health care facility during the time of your delivery."*			
		**RECEIVED (1)**	**DID NOT RECEIVE (0)**	**DON'T KNOW (96)**
	A Cesarean section (*belly cut open to deliver child)*	☐	☐	☐
	B Blood transfusion/receive blood	☐	☐	☐
	C Antibiotics/intravenous (IV) drip	☐	☐	☐
	D Counseling on breastfeeding	☐	☐	☐
	E Family planning products/services/counseling	☐	☐	☐
	F Counseling on kangaroo (skin to skin) care of your newborn	☐	☐	☐

D30	***INTERVIEWER:*** *"Now I'm going to ask you some common problems women face at health facilities during their delivery. As I mention each one, please tell me whether any of these were a problem for you during your delivery at the health facility, and if so if they were a major or minor problem for you."*				
		**MAJOR PROBLEM (2)**	**MINOR PROBLEM (1)**	**NO PROBLEM (0)**	**UNDECIDED (96)**
A	QUALITY OF MEDICAL CARE RECEIVED DURING DELIVERY	☐	☐	☐	☐
B	RESPECT SHOWN BY HEALTH CARE WORKERS	☐	☐	☐	☐
C	PRIVACY DURING DELIVERY	☐	☐	☐	☐
D	CLEANLINESS OF HEALTH CARE FACILITY	☐	☐	☐	☐
	**After completing the facility-based delivery section, continue to MODULE E.**				

HOME DELIVERIES

No.	Question	Potential responses	Skip
D31	Who was the primary decision maker of you delivering at home?	YOURSELF (1)<br>HUSBAND/PARTNER (2)<br>MOTHER/MOTHER-IN-LAW (3)<br>AUNTIE (4)<br>OTHER FAMILY MEMBER (5)<br>FRIEND (6)<br>OTHER (SPECIFY) (7):	
D32	What were the main reasons why you did not deliver at a facility?<br><br>*Select all that apply.*	COST TOO MUCH (1)<br>FACILITY NOT OPEN (2)<br>TOO FAR/NO TRANSPORTATION (3)<br>POOR QUALITY SERVICE/DON'T TRUST (4)<br>NO FEMALE HEALTH PROVIDER (5)<br>HUSBAND/FAMILY DIDN'T ALLOW (6)<br>SHORT LABOR (7)<br>BABY CLOTHES (8)<br>CDK (9)<br>NO MOTHERS SHELTER (10)<br>NOT NECESSARY (11)<br>NOT CUSTOMARY (12)<br>OTHER (SPECIFY) (13):	
D33	Did you go to a health facility for a check of your health and/or your baby's health within 24 hours after delivery?	YES (1)<br>NO (0)<br>DON'T KNOW (96)	

MODULE E: SPENDING AND SAVINGS

INTERVIEWER: *"Now I'm going to ask you about the costs of your last pregnancy and delivery. Think back to the costs associated with your pregnancy and delivery and how you prepared for those costs."*

E1	***INTERVIEWER:*** *"Now we would like to talk to you about cash expenses that you had with your last delivery. Think about things that you had to buy in preparation for your delivery, for your journey to the health facility or home where you delivered, and at the time of your delivery at the health facility or home where you delivered. Are you ready to begin?"*

SURVEY ID

			AMOUNT (KWACHA)	NONE (0)	DON'T KNOW (96)
	About how much did you spend at that time on:				
	In preparation:				
	A	SUPPLIES (including clean delivery kit, gloves, syringes, plastic sheets, sterile blade, disinfectant, etc.)			
	B	BABY CLOTHES/BLANKET			
	On your journey:				
	C	TRANSPORTATION TO AND FROM (if own home, put 0)			
	D	MOTHERS' SHELTER/OTHER ACCOMODATION WHILE AWAITING DELIVERY			
	***At time of delivery*:**				
	E	PROVIDER/HEALTH CENTER FEES			
	F	INFORMAL PAYMENTS			
	G	TIPS			
	H	IN-KIND RESOURCES (estimate kwacha value)			
	I	DRUGS			
	J	DIAGNOSTIC TESTS			
	K	OTHER FEES			

NO.	QUESTION	POTENTIAL RESPONSES	SKIP
E2	Did you have money set aside in preparation for your last delivery?	YES (1)<br>NO (0)<br>DON'T KNOW (96)	If (0) or (96), skip to E8
E3	Do you think you saved enough money for your last delivery (in preparation, on your journey, at time of delivery)?	YES (1)<br>NO (0)<br>DON'T KNOW (96)	
E4	Where did you store the money that you saved?	AT YOUR HOME (1)<br>AT A FRIEND OR FAMILY MEMBER'S HOME (2)<br>IN A BANK ACCOUNT (3)<br>OTHER (SPECIFY) (4):	
E5	Did anyone else in the household *(e.g., your spouse)* have access to the money you saved?	YES (1)<br>NO (0)<br>DON'T KNOW (96)	
E6	About how far along in your pregnancy were you when you began saving this money?	WEEKS	

		MONTHS	
E7	Who else, if anyone, contributed to your delivery savings (in preparation, on your journey, at time of delivery)?<br><br>*Select all that apply.*	HUSBAND/PARTNER (1)<br>YOUR CHILDREN (2)<br>PARENT/GRANDPARENT (3)<br>OTHER FAMILY MEMBER (4)<br>FRIEND (5)<br>AUNTIE (6)<br>NO ONE (7)<br>OTHER (SPECIFY) (8):	
E8	In your opinion, how important is it to save money for delivery?	NOT IMPORTANT (1)<br>SLIGHTLY IMPORTANT (2)<br>MODERATELY IMPORTANT (3)<br>IMPORTANT (4)<br>VERY IMPORTANT (5)	
E9	Have you ever saved money in a bank account?	YES (1)<br>NO (0)<br>DON'T KNOW (96)	
E10	Have you ever transferred money on a mobile phone ("mobile money")?	YES (1)<br>NO (0)<br>DON'T KNOW (96)	In (0) or (96), skip to module F
E11	Who did you transfer "mobile money" to?	HUSBAND/PARTNER (1)<br>YOUR CHILDREN (2)<br>PARENT/GRANDPARENT (3)<br>OTHER FAMILY MEMBER (4)<br>FRIEND (5)<br>AUNTIE (6)<br>OTHER (SPECIFY) (7):	

MODULE F. POST-NATAL CARE

INTERVIEWER: ***"****Now I would like to ask you a few questions about any health care you and your baby received after your last delivery."*

NO.	QUESTION	POTENTIAL RESPONSES	SKIP
F1	Did you go to a health facility or health post for **ANY** postnatal checks **after the first 24 hours** following your last delivery?	YES (1)<br>NO (0)<br>DON'T KNOW (96)	If (0) or (96) skip to F6
F2	Did you go to a health facility or health post for a postnatal check approximately 3 days after your last delivery?	YES (1)<br>NO (0)<br>DON'T KNOW (96)	
F3	Did you go to a health facility or health post for a postnatal check between 7 and 14 days after your last delivery?	YES (1)<br>NO (0)<br>DON'T KNOW (96)	
F4	Did you go to a health facility or health post for a postnatal check	YES (1)<br>NO (0)<br>DON'T KNOW (96)	

	approximately 6 weeks after your last delivery?		

F5	***INTERVIEWER:*** *"Now I'm going to ask you about some common problems women face at health facilities while receiving postnatal care. As I mention each one, please tell me whether any of these were a problem for you during your postnatal care visits after your last delivery, and if so if they were a **major** or **minor** problem for you."*				
		**MAJOR PROBLEM (2)**	**MINOR PROBLEM (1)**	**NO PROBLEM (0)**	**UNDECIDED (96)**
A	TIME YOU WAITED TO SEE A PROVIDER	☐	☐	☐	☐
B	ABILITY TO DISCUSS PROBLEMS OR CONCERNS ABOUT YOUR PREGNANCY	☐	☐	☐	☐
C	AMOUNT OF EXPLANATION YOU RECEIVED ABOUT THE PROBLEM OR TREATMENT	☐	☐	☐	☐
D	PRIVACY FROM HAVING OTHERS SEE THE EXAMINATION	☐	☐	☐	☐
E	PRIVACY FROM HAVING OTHERS HEAR YOUR CONSULTATION DISCUSSION	☐	☐	☐	☐
F	THE CLEANLINESS OF THE FACILITY	☐	☐	☐	☐
G	HOW THE STAFF TREATED YOU	☐	☐	☐	☐
H	COST FOR SERVICES OR TREATMENTS	☐	☐	☐	☐

F6	Do you currently use something or try in any way to delay or avoid getting pregnant?	YES (1)<br>NO (0)<br>DON'T KNOW (96)	
F7	**INSTRUCTIONS:** Look back to question **B27** – Is the child from the most recent delivery still alive? *Confirm with respondent.*	YES (1)<br>NO (0)<br>DON'T KNOW (96)	If (0) or (96) skip to F15
F8	Are you currently breastfeeding your infant from your last delivery?	YES (1)<br>NO (0)<br>DON'T KNOW (96)	If (0) or (96) skip to F10
F9	Are you currently feeding the child foods other than breast milk and medicine?	YES (1)<br>NO (0)<br>DON'T KNOW (96)	
F10	In the last 2 weeks, did you seek healthcare for your child for any reason?	YES (1)<br>NO (0)<br>DON'T KNOW (96)	If (0) or (96), skip to F12
F11	Where did you first bring your child for health care?	HEALTH CARE CENTER (1)<br>HOSPITAL (2)<br>PHARMACY (3)<br>TRADITIONAL HEALER (4)<br>OTHER (SPECIFY) (5):	
F12	Has your child received any vaccinations?	YES (1)<br>NO (0)<br>DON'T KNOW (96)	If (0) or (96), skip to F15

		SURVEY ID

F13	**INSTRUCTIONS:** Based on **D1**, calculate child's age.<br><br>*Specify unit of response.*		
	**INSTRUCTIONS:** Ask to see the child's vaccination card. If available, use card to confirm the vaccines received and mark below. If card is unavailable, ask mother which vaccines the child has received. **BASED ON CALCULATED AGE FROM F13**, ask only about **AGE APPROPRIATE** vaccines.		
F14	Confirm you have the child's vaccine card in-hand.	YES (1)<br>NO (0)	

			CONFIRMED BY VACCINE CARD		CONFIRMED BY MOTHER	
			RECEIVED	NOT RECEIVED	RECEIVED	NOT RECEIVED
	**Did your child receive the following vaccines at birth?**					
	A	BCG	☐	☐	☐	☐
	B	Polio (OPV-0)	☐	☐	☐	☐
	**Did your child receive the following 6-week vaccines?**					
	C	Polio (OPV-1)	☐	☐	☐	☐
	D	DTP-HepB-Hib-1	☐	☐	☐	☐
	E	Pneumococcal (PCV)	☐	☐	☐	☐
	F	Rotavirus	☐	☐	☐	☐
	**Did your child receive the following 10-week vaccines?**					
	G	Polio (OPV-2)	☐	☐	☐	☐
	H	DTP-HepB-Hib-2	☐	☐	☐	☐
	I	Pneumococcal (PCV)	☐	☐	☐	☐
	J	Rotavirus	☐	☐	☐	☐
	**Did your child receive the following 14-week vaccines?**					
	K	Polio (OPV-3)	☐	☐	☐	☐
	L	DTP-HepB-Hib-3	☐	☐	☐	☐
	M	Pneumococcal (PCV)	☐	☐	☐	☐

INTERVIEWER: *"For the following question, please respond only if you feel comfortable doing so. Your response is optional."*			
F15	Were you tested for HIV during your last pregnancy?	YES (1)<br>NO (0)<br>PREFER NOT TO ANSWER (2)<br>DON'T KNOW (96)	
F16	What is your HIV status?	INFECTED (1)<br>NOT-INFECTED (2)<br>PREFER NOT TO ANSWER (3)<br>DON'T KNOW (96)	If (2), (3), or (96), skip to F23
F17	Did you take ARVs during your last pregnancy?	YES (1)<br>NO (0)<br>DON'T KNOW (96)	
F18	**INSTRUCTIONS:** Refer back to question **B28**. Did the respondent's baby survive beyond the day of birth?<br>*Confirm with respondent.*	YES (1)<br>NO (0)<br>DON'T KNOW (96)	If (0) or (96), skip to Module G

F19	Did your baby take ARVs for at least 6 weeks after birth?	YES (1)<br>YES, BUT BABY DIED BEFORE 6 WEEKS OF AGE (2)<br>NO (0)<br>DON'T KNOW (96)	
F20	Was your baby tested for HIV?	YES (1)<br>NO (0)<br>DON'T KNOW (96)	If (0) or (96), skip to F23
F21	How many weeks old was your baby when he/she was tested?<br><br>*Round to nearest full number.*		
F22	What was the result of your baby's HIV test?	INFECTED (1)<br>NOT INFECTED (2)<br>PREFER NOT TO ANSWER (3)<br>DON'T KNOW (96)	

F23 In the past three days, did you or a household member over 15 years of age engage in any of the following activities with your infant?

Select all that apply.

		**YES (1)**	**NO (0)**	**DON'T KNOW (96)**
A	READ BOOKS TO OR LOOK AT PICTURE BOOKS WITH INFANT	☐	☐	☐
B	TOLD STORIES TO INFANT	☐	☐	☐
C	SANG SONGS OR LULLABIES TO/WITH INFANT	☐	☐	☐
D	TOOK INFANT OUTSIDE HOME, COMPOUND, YARD, OR ENCLOSURE	☐	☐	☐
E	NAMES, COUNTED, OR DREW THINGS WITH INFANT	☐	☐	☐

F24 ***INTERVIEWER:*** *"For the following questions, please respond only if you feel comfortable doing so. Your response is optional. I am going to read you a list of problems. Please tell me how often each of these problems has happened to you in the PAST TWO WEEKS: never, once in a while, more than half the time, or almost always."*

		**NEVER (0)**	**ONCE IN A WHILE (1)**	**MORE THAN HALF THE TIME (2)**	**ALMOST ALWAYS (3)**
A	IN THE PAST 2 WEEKS, I HAVE BEEN SAD OR UNHAPPY	☐	☐	☐	☐
B	IN THE PAST 2 WEEKS, I HAVE NOT HAD ANY INTEREST IN THINGS (ACTIVITIES, WORK, PEOPLE)	☐	☐	☐	☐
C	IN THE PAST 2 WEEKS, I HAVE CRIED	☐	☐	☐	☐
D	IN THE PAST 2 WEEKS, I HAVE FELT LONELY OR SOCIALLY WITHDRAWN	☐	☐	☐	☐

F25	***INTERVIEWER:** "Now I am going to read you a list of things that you may have experienced. Please tell me how often each of these events have happened to you in the past two weeks: never, once in a while, a few times, or many times. Again, please respond only if you feel comfortable doing so. Your response is optional."*						
			**NEVER (0)**	**ONCE (1)**	**A FEW TIMES (2)**	**MANY TIMES (3)**	**N/A (4)**
	A	IN THE PAST 2 WEEKS, HOW MANY TIMES DID YOUR HUSBAND, BOYFRIEND, OR PARTNER PUSH OR SHOVE YOU OR SLAP YOU?	☐	☐	☐	☐	☐
	B	IN THE PAST 2 WEEKS, HOW MANY TIMES DID YOUR HUSBAND, BOYFRIEND, OR PARTNER KICK, DRAG, BEAT, CHOKE, OR BURN YOU?	☐	☐	☐	☐	☐

MODULE G. LAST PREGNANCY

***INTERVIEWER:** "Now I'm going to ask you some questions that are specific to your pregnancy leading up to your most recent delivery. Take a moment to think back to when you first knew you were pregnant and about your antenatal care. Are you ready to begin?"*

**NO.**	**QUESTION**	**POTENTIAL RESPONSES**			**SKIP**
**INTERVIEWER:** *"Ask to see if antenatal care card is available for the woman's last pregnancy that led to a delivery and confirm information provided by respondent."*					
G1	Did the woman provide you with her antenatal card?	YES (1)<br>NO (0)<br>DON'T KNOW (96)			
G2	How many times did you receive antenatal care at a health facility or health post during your last pregnancy?	NONE (0)<br>ONE TIME (1)<br>TWO TIMES (2)<br>THREE TIMES (3)<br>FOUR TIMES (4)<br>MORE THAN FOUR TIMES (5)			If (0) skip to End of Survey
G3	Did you discuss any of the following at an ANC visit:	**YES (1)**	**NO (0)**	**DON'T KNOW (96)**	
	A Where you would deliver the baby?	☐	☐	☐	
	B What you would do if a complication arose?	☐	☐	☐	
	C Saving money to cover costs of labor and delivery?	☐	☐	☐	
	D Your estimated delivery date?	☐	☐	☐	
G4	Do you remember what you were told your estimated delivery date was?	YES (1)<br>NO (0)<br>DON'T KNOW (96)			If (0), skip to G6
G5	What was your estimated delivery date? (DD MONTH YYYY)	___ ___ ___ ___ ___ ___ ___ ___<br>D D M M Y Y Y Y			

									SURVEY ID

	If EDD is on ANC card, copy it from card. If no card and date not known, enter 15th of the month.		
G6	About how many weeks or months pregnant were you at your **FIRST** ANC visit?	[ ][ ] WEEKS [ ][ ] MONTHS	

INTERVIEWER: *"Thank you for taking the time to take our survey. We have now reached the end of the survey."*

G7	Would you be willing to have someone come back and follow up on a few questions from the survey in the next couple of days?	YES (1)<br>NO (0)	

INTERVIEWER: *"Thank you very much. Do you have any additional comments you might like to add?"*

COMMENTS:

END OF SURVEY

INSTRUMENT REVIEW

Enumerator Initials:	
Date (DD/MM/YYYY)	
Supervisor Initials:	
Date (DD/MM/YYYY)	

Data Entry Initials:	
Date (DD/MM/YYYY)	
Supervisor Initials:	
Date (DD/MM/YYYY)	
