# Supplementary material for: Characteristics of maternity waiting homes and the women who use them: Findings from a baseline cross-sectional household survey among SMGL-supported districts in Zambia
Source: PLoS One. 2018 Dec 31;13(12):e0209815. doi: 10.1371/journal.pone.0209815 (PMC6312364; doi:10.1371/journal.pone.0209815)
Supplement: S2 File — Household Survey Baseline Impact Evaluation–Tonga. (PDF) [file pone.0209815.s002.pdf]

SURVEY ID

Instrument ID:

The MAHMAZ Project

Baseline Impact Evaluation – Household Survey **TONGA**

Target Audience:

Women who have delivered a child in the last 12 months, who are ≥ 15 years of age, and who live within the study facility catchment areas

KUSEBA KUFWAAFWI

SS1	Mbongaye bamakaintu balaa myaka kkumi ayosanwe (15) kusikila ku makumi one afuka (49) bawvula kukkala mumukwashi, kusanganya abakafwa abakazwa mu myezi ili 12 eyi yainda?<br><br>*Write down the number of women.*		If none, thank person and move to next household.
SS2	Sena ali bamakaintu baambwa waawa kuli bakatumbuka mu myezi kkumi ayibili yainda (mwaka omwe), kutalanganya nseba yamwana na banyina nobakamana kutumbuka	Yes (1)<br>No (0)<br>Don't know (96)	If (0) or (96), thank person and move to next household
SS3	Kuti naa mukaintu ooyu mumukwashi tako, hena inga mwacikonzya kuvwiila mibuzyo injatikizya makani ada atoomwe akutumbuka kwakwe?	Yes (1)<br>No (0)<br>Don't know (96)	
**INTERVIEWER: IF YOU HAVE ANSWERED YES TO SS1 AND YES TO SS2, THEN PROCEED WITH THE INFORMED CONSENTING PROCESS. PLACE THE UNIQUE ID STICKER ON THE INSTRUMENT AND ON THE HOUSEHOLD CONSENT FORM A.**			
	Confirm consent was granted<br><br>*Draw a check mark if consent was granted.*		
**IF CONSENT WAS GRANTED, PLACE A SECOND UNIQUE ID STICKER ON THE PAPER VERSION OF THE INSTRUMENT.**			

SURVEY ID | | | | | | |

MODULE A. LOCATION

INSTRUCTIONS: Complete before administering the rest of the survey

NO.	FIELD	CODE	RESPONSE
A1	Province	EASTERN (1)<br>SOUTHERN (2)<br>LUAPULA (3)	
A2	District	CHOMA (1)<br>KALOMO (2)<br>NYIMBA (3)<br>PEMBA (4)<br>LUNDAZI (5)<br>MANSA (6)<br>CHEMBE (7)	
A3	Health Facility Catchment Area	CHOMA DISTRICT<br>CHOMA GENERAL (801001)<br>MANGUNZA (801019)<br>MACHA MISSION (801002)<br>MASUKU MISSION (801021)<br>MBABALA (801022)<br>MOCHIPAPA (801023)<br>SIMAKUTU (801043)<br>KALOMO DISTRICT<br>CHIFUSA HC (804023)<br>CHILALA HC (804024)<br>DIMBWE HC (804019)<br>HABULILE HC (804032)<br>KALOMO DISTRICT HOSPITAL (804002)<br>KANCHELE HC (804014)<br>MAWAYA HC (804034)<br>MOONDE HP (804042)<br>MUKWELA HC (804020)<br>SIACHITEMA HC (804013)<br>PEMBA DISTRICT<br>JEMBO (801413)<br>MUZOKA (801419)<br>NYIMBA DISTRICT<br>CHIPEMBE RHC (307010)<br>HOFMEYR ZONAL HC (307011)<br>KACHOLOLA RHC (307012)<br>MKOPEKA RHC (307016)<br>NYIMBA DISTRICT HOSPITAL (307001)<br>MANSA DISTRICT<br>FIMPULU (403017)<br>KABUNDA (403018)<br>LUBENDE (403041)<br>MANO (403026)<br>MANSA GENERAL HOSPITAL (403001)<br>MIBENGE (403029)<br>MUSAILA (403030)<br>MUTITI (403031)<br>MUWANGUNI (403032)	

		CHEMBE DISTRICT<br>KUNDAMFUMU (403023)<br>LUKOLA (403037)<br>LUNDAZI DISTRICT<br>CHIKOMENI (405026)<br>KAMSARO (305034)<br>KAPICHILA (305023)<br>LUKWISIZI (305040)<br>LUNDAZI HOSPITAL (305032)<br>LUSUNTHA (305021)<br>MWASE LUNDAZI ZONAL (305011)<br>NKHANGA (305046)<br>NYANGWE (305020)<br>PHIKAMALAZA (305031)<br>ZUMWANDA (305024)	
A4	Village Name<br><br>*Write in the name of the village*		
**GPS COORDINATES, BWEZA YOMWE**			
A5	Latitude		
A6	Longitude		
**GPS COORDINATES, BWEZA ZYOBILO**			
A7	Latitude (decimal format)		
A8	Longitude (decimal format)		
A9	Date of Interview (DD/MM/YYYY)		
A10	Start time of interview<br>(24:00 format)		

SURVEY ID

MODULE B. HOUSEHOLD ENUMERATION

INSTRUCTIONS: Confirm that the person who you are speaking with is the head of the household or the head woman of the household.

INTERVIEWER: *"I am now going to ask you some basic information about you and the members of your household. For the purposes of the following questions, let us define a household as a group of related or unrelated people who usually live together on a premise, acknowledge the same person as the head of the household, and who have a common cooking and eating arrangement."*

NO.	QUESTION	POTENTIAL RESPONSES	SKIP
B1	Ino mwakali amyaka yongaye kusekelela kwakuzyalwa kwenu kwa ciindi cakamana?<br><br>*Unit of response in years.*		
B2	Hena kuli nomwakanjide cikolo kuya kwiiya?	YES (1)<br>NO (0)<br>DON'T KNOW (96)	If (0) or (96), skip to B4
B3	Ino mwakagolela mubbuku naa giledi nzi?<br><br>*Write grade level (ie: 03 for grade 3). If <1 year completed, write down 00. If >12 years completed, write down 13.*	DON'T KNOW (96)	
B4	Ino mulibachikombelo/lukondo nzi?	CATHOLIC (1)<br>PROTESTANT (2)<br>MUSLIM (3)<br>OTHER (SPECIFY) (4)	
B5	Muzulilwa kubasyobo nzi?		
B6	Kukwata na kukwatwa?	MARRIED/COHABITING (1)<br>DIVORCED (2)<br>SEPARATED (3)<br>WIDOWED (4)<br>NEVER-MARRIED (5)	If (2), (3), (4), or (5), skip to B8
B7	*If respondent is the male head of household:*<br>Ino mujisi bamakaintu bongaye kubasanganya boonse mbomukwete ambomutakwete/bakumbali?<br><br>*If respondent is NOT male head of household:*<br>Ino mwaalumi mutwe wamukwasyi ujisi bamakaintu bongaye kubasanganya mbakwete ambatakwete/bakumbali?		

SURVEY ID

B8	Balibongaye basankwa abasimbi batana sika myaka yakuzyalwa yosanwe (5) bavwula kukkala mumukwasyi wenu?<br><br>*Include children who are in boarding school at the moment. If none, write down 00.*	BOYS<br><br>GIRLS	
B9	Balibongaye basankwa abasimbi balaa myaka yakuzyalwa iitalikila ali yosanwe (5) kusika amyaka kkumi ayone (14) bavwula kukkala mumukwasyi wenu?<br><br>*Include children who are in boarding school at the moment. If none, write down 00.*	BOYS<br><br>GIRLS	
B10	Balibongaye bamaalumi abamakaintu balaa myaka yakuzyalwa iitalikila kkumi ayosanwe (15) kusika myaka makkumi one alusele (49) bavwula kukkala mumukwasyi wenu?	MEN<br><br>WOMEN	
	Kusanganya andinywe balibongaye bamaalumi abamakaintu balaa myaka yakuzyalwa iitalikila kkumi ayosanwe (15) kusika myaka makkumi one afuka (49) bakali kukkala mumukwasyi wenu bakafwa mu myezi ili twelufu yayinda?	MEN<br><br>WOMEN	
B11	Kusanganya andinywe balibongaye bamaalumi abamakaintu balaa myaka yakuzyalwa iitalikila makkumi osanwe (50) kusika makkumi cisambomwe ayone (64) bavwula kukkala mumukwasyi wenu?	MEN<br><br>WOMEN	
B12	Kusanganya andinywe balibongaye bamaalumi abamakaintu balaa myaka yakuzyalwa makkumi cisambomwe ayosanwe (65) naanka bapati kwiinda waawa bavwula kukkala mumukwasyi wenu?	MEN<br><br>WOMEN	
**INSTRUCTIONS:** Count and record the total number (B8 to B12) of household members.			
B13	Zwidilizya amwiinguzyi: Tede bali (mweelwe) bantu mumukwasyi wenu antoomwe?	YES (1)<br>NO (0)	

SURVEY ID | | | | | | |

INSTRUCTIONS: Ask the respondent to list the names of all women aged 15-49 in the household, including those who passed away in the last 12 months (1 year). Emphasize that you are also looking for information on individuals who have passed away in the last 12 months (1 year). Fill out column A with all names provided, and then continue to answer B-F for each person before selecting a respondent.
INTERVIWER: Lino inga ndayanda kuti mundaambile mazyina abamakzintu boonse bavwula kukkala mumukwasyi balo balaa myaka iili kkumi ayosanwe (15) kusika balaa myaka makkumi one alusele (49). Twalomba musanganye amazyina abamakaintu bakafwa mu myezi kkumi ayibili yainda (mwaka omwe).

TABLE 1. ROSTER OF WOMEN AGED 15-49 YEARS

	A. Ndalomba mundaambile mazyina aakutaanguna and age abamakaintu balaa myaka kkumi ayosanwe (15) kusika makkumi one alusele (49) bavwula kukkala mumukwasyi ooyu for at least 4 days a week. Musanganye abakafwa mumyezi kkumi ayibili yainda (mwaka omwe)<br><br>Ensure the number includes those who would have been living there if they didn't pass away/move away in the past 12 months.<br>*The number of women in this list should be greater or equal to the number of women in **B10**.*	B. Mumyezi kkumi ayibili yainda (mwaka omwe), sena (zyina) wakaba ada lyakasisya nsondo zyili makkumi otatwe azyosanwe (35)?<br><br>*That is, was (name) pregnant at least up until ~3 weeks before her estimated delivery date?*<br><br>*Instructions: Note that this includes a delivery, still birth, neonatal death, etc.at any point within the past 12 months*<br><br>YES (1)<br>NO (0)<br>DON'T KNOW (96)<br><br>*If (0) or (96), skip to next person.*	C. Sena (*name) ucipona?<br><br>YES (1)<br>NO (0)<br><br>*If (1), skip to E.*<br><br>**Check list and input name*	D Sena mula or somebody else lyaaba kuingula mibuzyo misyoonto biyo abusena bwa (zyina) iijatikizya da lyakwe?<br><br>YES (1)<br>NO/ DON'T KNOW (0)<br><br>*If (0), skip to next person.*	E. Is (name) potentially eligible to take the survey?<br><br>*If **B=1** and (**IF APPLICABLE) D=1**, mark the box below.*<br><br>**Check list and input name*	**AFTER ALL WOMEN HAVE BEEN LISTED, TO SELECT A RESPONDENT:**<br><br>1. Roll the die<br>2. From the 1st checked box in Column E, count up to the rolled number, beginning again at the 1st checked box if needed until number is reached<br>3. Roll the die again<br>4. From the checked box you landed on after the 1st roll, count up to the 2nd rolled number, beginning again at the 1st checked box if needed until the 2nd number is reached<br>5. Select this woman<br>6. If woman selected is ALIVE, proceed to **Question B24**<br>7. If woman selected is DECEASED, proceed to Proxy Household Survey
B14					☐	
B15					☐	
B16					☐	
B17					☐	
B18					☐	
B19					☐	

SURVEY ID | | | | | | |

B20					☐	
B21					☐	
B22					☐	
B23					☐	

SURVEY ID | | | | | | |

NO.	QUESTION	POTENTIAL RESPONSES	SKIP
B23A	Sena uli amyaka yongaye(*name)<br><br>*Input name	[ ][ ]	
B24	Sena (name) nkwali kutegwa atole lubazu mukwiingula mibuzyo?	YES (1)<br>NO (0)<br>DON'T KNOW (96)	If (1), skip to consent then proceed to B27
B25	Sena tupangane ciindi cakuboola alimwi anooliko/anoolibambilide?	YES (1)<br>NO (0)<br>DON'T KNOW (96)	If (0) or (96), resample from potentially eligible women in TABLE 1
B26	Were you able to reschedule another time?	YES (1)<br>NO (0)	If (0), resample from potentially eligible women in TABLE 1
If you are unable to reschedule a time to come back and survey the sampled woman, go back to TABLE 1 and resample another potentially eligible woman. If you are re-visiting the household a subsequent time and the woman is now available, **proceed from Question B27**.			

INSTRUCTIONS: Make sure to obtain consent or assent (if the sampled woman is 15, 16 or 17 years old – refer to B23A), including a signature, from the sampled woman. If the woman is not able to sign, please have the woman provide a thumbprint. These questions will determine whether or not the sampled woman is eligible to proceed to the full household survey. If she is ineligible, then re-sample from Roster Table 1. If there are no more potentially eligible women to sample from, thank the woman and move on to the next household. If she is eligible, proceed to Module C.

STOP: MAKE SURE CONSENT OR ASSENT WAS OBTAINED FROM (NAME). PLACE A THIRD UNIQUE ID STICKER ON THE CONSENT FORM B – FOR THE ELIGBLE WOMAN. IF SHE HAS ALREADY CONSENTED AS THE HOUSEHOLD HEAD, NO NEED TO ISSUE A CONSENT FORM B.

SIKUBUZYA: twalumba kuti mwazumina kutola lubazu mukuingula mibuzyo yesu. Ino ndilamubuzya mibuzyo misyoonto biya kujatikizya makani akutumbuka kwenu kwaino-ino

NO.	MUBUZYO	BWIINGUZI BULANGILWA	SOTOKA
B27	Sena mwana wenu ucipona?	YES (1)<br>NO (0)<br>DON'T KNOW (96)	If (1), continue to Module C<br>If (96), skip to B29
B28	Ino mwana wenu wakafwa lili?	BEFORE OR ON DAY OF DELIVERY (1)<br>WITHIN ONE MONTH AFTER DELIVERY (2)<br>OTHER (3)	If (2) or (3), continue to Module C
B29	Sena mwana wenu mwaamutumbuka ciindi ncaakali kulangilwa kuzyalwa kacitaninga sika?	YES (1)<br>NO (0)<br>DON'T KNOW (96)	If (0), continue to Module C<br>If (96), end and re-sample from Roster Table 1
B30	Ino nomwakamutumbuka mwana wenu, kwakaceede nsondo zyongaye kuti cisike ciindi ncomwakali kulangilwa kutumbuka?	<= 3 WEEKS (1)<br>>3 WEEKS (2)<br>DON'T KNOW (96)	If (1), continue to Module C<br>If (2), end and re-sample from Roster Table 1<br>If (96), end and re-sample from Roster Table 1

SURVEY ID | | | | | | |

MODULE C. DEMOGRAPHICS

INSTRUCTIONS: After eligible respondent has been randomly sampled from all eligible respondents, proceed with the instrument. Ensure that the woman selected to proceed with the survey has delivered a child **within the last year**. This section is to get basic demographics on the household and the respondent.
INTERVIEWER: *Ino ndilamubuzya mibuzyo iijatikizya ndinwe amukwasyi wenu.*

NO.	QUESTION	POTENTIAL RESPONSES	SKIP
C1	Sena ndinywe mutwe wamukwasyi?	YES (1)<br>NO (0)	If (1), skip to C9
C2	Ino mucitene buti amutwe wang'anda?	SPOUSE (1)<br>CHILD (2)<br>GRANDCHILD (3)<br>NIECE (4)<br>AUNTIE/OTHER RELATIVE (5)<br>OTHER (SPECIFY) (6):	
C3	Hena kuli nomwajide cikolo kuya kwiiya??	YES (1)<br>NO (0)<br>DON'T KNOW (96)	If (0) or (96), skip to C5
C4	Ino mwakagolela mubbuku naa giledi nzi?<br><br>*If <1 year completed, write down 00. If >12 years completed, write down 13.*	[ ][ ]<br><br>DON'T KNOW (96)	
C5	Ino mulibachikombelo/lukondo nzi?	CATHOLIC (1)<br>PROTESTANT (2)<br>MUSLIM (3)<br>OTHER (SPECIFY) (4):	
C6	Muzulilwa kubasyobo nzi?		
C7	Kukwata na kukwatwa?	MARRIED/COHABITING (1)<br>DIVORCED (2)<br>SEPARATED (3)<br>WIDOWED (4)<br>NEVER-MARRIED (5)	If (2), (3), (4) or (5), skip to C9
C8	Ino balumi benu bajisi bamakaintu bongaye kusanganya baang'anda atoomwe abakumbali?<br><br>*If don't know, write down 96.*	[ ][ ]	
C9	Ino zyiindi zyongaye nzi mwakaba aada?		
C10	Ino bali bongaye bana bapona mbomwakaba aabo?		

INTERVIEWER: *"Ino tubandike makani aang'anda yenu."*

SURVEY ID | | | | | | |

NO.	QUESTION	POTENTIAL RESPONSES	SKIP
C11	Nkuli nkomujana kapati meenda aakunywa aabantu bamumukwasyi?	*PIPED WATER*<br>PIPED INTO DWELLING (1)<br>PIPED TO YARD/PLOT (2)<br>PUBLIC TAP/STANDPIPE (3)<br>TUBE WELL OR BOREHOLE (4)<br>*DUG WELL*<br>PROTECTED WELL (5)<br>UNPROTETED WELL (6)<br>*WATER FROM SPRING*<br>PROTECTED SPRING (7)<br>UNPROTECTED SPRING (8)<br>RAINWATER (9)<br>TANKER TRUCK (10)<br>CART WITH SMALL TANK (11)<br>SURFACE WATER (12)<br>(RIVER/DAM/LAKE/POND/STREAM/CANAL/<br>IRRIGATION CHANNEL)<br>BOTTLED WATER (13)<br>OTHER (PLEASE SPECIFY) (14):	If (13), skip to C14
C12	Ino busena oobu kuli nkobubede?	IN OWN DWELLING (1)<br>IN OWN YARD/PLOT (2)<br>ELSEWHERE (3)	If (1) or (2), skip to C14
C13	Citola ciindi cilamfu buti kuyoo teka meenda akujoka?<br><br>*If minutes, write down how many minutes.*	MINUTES (1): [ ][ ]<br><br>DON'T KNOW (96)	
C14	Sena kuli ncomucita kumeenda kutegwa ayelele kunyugwa?	YES (1)<br>NO (0)<br>DON'T KNOW (96)	
C15	Ino ncimbuzi camusyobo nzi amumukwasyi wenu ncomuvula kubelesya?	*FLUSH OR POUR FLUSH TOILET*<br>FLUSH TO PIPED SEWER SYSTEM (1)<br>FLUSH TO SEPTIC TANK (2)<br>FLUSH TO PIT LATRINE (3)<br>FLUSH TO SOMEWHERE ELSE (4)<br>FLUSH, DON'T KNOW WHERE (5)<br>*PIT LATRINE*<br>VENTILATED IMPROVED PIT LATRINE (6)<br>PIT LATRINE WITH SLAB (7)<br>PIT LATRINE WITHOUT SLAB/OPEN PIT (8)<br>COMPOSTING TOILET (9)<br>BUCKET TOILET (10)<br>HANGING TOILET/HANGING LATRINE (11)<br>NO FACILITY/BUSH/FIELD (12)<br>OTHER (SPECIFY) (13):	
C16	Sena kuli mikwasyi iimbi njomubelesya limwi cimbuzi eeci?	YES (1)<br>NO (0)	

C17	Sena mukwasyi wenu ulijisi zyiccilila *(item must be functioning usually):*		YES (1)	NO (0)	DON'T KNOW (96)
	A	MALAITI	☐	☐	☐
	B	MALAITI ASOLA	☐	☐	☐
	C	GENERATA	☐	☐	☐

D	LAMBE	☐	☐	☐
E	FILINJI	☐	☐	☐
F	CHIBULO CAMAGESI CAKUKASAAZYISYA CAKULYA	☐	☐	☐
G	CITOFU CA MALASHA	☐	☐	☐
H	CITOFU CANKUNI	☐	☐	☐
I	CITOFU CA MALAITI	☐	☐	☐
J	BULO	☐	☐	☐
K	MATILESI	☐	☐	☐
L	CUUNO	☐	☐	☐
M	TAFULE	☐	☐	☐
N	CABATI	☐	☐	☐
O	MASEETI	☐	☐	☐
P	NKOLOKO	☐	☐	☐
Q	CIVUPAULA MUWO UTONTOLA	☐	☐	☐
R	MUCHINI WAKUSUMYA ZISANI	☐	☐	☐
S	KASABWI KAMANSENYA	☐	☐	☐
T	INTERNET	☐	☐	☐
U	NKOLOKO YAAKWANZA	☐	☐	☐
V	AAKUYOBWEDA MALI KU BBANGA	☐	☐	☐
W	PULAU	☐	☐	☐
X	BBALA	☐	☐	☐
Y	KANCINI KAKU MAANZA	☐	☐	☐
Z	TALAKITA	☐	☐	☐
AA	CIGAYO	☐	☐	☐
BB	FOSHOLO	☐	☐	☐
CC	CCEBA	☐	☐	☐
DD	PIKI	☐	☐	☐
EE	NJINI WAMAANZI	☐	☐	☐
FF	MPULAZI YAKULIMA	☐	☐	☐
GG	ZISAMU ZIZYALA MICHELO	☐	☐	☐
HH	WAILESI	☐	☐	☐
II	CIPEKUPEKU	☐	☐	☐

JJ	LUWAILE LWAKU MAANZA	☐	☐	☐
KK	LUWAILE LUTALI LWA KUMAANZA	☐	☐	☐
LL	COMPUTER	☐	☐	☐
MM	CILIMBA CAMA TEEPU	☐	☐	☐
NN	MINCCINI YAKWEEBELEZYESYA MA FILIMU KUCIPEKUKUPEKU	☐	☐	☐
OO	NCINGA	☐	☐	☐
PP	HONDA/MUDUDUUDU	☐	☐	☐
QQ	CIKOCHI CIKWELWA ABANYAMA	☐	☐	☐
RR	MOOTA /LOLI	☐	☐	☐
SS	BWATO BULAA NJINI	☐	☐	☐
TT	KATO KANINI	☐	☐	☐

C18	Ino mumukwasyi wenu babelesya mulilo wamusyobo nzi kujika kanji kanji?	ELECTRICITY (1)<br>SOLAR POWER (2)<br>LIQUID PROPANE GAS (LPG) (3)<br>NATURAL GAS (4)<br>BIOGAS (5)<br>KEROSENE (6)<br>COAL, LIGNITE (7)<br>CHARCOAL (8)<br>WOOD (9)<br>STRAW/SHRUBS/GRASS (10)<br>AGRICULTURAL CROP (11)<br>ANIMAL DUNG (12)<br>NO FOOD COOKED IN HOUSEHOLD (13)<br>OTHER (SPECIFY) (14):	If (13), skip to C20
C19	Ino muvula kujikila kuli aba mukwasyi wenu?	IN THE HOUSE (1)<br>IN A SEPARATE BUILDING (2)<br>OUTDOORS (3)<br>OTHER (SPECIFY) (4):	
C20	Ino cibuye cang'anda mpati cishingululidwe aanzi?<br><br>*OBSERVE THE FLOOR TO CONFIRM. (If more than one material, select the one that is "most" common)*	<u>NATURAL FLOOR</u><br>EARTH/SAND (1)<br>DUNG (2)<br><u>RUDIMENTARY FLOOR</u><br>WOOD PLANKS (3)<br>PALM/BAMBOO/REEDS (4)<br><u>FINISHED FLOOR</u><br>PARQUET/POLISHED WOOD (5)<br>VINYL (PVC) OR ASPHALT STRIPS (6)<br>CERAMIC/TERRAZZO TILES (7)<br>CONCRETE CEMENT (8)<br>CARPET (9)<br>OTHER (SPECIFY) (10):	

C21	Ino ciluli cang'anda yenu cimvumbindwe nzi?<br><br>*OBSERVE THE ROOF TO CONFIRM. (If more than one material, select the one that is "most" common)*	*NATURAL ROOFING*<br>NO ROOF (0)<br>THATCH/PALM LEAF (1)<br>*RUDIMENTARY ROOFING*<br>RUSTIC MAT (2)<br>PALM/BAMBOO (3)<br>WOOD PLANKS (4)<br>CARDBOARD (5)<br>*FINISHED ROOFING*<br>METAL/IRON SHEETS (6)<br>WOOD (7)<br>CALAMINE/CEMENT FIBRE (ASBESTOS) (8)<br>CERAMIC/HARVEY TILES (9)<br>CEMENT (10)<br>ROOFING SHINGLES (11)<br>MUD TILES (12)<br>OTHER (SPECIFY) (13):	
C22	Ino bwaanda bwa nga'anda yenu nkobuli aanze buyakidwe aanzi?<br><br>*OBSERVE THE WALLS TO CONFIRM. (If more than one material, select the one that is "most" common)*	*NATURAL WALLS*<br>NO WALLS (0)<br>CANE/PALM/TRUNKS (1)<br>MUD (2)<br>*RUDIMENTARY WALLS*<br>BAMBOO/POLE WITH MUD (3)<br>STONE WITH MUD (4)<br>PLYWOOD (5)<br>CARDBOARD (6)<br>REUSED WOOD (7)<br>*FINISHED WALLS*<br>CEMENT (8)<br>STONE WITH LIME/CEMENT (9)<br>BRICK (10)<br>CEMENT BLOCKS (11)<br>WOOD PLANKS (12)<br>OTHER (SPECIFY) (13):	
C23	Sena kuli umwi wamumukwasyi wenu uujisi nyika yakulima?	YES (1)<br>NO (0)<br>DON'T KNOW (96)	If (0) or (96), skip to C25
C24	Ino mukwasyi wenu ujisi nyika mpati buti yakulima?	☐☐ ☐☐☐☐<br>LIMA (1) QUANTITY<br>ACRES (2)<br>HECTARES (3)<br>SQUARE METERS (4)<br>DON'T KNOW (96)	

C25	Ino bali bongaye abanywama aaba mukwasyi wenu mbomujisi?		**NUMBER**	**NONE (00)**	**DON'T KNOW (96)**
	A	NG'OMBE ZYA CITONGA	☐☐☐☐	☐	☐
	B	NG'OMBE ZYA MUKUPA	☐☐☐☐	☐	☐
	C	NG'OMBE ZYACISYU	☐☐☐☐	☐	☐

D	MAHACI/MADINKI/ AMBONGOLO		☐	☐
E	MPONGO		☐	☐
F	MBELELE		☐	☐
G	NGULUBE		☐	☐
H	NKUKU/ABAYUNI BAMBI		☐	☐
I	BASULWE		☐	☐
J	BANYAMA BAMBI		☐	☐

NO.	QUESTION	POTENTIAL RESPONSES	SKIP
C26	Twalomba amutodezye nzila zyoonse zyivwula kubelesyegwa kuleta mali mumukwasyi.<br><br>*Select all that apply.*	SALARIED EMPLOYMENT (1)<br>SMALL BUSINESS, SHOP OR KIOSK (2)<br>SMALL HOUSEHOLD INCOME GENERATING ACTIVITY (3)<br>DOWRY (4)<br>SALE OF CROPS/ANIMALS (5)<br>SALE OF ASSETS (6)<br>REMITTANCES (CASH DONATIONS FROM FRIENDS/FAMILY) (7)<br>GOVERNMENT/NGO AID, GRANT OR OTHER FINANCIAL SUPPORT (8)<br>CASUAL DAILY WORK (9)<br>OTHER (SPECIFY) (10):	
C27	Naa kuti bamumukwasyi wenu bayanda kuti kakumbile mali kubbanga nokuba kubakolotesya mali (kutabikilizya bacilongwe naa kuba mukowa), sena mukwasyi wenu inga wakozya kukumbila mali?	NO (0)<br>PROBABLY NOT (1)<br>PROBABLY YES (2)<br>DEFINITELY YES (3)<br>DON'T KNOW (96)	
C28	Sena boonse bamakaintu balaa mada kusanganya abana balaa myaka iitasiki ali yosanwe bakoona mukasabwi kamansenya masiku aayinda?	YES (1)<br>NO (0)<br>DON'T KNOW (96)	
C29	Sena bamumukasyi wenu kalakozya kubbaddelela bana mali makucikolo antoomwe aziyandika kucikolo?	YES (1)<br>USUALLY (2)<br>SOMETIMES (3)<br>RARELY (4)<br>OTHER (SPECIFY) (5):<br>NO (0)	
C30	Mwezi wainda sena kuli umwi wamumukwasyi wenu wakala buzuba boonse amasiku kukunyina kulya?	YES (1)<br>NO (0)<br>DON'T KNOW (96)	

C31	Sena kuli bamwi akati kabana benu kukati kang'anda yenu bakalala anzala masiku ainda?	YES (1)<br>NO (0)<br>DON'T KNOW (96)	
C32	Sena munzi wenu inga wayindwa kuguwo naa mvula mpati kakunyina akusinyikilwa kupati?	YES (1)<br>NO (0)<br>DON'T KNOW (96)	
C33	Ciindi mvula yawa, sena meenda meenda nasweka mubusena bumwi bwang'anda bana mobalala?	YES (1)<br>NO (0)<br>DON'T KNOW (96)	

MODULE D. LAST DELIVERY/MOTHERS' SHELTER

INTERVIEWER: Ino ndilamubuzya mibuzyo iigaminina kutumbuka kwenu kwaino-ino. Kutaanguna amuyeeye ciindi cakutumbuka kwaino-ino kacitana sika antoomwe akutumbuka kwini. Mpoona tulababandika zyipanga hondi zyabamatumbu. Sena mwalibambila kutalika?

NO.	QUESTION	POTENTIAL RESPONSES	SKIP
D1	Ino kutumbuka kwenu kwaino-ino kwakali lili?<br>(DD MONTH YYYY)<br><br>*If date not know, ask for under 5 card. If no under 5 card, write 15th*	___ ___ ___ ___ ___ ___ ___ ___<br>D D M M Y Y Y Y	
D2	Kuze aaino awa, sena kuli nomwakanvwide zyipanga hodi zya bamatumbu?	YES (1)<br>NO (0)<br>DON'T KNOW (96)	If (0) or (96), skip to D18
D3	Kuzwa kuli /nkulini nkumwakavwida zyamaanda abamatumbu?<br><br>*(Select all that apply)*	CHIEF (1)<br>HEADMEN (2)<br>HEALTH CARE WORKER (3)<br>SMAG (4)<br>TRADITIONAL BIRTH ATTENDANT (5)<br>FAMILY MEMBER (6)<br>ANOTHER MOTHER (7)<br>OTHER COMMUNITY MEMBER (8)<br>RADIO (9)<br>OTHER (SPECIFY) (10):	
D4	Kulanganya makani akutumbuka ngotwaambaula waawa, sena kuli nomwaka kkede kuzyipanga hondi zyabamatumbu kamutaninga tumbuka naanka kamutumbukide kukkalilila kaambo kali koonse?	YES (1)<br>NO (0)	If (1), skip to D5

D4a	Naataku. kaambo nzi?<br><br>*Select all that apply.*	NO MOTHERS SHELTER (1)<br>NO PERMISSION FROM HUSBAND OR FAMILY (2)<br>NO MONEY (3)<br>POOR QUALITY (4)<br>NOT CLEAN (5)<br>TOO CROWDED (6)<br>NOT CULTURALLY APPROPRIATE (7)<br>NOT SAFE (8)<br>DELAYS DELIVERY (10)<br>DIDN'T KNOW ABOUT MOTHERS SHELTER (11)<br>OTHER (12)<br>IF OTHER, SPECIFY	Skip to D18

INSTRUCTIONS: Ask the respondent for what reason(s) did she stay at a mothers' shelter, and then prompt her with the reasons listed below.

D5		Akaambo kamakani aaccilila, ino mukweezyeezya biyo mwakakkala masiku ongaye kucipanga hondi cabamatumbu?	**NUMBER OF NIGHTS**	**NONE (0)**	**DON'T KNOW (96)**
	A	kupimwa da lwakusaanguna		☐	☐
	B	kupimwa da cindi cimbi biyo		☐	☐
	C	Kulindilila kutumbuka		☐	☐
	D	nomwakamana Kulekezyegwa kucibbadela/nokwaamana biyo kutumbuka		☐	☐
	E	Kujoka kucibbadela kwainda mazuba otatwe aakutumbuka		☐	☐
	F	kupimwa kucibbadela kwainda mazuba aali ciloba kusikila aali kkumi aone aakutumbuka		☐	☐
	G	kupimwa kucibbadela kwainda nsondo zili cisambomwe zyakutumbuka		☐	☐

	H	Azimbi biyo (Zyaambe)		☐	☐

NO.	QUESTION	POTENTIAL RESPONSES	SKIP
D6	Nkukuli kuchipanga hondi cabamatumbu nkomwakakkala kwaciindi cilanfwu?<br><br>*Confirm the longest number of nights the respondent stayed at a mothers' shelter.*	CHOMA DISTRICT<br>CHOMA GENERAL (801001)<br>MANGUNZA (801019)<br>MACHA MISSION (801002)<br>MASUKU MISSION (801021)<br>MBABALA (801022)<br>MOCHIPAPA (801023)<br>SIMAKUTU (801043)<br>KALOMO DISTRICT<br>CHIFUSA HC (804023)<br>CHILALA HC (804024)<br>DIMBWE HC (804019)<br>HABULILE HC (804032)<br>KALOMO DISTRICT HOSPITAL (804002)<br>KANCHELE HC (804014)<br>MAWAYA HC (804034)<br>MOONDE HP (804042)<br>MUKWELA HC (804020)<br>SIACHITEMA HC (804013)<br>PEMBA DISTRICT<br>JEMBO (801413)<br>MUZOKA (801419)<br>NYIMBA DISTRICT<br>CHIPEMBE RHC (307010)<br>HOFMEYR ZONAL HC (307011)<br>KACHOLOLA RHC (307012)<br>MKOPEKA RHC (307016)<br>NYIMBA DISTRICT HOSPITAL (307001)<br>MANSA DISTRICT<br>FIMPULU (403017)<br>KABUNDA (403018)<br>LUBENDE (403041)<br>MANO (403026)<br>MANSA GENERAL HOSPITAL (403001)<br>MIBENGE (403029)<br>MUSAILA (403030)<br>MUTITI (403031)<br>MUWANGUNI (403032)<br>CHEMBE DISTRICT<br>KUNDAMFUMU (403023)<br>LUKOLA (403037)<br>LUNDAZI DISTRICT<br>CHIKOMENI (405026)<br>KAMSARO (305034)<br>KAPICHILA (305023)<br>LUKWISIZI (305040)<br>LUNDAZI HOSPITAL (305032)<br>LUSUNTHA (305021)<br>MWASE LUNDAZI ZONAL (305011)<br>NKHANGA (305046)<br>NYANGWE (305020)<br>PHIKAMALAZA (305031)	

		ZUMWANDA (305024)<br>OTHER (SPECIFY NAME OF HEALTH FACILITY AND DISTRICT) (47):	

***INTERVIEWER**: "Lino ndilamubuzya kujatikizya buponi bwenu kucipanga hondi chabamatumbu. Amutole kaindi kaniini biyo kuyeeya bukkale bwenu mbubwakabede. Sena mwalibambila kutalika?"*

D7		Ciindi nomwakakkede kucipanga hondi cabamatumbu…	YES (1)	NO (0)	DON'T KNOW (96)
	A	SENA KWAKALI BULO NAANKA MATULESI ZYAKUTI MUBELESYA	☐	☐	☐
	B	SENA KULI NOMWAKABELESYA BULO NAANKA MATULESI AMUNTU UMBI KWA CIINDI CIMWI	☐	☐	☐
	C	SENA KULI NOMWAKOONA MUKASABWI KAMANSENYA CIINDI CAMASIKU	☐	☐	☐
	D	Sena baka mulailila kumilawo azyeeledwe kucita zya mung'anda yaba matumbu mbumwakasikila	☐	☐	☐
	E	SENA MWAKALIJISI KWAKUJANA MEENDA MABOTU	☐	☐	☐
	F	SENA MWAKALIJISI ZYAKUMUNISYA LYAMANA KUBBILA ZUBA?	☐	☐	☐
	G	SENA MWAKALIJISI KWAKUSAMBILA NAA KWAKUSAZYILA ZYIKOBELA	☐	☐	☐
	H	SENA KWAALI BUSENA BULIKABOTU/BUKWABILIDWE BWAKUBIKKA ZYINTU A ZYAKULYA ZYENU?	☐	☐	☐
	I	SENA KULI NOMWAKAIYA MAKANI AANSEBA	☐	☐	☐

NO.	QUESTION	POTENTIAL RESPONSES	SKIP
D8	Sena kwakali busena bubambilidwe kujikila kuchipanga hondi cabamatumbu?	Yes (1)<br>No (2)<br>Don't know (96)	If (0) or (96), skip to D10
D9	Sena busena oobu bwakujikila buli vumbidwe?	Yes (1)<br>No (2)<br>Don't know (96)	
D10	Sena kuli zyakucita zyipya zyomwakayiya kamuli kuchipanga hondi cabamatumbu?	Yes (1)<br>No (2)<br>Don't know (96)	If (0) or (96), skip to D12

SURVEY ID | | | | | | |

D11	Ino zyakucita nzi zyomakayiya?		
D12	Sena mwakaa mbilwa kuti musange mali (kwacha) akaambo kakukala mung'anda yabamatumbu?	Yes (1)<br>No (2)<br>Don't know (96)	If (0) or (96), skip to D14
D13	Ino mali nzi (kwacha) ngo mwakasanga aantomwe?		
D14	Sena mwakaambilwa kuti musange kufumbwa cintu kunze aamali kutegwa kukale mung'anda yaba matumbu?	Yes (1)<br>No (2)<br>Don't know (96)	If (0) or (96), skip to D16
D15	Ino ncinzi ncomwakasanga?<br><br>*(Select all that apply)*	LABOR (1)<br>LIVESTOCK/POULTRY (2)<br>FOOD OR OTHER AGRICULTURAL RESOURCES (3)<br>OTHER IN-KIND RESOURCES (SPECIFY) (4)<br>OTHER (SPECIFY) (5):	

D16	***INTERVIEWER:*** *"*Ono ndila mubuzya mapenzi azyizyilwe bamakaintu ngobaba angayo ajanika ku maanda abamakaintu ciindi nobakala ookuya kulindilila kutumbuka**.** Ndamba kupenzi alimwi, ndalomba mundaambile cilibwaceeci cakali penzi kulindinywe ciindi nimwakali kukala kung'anda yabamakaintu kamutana tumbuka, anaa kuti lyakali mpenzi pati na lyakali penzi syoonto kulindinywe.*"*				
		**MAJOR PROBLEM (2)**	**MINOR PROBLEM (1)**	**NO PROBLEM (0)**	**UNDECIDED (96)**
A	QUAZYOONSE MBOZI BEDE	☐	☐	☐	☐
B	KWEENDELEZYA ALIMWI AKU AZILUBILA	☐	☐	☐	☐
C	BULONDO AKUSALALA	☐	☐	☐	☐
D	BABELESI KUJANIKA	☐	☐	☐	☐
E	BABELESYI KUMVWANA AMBABO	☐	☐	☐	☐
F	KUKOZYA KUBA AAKWAKU JIKILA	☐	☐	☐	☐
G	KUKANDUKA	☐	☐	☐	☐
H	KUBA KWKUNYINA NTENDA	☐	☐	☐	☐
I	BUUMBA/BUKATA	☐	☐	☐	☐
J	TUNSIYA NSIYA TULIKABOTU	☐	☐	☐	☐

NO.	QUESTION	POTENTIAL RESPONSES	SKIP
D17	Mwaakkutila buti abukkale bwenu kucipanga hondi cabamatumbu?	VERY SATISFIED (1)<br>MORE OR LESS SATISFIED (2)<br>NOT SATISFIED (3)	

D17a	Hena muyeyela kuti muyakuyanda kubelesya chipanga hodi cabamatumbu, kukutumbuka ku mazuba abola.	YES (1)<br>NO (0)<br>DON'T KNOW (96)	
D17b	Hena ingamwakonzya kuzumizya benzinyoko na basimukowa kubelesya cipanga hodi cabamatumbu.	YES (1)<br>NO (0)<br>DON'T KNOW (96)	

***INTERVIEWER:** "Twalumba kwiingula mibuzyo yachipanga hondi cabamatumbu. Ino ndalikulombozya kuti tubandika alimwi amakani akutumbuka kwenu kwakainda ino-ino.."*

D18	Nguni wakagwasilizya ciindi cakutumbuka kwaku caaliuzya?<br><br>*(Select all that apply)*<br><br>*If respondent says NO ONE ASSSISTED, probe to determine whether any adults were present at the delivery.*	DOCTOR/CLINICAL OFFICER (1)<br>NURSE/MIDWIFE (2)<br>OTHER HEALTH FACILITY STAFF/PERSONNEL (3)<br>TRADITIONAL BIRTH ATTENDANT (4)<br>SMAG (5)<br>RELATIVE/FRIEND/AUNTIE (6)<br>NO ONE ASSISTED (7)<br>OTHER (SPECIFY) (8):	
D18a	Hena nkokuli nkomuyanda kuti mukatumbukile mwana ku mazuba aabola.	YOUR HOME (1)<br>OTHER HOME (2)<br>HEALTH POST/FACILITY (3)<br>HOSPITAL (4)<br>OTHER (SPECIFY) (5):	
D18b	Hena nkokuli komwakalibambilide kutumbukila mukutumba kwakusyalila ooku.	YOUR HOME (1)<br>OTHER HOME (2)<br>HEALTH POST/FACILITY (3)<br>HOSPITAL (4)<br>OTHER (SPECIFY) (5):	
D19	Nkuli nkumwaka tumbukila mwana wenu wakucaalizya?	YOUR HOME (1)<br>OTHER HOME (2)<br>HEALTH POST/FACILITY (3)<br>HOSPITAL (4)<br>OTHER (SPECIFY) (5):	If (1), (2) or (5), skip to D31
**INSTRUCTIONS:** If respondent answers **OTHER (5) to Question D19**, probe to ensure this is not a health post/facility or hospital.			

**KUTUMBUKILA KU NGÁNDA ILANGANYA NSEBA NAA CIBBADELA**			
**NO.**	**MUBUZYO**	**BWIINGUZI BULANGILWA**	**SOTOKA**
D20	Ino nkokuli kucibbaddela nkumwa kasaanguna kwinka kuyoo tumbuka da lyakucaalizya?	CHOMA DISTRICT<br>CHOMA GENERAL (801001)<br>MANGUNZA (801019)<br>MACHA MISSION (801002)<br>MASUKU MISSION (801021)<br>MBABALA (801022)	

	(INSTRUCTIONS: if woman reports hospital, probe to ensure she did not first present at a health facility and was transferred to hospital.)	MOCHIPAPA (801023)<br>SIMAKUTU (801043)<br>KALOMO DISTRICT<br>CHIFUSA HC (804023)<br>CHILALA HC (804024)<br>DIMBWE HC (804019)<br>HABULILE HC (804032)<br>KALOMO DISTRICT HOSPITAL (804002)<br>KANCHELE HC (804014)<br>MAWAYA HC (804034)<br>MOONDE HP (804042)<br>MUKWELA HC (804020)<br>SIACHITEMA HC (804013)<br>PEMBA DISTRICT<br>JEMBO (801413)<br>MUZOKA (801419)<br>NYIMBA DISTRICT<br>CHIPEMBE RHC (307010)<br>HOFMEYR ZONAL HC (307011)<br>KACHOLOLA RHC (307012)<br>MKOPEKA RHC (307016)<br>NYIMBA DISTRICT HOSPITAL (307001)<br>MANSA DISTRICT<br>FIMPULU (403017)<br>KABUNDA (403018)<br>LUBENDE (403041)<br>MANO (403026)<br>MANSA GENERAL HOSPITAL (403001)<br>MIBENGE (403029)<br>MUSAILA (403030)<br>MUTITI (403031)<br>MUWANGUNI (403032)<br>CHEMBE DISTRICT<br>KUNDAMFUMU (403023)<br>LUKOLA (403037)<br>LUNDAZI DISTRICT<br>CHIKOMENI (405026)<br>KAMSARO (305034)<br>KAPICHILA (305023)<br>LUKWISIZI (305040)<br>LUNDAZI HOSPITAL (305032)<br>LUSUNTHA (305021)<br>MWASE LUNDAZI ZONAL (305011)<br>NKHANGA (305046)<br>NYANGWE (305020)<br>PHIKAMALAZA (305031)<br>ZUMWANDA (305024)<br>OTHER (SPECIFY NAME OF HEALTH FACILITY AND DISTRICT) (47):	
D21	Ino mwakabelesya cakweendela camusyobo nzi kuya kucibbaddela ciindi nimwakainka kukutumbuka mwana wakucaalizya?	WALKING (1)<br>BICYCLE (2)<br>CARRIED IN WHEELBARROW (3)<br>ANIMAL-DRAWN CART (4)<br>TAXI (5)<br>CAR (6)<br>MOTORCYCLE (7)<br>AMBULANCE (8)<br>OTHER (SPECIFY) (9):	

D22	Kubelesya [ceendelo cimwi], cakamutolela ciindi cilamfu buti kuti musike ku ngánda ilanganya nseba naa cibbadela?<br><br>*Be sure to specify unit of response.*	HOURS MINUTES	
D23	Sena mwaka tumbuka mwana wenu kucibbaddela nkumwaka saanguna kwinka kuyoo tumbukila?	YES (1)<br>NO (0)<br>DON'T KNOW (96)	If (1) or (96) skip to D27
D24	Ino mwakatumbukila kucibbadela nzi?	CHOMA DISTRICT<br>CHOMA GENERAL (801001)<br>MANGUNZA (801019)<br>MACHA MISSION (801002)<br>MASUKU MISSION (801021)<br>MBABALA (801022)<br>MOCHIPAPA (801023)<br>SIMAKUTU (801043)<br>KALOMO DISTRICT<br>CHIFUSA HC (804023)<br>CHILALA HC (804024)<br>DIMBWE HC (804019)<br>HABULILE HC (804032)<br>KALOMO DISTRICT HOSPITAL (804002)<br>KANCHELE HC (804014)<br>MAWAYA HC (804034)<br>MOONDE HP (804042)<br>MUKWELA HC (804020)<br>SIACHITEMA HC (804013)<br>PEMBA DISTRICT<br>JEMBO (801413)<br>MUZOKA (801419)<br>NYIMBA DISTRICT<br>CHIPEMBE RHC (307010)<br>HOFMEYR ZONAL HC (307011)<br>KACHOLOLA RHC (307012)<br>MKOPEKA RHC (307016)<br>NYIMBA DISTRICT HOSPITAL (307001)<br>MANSA DISTRICT<br>FIMPULU (403017)<br>KABUNDA (403018)<br>LUBENDE (403041)<br>MANO (403026)<br>MANSA GENERAL HOSPITAL (403001)<br>MIBENGE (403029)<br>MUSAILA (403030)<br>MUTITI (403031)<br>MUWANGUNI (403032)<br>CHEMBE DISTRICT<br>KUNDAMFUMU (403023)<br>LUKOLA (403037)<br>LUNDAZI DISTRICT<br>CHIKOMENI (405026)<br>KAMSARO (305034)	

		KAPICHILA (305023)<br>LUKWISIZI (305040)<br>LUNDAZI HOSPITAL (305032)<br>LUSUNTHA (305021)<br>MWASE LUNDAZI ZONAL (305011)<br>NKHANGA (305046)<br>NYANGWE (305020)<br>PHIKAMALAZA (305031)<br>ZUMWANDA (305024)<br>OTHER (SPECIFY NAME OF HEALTH FACILITY AND DISTRICT) (47):	
D25	Sena mwakatumwa na kutolwa kucibbaddela eeci amubelesi waku cibbaddela?	YES (1)<br>NO (0)<br>DON'T KNOW (96)	If (0) or (96), skip to D27
D26	Ino kweezyeezya biyo, cakamutolela ciindi cilamfu buti kuti mweetwe kucibbadela eeci?	LESS THAN 1 HOUR (1)<br>1 TO 2 HOURS (2)<br>MORE THAN 2 HOURS (3)	
D27	Ino Nguni wakasungula lutaanzi kuti mutumbukile kucibbaddela?	YOURSELF (1)<br>HUSBAND/PARTNER (2)<br>MOTHER/MOTHER-IN-LAW (3)<br>AUNTIE (4)<br>OTHER FAMILY MEMBER (5)<br>FRIEND (6)<br>OTHER (SPECIFY) (7):	
D28	Sena nomwakamana kutumbuka mwakakkala kucibbadela kwamawoola asika makkumi obilo aone (24) naanka buzuba bomwe kamutana lekezyegwa?	YES (1)<br>NO (0)<br>DON'T KNOW (96)	

D29	***INTERVIEWER:*** *"Ndila lemba mundaandanda wa zya nseba zyobacita alimwi ndiyanda kuzyiba nakuti mwaka tambula nokuba kuta tambula kuli bwazyezyi kucibbaddela ciindi nimwakaainka kukutumbuka."*			
		**RECEIVED (1)**	**DID NOT RECEIVE (0)**	**DON'T KNOW (96)**
	A Kutumbuka kwakucaalizya, sena mwana wenu mwaka mutumbuka kubelesya nzila wakwaandulwa? Nkokuti, sena baka mwandula ada kutegwa bagwisye mwana?	☐	☐	☐
	B Ciindi nimwaka tumbuka lwaku caalizya sena mwaka pegwa bulowa?	☐	☐	☐
	C Misamu yama Antibiotics/intravenous (IV)meenda	☐	☐	☐
	D Kukulwaizyigwa kunyosya	☐	☐	☐
	E Zyintu zyibelesyegwa kucesya naa kutantaanya bana mumukwasyi/kulaililwa	☐	☐	☐
	F Kulaililwa makani akulanganya mwana muvwanda amakani aaku gumanya lukanda a lukanda	☐	☐	☐

D30	*INTERVIEWER: "Ono ndila mubuzya mapenzi azyizyilwe bamakaintu ngobaba angayo kucibbaddela ciindi banotumbuka. Ndila abandauka ndalomba mundaambile mbuli bwayaya akali mapenzi kuli ndinywe ciindi nimwakali kutumbuka kucibbaddela naa mbombubo nakuti akali mapenzi mapati na mapenzi masyoonto kuli ndinywe."*				
		**MAJOR PROBLEM (2)**	**MINOR PROBLEM (1)**	**NO PROBLEM (0)**	**UNDECIDED (96)**
	A BUBE BWA BUBAMBE BWA ZYANSEBA CIINDI CAKU TUMBUKA	☐	☐	☐	☐
	B BULEMU MBOBAPA BABELESYI BAZYABUUMI	☐	☐	☐	☐
	C BUSENA BUVUMBILILIDWE CIINDI CAKU TUMBUKA	☐	☐	☐	☐
	D BULONDO BWA CIBBADDELA	☐	☐	☐	☐
**After completing the facility-based delivery section, continue to MODULE E.**					

**HOME DELIVERIES**			
**No.**	**Question**	**Potential responses**	**Skip**
D31	Ino Nguni waka mbakuti mutumbukile kung'anda?	YOURSELF (1)<br>HUSBAND/PARTNER (2)<br>MOTHER/MOTHER-IN-LAW (3)<br>AUNTIE (4)<br>OTHER FAMILY MEMBER (5)<br>FRIEND (6)<br>OTHER (SPECIFY) (7):	
D32	Twaambo nzi tupati-pati twakapa kuti mutatumbukili kucibbadela?<br><br>*Select all that apply.*	COST TOO MUCH (1)<br>FACILITY NOT OPEN (2)<br>TOO FAR/NO TRANSPORTATION (3)<br>POOR QUALITY SERVICE/DON'T TRUST (4)<br>NO FEMALE HEALTH PROVIDER (5)<br>HUSBAND/FAMILY DIDN'T ALLOW (6)<br>SHORT LABOR (7)<br>BABY CLOTHES (8)<br>CDK (9)<br>NO MOTHERS SHELTER (10)<br>NOT NECESSARY (11)<br>NOT CUSTOMARY (12)<br>OTHER (SPECIFY) (13):	
D33	Sena mwakainka kucibbadela kuya kupimwa nseba yenu aya mwana wenu mukati mwamawoola makkumi obilo aone nomwakamana kutumbuka?	YES (1)<br>NO (0)<br>DON'T KNOW (96)	

MODULE E: SPENDING AND SAVINGS

SURVEY ID | | | | | | |

INTERVIEWER: *"Ino ndilamubuzya makani aamali ngomwakabelesya ada lyaainda akukutumbuka. Amuyeeye mali ngomwakabelesya kujatikizya da akutumbuka kwenu ambomwakalibambila kujana mali aayo ngomwakabelesya"*

E1	***SIKUBIZYA:*** *"Ino ndilombozya kuti tubandike makani ajatikizya mali ngomwakabelesya mukutumbuka kwenu kwakainda ino-ino. Amuyeeye zyintu zyalo zyomwakaula mukulibambila kutumbuka, mulweendo lwenu kuya kucibbadela na kungánda nkomwakaakutumbukila, mpoona akuciindi cakutumbuka kwini kucibbadela naa kungánda nkomwakatumbukila. Sena mwalibambila kutalika?"*				
	Kweezyeezya biyo mwakabelesya mali nzi:		**AMOUNT (KWACHA)**	**NONE (0)**	**DON'T KNOW (96)**
	Mu kulibambila:				
	A	ZYAKUBELESYA KUTUMBUKA (Mbuli zyakusama kumaanza banootumbusya, silinji, zipepa zyakuyala, mbeli, zyakujaizya tuuka tweeta malwazi, azimwi zyatambwa.)			
	B	ZYISANI ZYAMWANA/KAPAYI			
	Mu lweendo lwenu:				
	C	CEENDELO CAKUUNKILA AKUJOKA (kuti paang'nda yabo bikka 0)			
	D	CHIPANGA HODI CABAMATUMBU KUCIBBADELA/KWAKUKKALA KUMBI NOMWAKALI KULINDILA KUTUMBUKA			
	Aciindi cakutumbuka:				
	E	SIKUTUMBUSYA/MALI ABBADELWA KUCIBBADELA			
	F	KUBBADELA KUMWI KUTALEMBEDWE MUMULAWO			
	G	MALI AKUPA MUNTU KULUMBA KUTI WAGWASYA			
	H	KUBBADELA KUTALI MUMALI (ezyeezya kuti inga aba malinzi muma kwacha)			
	I	MISAMU			
	J	KUPIMWA MUBULI			
	K	KUBBADELA KUMWI BIYO			

**NO.**	**QUESTION**	**POTENTIAL RESPONSES**	**SKIP**

SURVEY ID

E2	Sena mwakalijisi mali akayoboledwe akukuyoobelesya kukutumbuka kwenu kwaka caalizya?	YES (1)<br>NO (0)<br>DON'T KNOW (96)	If (0) or (96), skip to E8
E3	Sena mukweezyeezya kwenu mwakaliyobwede mali akwana kubelesya mukutumbuka kwenu kwakainda ino-ino (mukulibambila, mu lweendo, a ciindi cakutumbuka)?	YES (1)<br>NO (0)<br>DON'T KNOW (96)	
E4	Ino nkokuli nkumwaka yoobwede mali aaya?	AT YOUR HOME (1)<br>AT A FRIEND OR FAMILY MEMBER'S HOME (2)<br>IN A BANK ACCOUNT (3)<br>OTHER (SPECIFY) (4):	
E5	Sena kuli umwi wamung'anda yenu (mbuli mbomukwetene limwi) kwakabwene mali ngumwaka yoobwede?	YES (1)<br>NO (0)<br>DON'T KNOW (96)	
E6	Ino lyakali lipati buti da lyenu nomwakatalika kuzungilila mali?	WEEKS<br><br>MONTHS	
E7	Sena kuli wakabikka mali kumugwasya kusungilila (mukulibambila, mu lweendo, a ciindi cakutumbuka), nakwali, nguni?<br><br>*(Select all that apply)*	HUSBAND/PARTNER (1)<br>YOUR CHILDREN (2)<br>PARENT/GRANDPARENT (3)<br>OTHER FAMILY MEMBER (4)<br>FRIEND (5)<br>AUNTIE (6)<br>NO ONE (7)<br>OTHER (SPECIFY) (8):	
E8	Sena ciyandika buti kusungilila mali aakubelesya kukutumbuka?	NOT IMPORTANT (1)<br>SLIGHTLY IMPORTANT (2)<br>MODERATELY IMPORTANT (3)<br>IMPORTANT (4)<br>VERY IMPORTANT (5)	
E9	Sena kuli nomwakayobwede mali ku bbanga?	YES (1)<br>NO (0)<br>DON'T KNOW (96)	
E10	Sena kuli nomwakatumide mali kubesya luwaile lwa mumaanza?	YES (1)<br>NO (0)<br>DON'T KNOW (96)	In (0) or (96), skip to module F
E11	Sena kuli nomwakatumide mali kubesya luwaile lwa mumaanza?	HUSBAND/PARTNER (1)<br>YOUR CHILDREN (2)<br>PARENT/GRANDPARENT (3)<br>OTHER FAMILY MEMBER (4)	

		FRIEND (5)<br>AUNTIE (6)<br>OTHER (SPECIFY) (7):	

MODULE F. POST-NATAL CARE

***INTERVIEWER:** "Ino ndilombozya kuti ndimubuzye mibuzyo misyoonto biyo iijatikizya naa kuli kubambwa nkumwakatambula kwanseba yenu aya mwana wenu nomwakamana kutumbuka kwamamanino."*

NO.	QUESTION	POTENTIAL RESPONSES	SKIP
F1	Sena nomwakamana kutumbuka kwaino-ino mwakaunka kucibbadela kuya kupimwa ciindi cikkwana mawoola makkuni obile aone kacitanainda?	YES (1)<br>NO (0)<br>DON'T KNOW (96)	If (0) or (96) skip to F6
F2	Sena mwakaunka kucibbadela kuya kupimwa nokwakainda mazuba otatwe aakutumbuka kwaainda ino ino?	YES (1)<br>NO (0)<br>DON'T KNOW (96)	
F3	Sena mwakaunka kucibbadela kuya kupimwa nokwakainda sondo yomwe naa zyobilo kuzwa nomwakamana kutumbuka kwaainda ino ino?	YES (1)<br>NO (0)<br>DON'T KNOW (96)	
F4	Sena mwakaunka kucibbadela kuya kupimwa nozyakainda nsondo zili cisambomwe nomwakamana kutumbuka?	YES (1)<br>NO (0)<br>DON'T KNOW (96)	

F5	***Sikubuzya:** "Ono ndila mubuzya mibuzyo iimwi yama penzi aazyizyilwe kububambe bwa bamakaintu mbobajana kuzibbaddela baunka kuyoopimwa mukati ka nsondo zili cisambomwe kwakuzwa akutumbuka. Ndani kwaamba kuli cimwi acimwi, ndalomba mundaambile naa cili boobu cakakuli penzi kuli ndinywe ciindi nimwakaunka kucibbaddela mukatika nsondo zili cisambomwe kuya kupimwa ciindi nimwaka tumbuka lwaakucaalizya, na iiyi lyakali penzi pati, na lyakali penzi lisyoonto kuli ndiywe."*				
		**MAJOR PROBLEM (2)**	**MINOR PROBLEM (1)**	**NO PROBLEM (0)**	**UNDECIDED (96)**
A	CIINDI NCIMWAKATOLA KUTEGWA MUBONANE ABA GWASILIZYA	☐	☐	☐	☐
B	KUKOZYA KUKANANA PENZI NA ZYAKULIBILIKA ZYA DA LYENU	☐	☐	☐	☐
C	BUPANDULUZI BWA PENZI LYA NSEBA.	☐	☐	☐	☐
D	KUKOZYA KUSISILILA KUTEGWA BAMWI BATABONI ZYAPIMWA.	☐	☐	☐	☐

	E	KUSISILILA KUKOZYA KUKANANA KUTEGWA BAMWI BATAVWI CIKANANWA MUMUBANDI WESU	☐	☐	☐	☐
	F	BULONDO BWA CIBBADDELA	☐	☐	☐	☐
	G	BAKAMULANGANYA BUTI BABELSI?	☐	☐	☐	☐
	H	MALI ABBADDELWA KUTEGWA MUBONYWE NA KUSILIKWA	☐	☐	☐	☐

F6	Sena kwaino kuli ncomubelesya na kulinzila yakumusya kumita nokuba kutamita?	YES, MODERN METHOD (1)<br>YES, TRADITIONAL METHOD (2)<br>NO (0)<br>N/A, CURRENTLY PREGNANT (3)<br>DON'T KNOW (96)	
F7	**INSTRUCTIONS:** Look back to question **B27-** Sena mwana wakatumbukwa ucipona?<br><br>*Confirm with respondent.*	YES (1)<br>NO (0)<br>DON'T KNOW (96)	If (0) or (96) skip to F15
F8	Sena mucimunyosya mwana wenu ngumwaka kacaalizya kuzyala?	YES (1)<br>NO (0)<br>DON'T KNOW (96)	If (0) or (96) skip to F10
F9	Sena eno mula musanina mwana zyakulya zimbi kunze amukupa waku nkolo amisamu?	YES (1)<br>NO (0)<br>DON'T KNOW (96)	
F10	Ino munsondo zyobilo zyainda kuli nomwakatola mwana kukubambwa nseba yakwe?	YES (1)<br>NO (0)<br>DON'T KNOW (96)	If (0) or (96), skip to F12
F11	Ino nkuli nkomwakatola mwana wenu ciindi cakusaanguna kukubambwa nseba?	HEALTH CARE CENTER (1)<br>HOSPITAL (2)<br>PHARMACY (3)<br>TRADITIONAL HEALER (4)<br>OTHER (SPECIFY) (5):	
F12	Sena mwana wenu wakapegwa cipimo cabana (ntomba)?	YES (1)<br>NO (0)<br>DON'T KNOW (96)	If (0) or (96), skip to F15

F13	**INSTRUCTIONS:** Based on **D1**, calculate child's age.<br><br>*Specify unit of response.*				
	**INSTRUCTIONS:** Ask to see the child's vaccination card. If available, use card to confirm the vaccines received and mark below. If card is unavailable, ask mother which vaccines the child has received. **BASED ON CALCULATED AGE FROM F13**, ask only about **AGE APPROPRIATE** vaccines.				
F14	Confirm you have the child's vaccine card in-hand.	YES (1)<br>NO (0)			
		**CONFIRMED BY VACCINE CARD**		**CONFIRMED BY MOTHER**	
		RECEIVED	NOT RECEIVED	RECEIVED	NOT RECEIVED
	**Sena mwana wenu wakatambula bukwabilizyi butobela naakazyalwa**				
	A BCG	☐	☐	☐	☐

SURVEY ID ☐☐☐☐☐☐

	B Polio (OPV-0)	☐	☐	☐	☐
	**Sena mwana wenu wakatambula bukwabilizyi butobela bupegwa mwana akwanya nsondo cisambomwe?**				
	C Polio (OPV-1)	☐	☐	☐	☐
	D DTP-HepB-Hib-1	☐	☐	☐	☐
	E Pneumococcal (PCV)	☐	☐	☐	☐
	F Rotavirus	☐	☐	☐	☐
	**Sena mwana wenu wakatambula bukwabilizyi butobela bupegwa mwana akwanya nsondo zyili kkumi?**				
	G Polio (OPV-2)	☐	☐	☐	☐
	H DTP-HepB-Hib-2	☐	☐	☐	☐
	I Pneumococcal (PCV)	☐	☐	☐	☐
	J Rotavirus	☐	☐	☐	☐
	**Sena mwana wenu wakatambula bukwabilizyi butobela bupegwa mwana akwanya nsondo zyili kkuni azyone?**				
	K Polio (OPV-3)	☐	☐	☐	☐
	L DTP-HepB-Hib-3	☐	☐	☐	☐
	M Pneumococcal (PCV)	☐	☐	☐	☐

Interviewer: *"*kumibuzyo iitobela, amuvwiile nakuti muvwa kuti inga mwacita oobo, bwiinguzi bwenu bwakulipa biya.*"*			
F15	Mwakapimwa bulwazi mwasikalileke ciindi ni mwaala da lyakucaalizya?	YES (1)<br>NO (0)<br>PREFER NOT TO ANSWER (2)<br>DON'T KNOW (96)	
F16	Ino bube bwenu buli buti kubulwazi bwasikalileke?	INFECTED (1)<br>NOT-INFECTED (2)<br>PREFER NOT TO ANSWER (3)<br>DON'T KNOW (96)	If (2), (3), or (96), skip to F23
F17	Sena mwakanywa musamu wama ARV ciindi nimwalaada lyakucaalizya?	YES (1)<br>NO (0)<br>DON'T KNOW (96)	
F18	**INSTRUCTIONS:** Refer back to question **B27-28**. Did the respondent's baby survive beyond the day of birth?<br><br>*Confirm with respondent.*	YES (1)<br>NO (0)<br>DON'T KNOW (96)	If (0) or (96), skip to Module G
F19	Sena mwana wenu wakanywa ma ARVS nokwainda nsondo zyili cisambomwe zyakuzyalwa.	YES (1)<br>YES, BUT BABY DIED BEFORE 6 WEEKS OF AGE (2)<br>NO (0)<br>DON'T KNOW (96)	
F20	Sena mwana wenu wakapimwa bulwazi bwakazunda kasikalileke.	YES (1)<br>NO (0)<br>DON'T KNOW (96)	If (0) or (96), skip to F23
F21	Wakalaa nsondo zyakuzyalwa zyongaye ciindi nakapimwa bulwazi bwasikalileke?		

SURVEY ID | | | | | | |

	Round to nearest number		
F22	Ino ncinzi cakajanywa kukupimwa ciindi mwana wenu nakapimwa bulwazi bwasikalileke?	INFECTED (1)<br>NOT INFECTED (2)<br>PREFER NOT TO ANSWER (3)<br>DON'T KNOW (96)	

F23	Mu mazuba otatwe ainda, sena nywebo nokuba bamung'anda yenu balaa mwaka kumi lyomwe ayosanwe atala kuli wakalipa kucita zyimwi mbuli bwazyezyi amwana?<br><br>*Select all that apply*			
		**YES (1)**	**NO (0)**	**DON'T KNOW (96)**
A	KUMUBALILA BBUKU NA KULANGA ZYIFANIKISO MUMABBUKU AMWANA	☐	☐	☐
B	KU MWANINA TWAANO MWANA	☐	☐	☐
C	KWIMBA TWIMBO NA TWAKUMBULIZYA ANGUWE MWANA	☐	☐	☐
D	KUGWISYA MWANA ANZE AANG'ANDA, MUMUNZI, MULUBUWA, NA MUYAKILIDWE	☐	☐	☐
E	MAZINA, KUBALA NAMBA, NA KWEENGA ZINTU AMWANA	☐	☐	☐

F24	***INTERVIEWER:*** *"For the following questions, please respond only if you feel comfortable doing so. Your response is optional. I am going to read you a list of problems. Please tell me how often each of these problems has happened to you in the PAST TWO WEEKS: never, once in a while, more than half the time, or almost always."*				
		**NEVER (0)**	**ONCE IN A WHILE (1)**	**MORE THAN HALF THE TIME (2)**	**ALMOST ALWAYS (3)**
A	MVWIKI ZYOBILO ZYAINDA NDALIUSIDE NEKUBA KUTAKOMANA	☐	☐	☐	☐
B	MVWIKI ZYOBILO ZYAINDA HEKALIKUYANDA KUCITA CILICONSE (ZYAKUCITACITA, KUBELEKA, BANTU).	☐	☐	☐	☐
C	MVWIKA ZYOBILO ZYAINDA, NDAKALILA	☐	☐	☐	☐
D	MVWIKI ZYOBILO ZYAINDA, NDAKALENDELELWA	☐	☐	☐	☐

F25	***INTERVIEWER:*** *"Now I am going to read you a list of things that you may have experienced. Please tell me how often each of these events have happened to you in the past two weeks: never, once in a while, a few times, or many times. Again, please respond only if you feel comfortable doing so. Your response is optional."*				
		**NEVER (0)**	**ONCE (1)**	**A FEW TIMES (2)**	**MANY TIMES (3)**

	A	MUKWIKI ZYOBILO ZYAINDA-ZYINDI ZYONGAYE, BALUMI BENU, MUSAKWA WENU NAKAMUTONKENDE OLO KUMUMA LUBAI?	☐	☐	☐	☐
	B	MUVWIKI ZYOBILO ZYAINDA, ZYINDI ZYONGAYE BALUMI BENU, MUSANILWA WENU, NAKAMULYATIDE, KUMUKWELA, KUMUMA, KUMUSINA, NSEKUBA KUMUMPA?	☐	☐	☐	☐

MODULE G. LAST PREGNANCY

INTERVIEWER: Ino Ndamubuzya mibuzyo imwe iigaminina da lyenu kusikila kukutumbuka kwaino ino. waciindi cisyoonto biyo, amuyeeya nomwakazyiba kuti mulaada lwakusaanguna antoomwe a kupimwa/kulagwalagwa da. Sena mwalibambila kutalika?

NO.	QUESTION	POTENTIAL RESPONSES			SKIP
**Interviewer**: *Ask to see if antenatal care card is available for the woman's last pregnancy that led to a delivery and confirm information provided by respondent.*					
G1	Did the woman provide you with her antenatal card?	YES (1)<br>NO (0)<br>DON'T KNOW (96)			
G2	Mwakapimwa ziindi zyongaye kucibbadela naaka busena bwa nseba ku da lyenu lyakainda ino-ino?	NONE (0)<br>ONE TIME (1)<br>TWO TIMES (2)<br>THREE TIMES (3)<br>FOUR TIMES (4)<br>MORE THAN FOUR TIMES (5)			If (0) skip to End of Survey
G3	Sena mwakakanana kuli bwazyeezyi ciindi nimwa kainka kucipimo cada:	**YES (1)**	**NO (0)**	**DON'T KNOW (96)**	
	A Nkomuti katumbukile mwana?	☐	☐	☐	
	B Ncomunga mulacita naa kuti kwaba calubila?	☐	☐	☐	
	C Kubikilila mali akubelesya ciindi da lyabuka akuyoo tumbuka?	☐	☐	☐	
	D Nomwakali kulangilwa kutumbuka	☐	☐	☐	
G4	Sena muciyeeya ciindi ncomwakaambilidwe kuti ncomuyoo langilwa kutumbuka?	YES (1)<br>NO (0)<br>DON'T KNOW (96)			If (0), skip to G6
G5	Ino ndilili nomwakali kulangilwa kutumbuka?<br>(DD MONTH YYYY)	___ ___ ___ ___ ___ ___ ___ ___<br>D D M M Y Y Y Y			

SURVEY ID

	If EDD is on ANC card, copy it from card. If no card and date not know, enter 15th		
G6	Lyakali nsondo zyongaye da lyenu ciindi nomwa kainaka koolangwa ciindi cakusaanguna?<br><br>*Please specify unit of response.*	WEEKS<br><br>MONTHS	

INTERVIEWER: Twasika Kumamanino amibuzyo. Twalumba kutupa ciindi cakuti muingule mibuzyo eeyi.

Sena kuli zyakwaamba zyakuyungizya zyomulijisi na?

G7	Hena nga mulazumina kuti omwe wa ndiswe akamuswaye buzuba buli boonse munsondo ziboola kuti akavyuntauzye tumwi tumwi?	YES (1)<br>NO (0)	

COMMENTS:

END OF SURVEY

INSTRUMENT REVIEW

Enumerator Initials:	
Date (DD/MM/YYYY)	
Supervisor Initials:	
Date (DD/MM/YYYY)	

Data Entry Initials:	
Date (DD/MM/YYYY)	
Supervisor Initials:	
Date (DD/MM/YYYY)	
