# Supplementary material for: Characteristics of maternity waiting homes and the women who use them: Findings from a baseline cross-sectional household survey among SMGL-supported districts in Zambia
Source: PLoS One. 2018 Dec 31;13(12):e0209815. doi: 10.1371/journal.pone.0209815 (PMC6312364; doi:10.1371/journal.pone.0209815)
Supplement: S3 File — Household Survey Baseline Impact Evaluation–Bemba. (PDF) [file pone.0209815.s003.pdf]

SURVEY ID | | | | | | |

<u>Instrument ID</u>:

The MAHMAZ Project
# – Household Survey Baseline Impact Evaluation **BEMBA**

Abakuminwe ne ilisambililo:

Abanakashi abapapile abana mumyenshi ikumi limo na ibili iyapita kunuma, abakwete imyaka yakufyalwa ukwambila pali 15 nokuya pamulu abekala muncende ishipalamine nama catchment areas ya fipatala ifili muli ilisambililo

SHORT SCREEN

SS1	Nibanga bana mayo abali nemyaka ukwambila pamakumi ikumi na fisano (15) ukufika pamakumi yane na pabula (49) abekela muli ino n'ganda ukubikapo nabo abafwile or moved away mu mwaka wapwile?<br><br>*Write down the number of women.*	☐☐	If none, thank person and move to next household.
SS2	Bushe pali banamoya pali aba epobapapile papita umwaka nagulu mu mweshi ikumi limo na cibili?	YES (1)<br>NO (0)<br>DON'T KNOW (96)	If (0) or (96), thank person and move to next household
SS3	Bushe namayo ngatali pa ng'anda kuti mwatwasukilako amepusho pa lwa kwimita no kupaapa kwakwe?	YES (1)<br>NO (0)<br>DON'T KNOW (96)	
INTERVIEWER: IF YOU HAVE ANSWERED YES TO SS1 AND YES TO SS2, THEN PROCEED WITH THE INFORMED CONSENTING PROCESS. PLACE THE UNIQUE ID STICKER ON THE INSTRUMENT AND ON THE HOUSEHOLD CONSENT FORM A.			
	Confirm consent was granted<br><br>*Draw a check mark if consent was granted.*		
IF CONSENT WAS GRANTED, PLACE A SECOND UNIQUE ID STICKER ON THE PAPER VERSION OF THE INSTRUMENT.			

SURVEY ID | | | | | | |

MODULE A. LOCATION

INSTRUCTIONS: Complete before administering the rest of the survey

NO.	FIELD	CODE	RESPONSE
A1	Province	EASTERN (1)<br>SOUTHERN (2)<br>LUAPULA (3)	
A2	District	CHOMA (1)<br>KALOMO (2)<br>NYIMBA (3)<br>PEMBA (4)<br>LUNDAZI (5)<br>MANSA (6)<br>CHEMBE (7)	
A3	Health Facility Catchment Area	CHOMA DISTRICT<br>CHOMA GENERAL (801001)<br>MANGUNZA (801019)<br>MACHA MISSION (801002)<br>MASUKU MISSION (801021)<br>MBABALA (801022)<br>MOCHIPAPA (801023)<br>SIMAKUTU (801043)<br>KALOMO DISTRICT<br>CHIFUSA HC (804023)<br>CHILALA HC (804024)<br>DIMBWE HC (804019)<br>HABULILE HC (804032)<br>KALOMO DISTRICT HOSPITAL (804002)<br>KANCHELE HC (804014)<br>MAWAYA HC (804034)<br>MOONDE HP (804042)<br>MUKWELA HC (804020)<br>SIACHITEMA HC (804013)<br>PEMBA DISTRICT<br>JEMBO (801413)<br>MUZOKA (801419)<br>NYIMBA DISTRICT<br>CHIPEMBE RHC (307010)<br>HOFMEYR ZONAL HC (307011)<br>KACHOLOLA RHC (307012)<br>MKOPEKA RHC (307016)<br>NYIMBA DISTRICT HOSPITAL (307001)<br>MANSA DISTRICT<br>FIMPULU (403017)<br>KABUNDA (403018)<br>LUBENDE (403041)<br>MANO (403026)<br>MANSA GENERAL HOSPITAL (403001)<br>MIBENGE (403029)<br>MUSAILA (403030)<br>MUTITI (403031)<br>MUWANGUNI (403032)	

SURVEY ID | | | | | | |

		CHEMBE DISTRICT<br>KUNDAMFUMU (403023)<br>LUKOLA (403037)<br>LUNDAZI DISTRICT<br>CHIKOMENI (405026)<br>KAMSARO (305034)<br>KAPICHILA (305023)<br>LUKWISIZI (305040)<br>LUNDAZI HOSPITAL (305032)<br>LUSUNTHA (305021)<br>MWASE LUNDAZI ZONAL (305011)<br>NKHANGA (305046)<br>NYANGWE (305020)<br>PHIKAMALAZA (305031)<br>ZUMWANDA (305024)	
A4	Village Name<br><br>*Write in the name of the village*		
**GPS COORDINATES, TAKE 1**			
A5	Latitude		
A6	Longitude		
**GPS COORDINATES, TAKE 2**			
A7	Latitude (decimal format)		
A8	Longitude (decimal format)		
A9	Date of Interview (DD/MM/YYYY)		
A10	Start time of interview<br>(24:00 format)		

SURVEY ID | | | | | | |

MODULE B. HOUSEHOLD ENUMERATION

INSTRUCTIONS: Confirm that the person who you are speaking with is the head of the household or the head woman of the household.

INTERVIEWER: *"Nalaipusha ifipusho pali imwe naba pa ng'anda. Pa mulandu waifi ifipusho, lekeni tuloshe ing'anda nga kabungwe ka bantu abapalupwa nangula abashili ba lupwa abekala pamo pa ncende, apaba umuntu umo ngo mukulu wa ng'anda uulolesha pa fya mipikile no kulya".*

NO.	QUESTION	POTENTIAL RESPONSES	SKIP
B1	Muli ne myaka in'ga iya kufyalwa?		
B2	Mwalipitako ku sukulu?	YES (1)<br>NO (0)<br>DON'T KNOW (96)	If (0) or (96), skip to B4
B3	Mwapelela mu grade shani?<br><br>*Write grade level (ie: 03 for grade 3). If <1 year completed, write down 00. If >12 years completed, write down 13.*	[ ][ ]<br>DON'T KNOW (96)	
B4	Lukuta nshi mulongana ko?	CATHOLIC (1)<br>PROTESTANT (2)<br>MUSLIM (3)<br>OTHER (SPECIFY) (4)	
B5	Muli mutundu nshi?		
B6	Bushe mwabashani pa lwakulosha ku kupwa?	MARRIED/COHABITING (1)<br>DIVORCED (2)<br>SEPARATED (3)<br>WIDOWED (4)<br>NEVER-MARRIED (5)	If (2), (3), (4), or (5), skip to B8
B7	*If respondent is the male head of household:*<br>Nibanga pamo abakashi nangula abanenu ba pa mutima mwakwata?<br><br>*If respondent is NOT male head of household:*<br>Nibanga pamo abakashi nagula abatemwikwa bapa mu tima umwine wa ng'anda umwaume akwata?		
B8	Ni banga ba kalume na bana abanakashi abakwete imyaka ya kufyalwa iyi shicilile iyi sano abekala mu ng'anda yenu?<br><br>*Include children who are in boarding school at the moment. If none, write down 00.*	[ ][ ] BOYS<br>[ ][ ] GIRLS	

SURVEY ID

B9	Ni banga ba kalume na bana abanakashi aba kwete imyaka ya kufyalwa iyisano (5) ukufika na kwi kumi limo na fine (14) abekala mu ng'anda yenu?<br><br>*Include children who are in boarding school at the moment. If none, write down 00.*	BOYS<br>GIRLS	
B10	Ni banga abaume na bana mayo abakakweta imyaka ya kufyalwa ikumi na fisano (15) ukufika na pamakumi yane na pabula (49) abekala pa ng'anda yenu?	MEN<br>WOMEN	
	Ni banga abaume na bana mayo abakakweta imyaka ya kufyalwa ikumi na fisano (15) ukufika na pamakumi yane na pabula (49) abekala pa ng'anda yenu? Mubikeko naba tushile muli uyu mwaka wapitile.	MEN<br>WOMEN	
B11	Ni banga abaume na banakashi abakweta imyaka ya kufyalwa amakumi yasano (50) ukufika na pa makumi mutanda na fine (64) abekala pa ng'anda yenu?	MEN<br>WOMEN	
B12	Ni banga abaume na bana mayo abacilile imyaka ya kufyalwa amakumi mutanda na fisano (65) abekala pa ng'anda yenu?	MEN<br>WOMEN	
INSTRUCTIONS: Count and record the total number (B8 to B12) of household members.			
B13	Moneni ukuti impendya yabantu pa gánda namupenda iya kumanina	YES (1)<br>NO (0)	

SURVEY ID | | | | | | |

INSTRUCTIONS: Ask the respondent to list the names of all women aged 15-49 in the household, including those who passed away in the last 12 months (1 year). Emphasize that you are also looking for information on individuals who have passed away in the last 12 months (1 year). Fill out column A with all names provided, and then continue to answer B-F for each person before selecting a respondent.

INTERVIEWER: *"Nomba kuti natemwa ukuti mulumbule amashiana yaba namayo bonse abali nemyaka yakufyalwa ikumi limo na fisano (15) ukufika na pama kumi yane na pabula (49) abekala pali ino ng'anda . Mubikepo nabafwile muli uyu mwaka wa pita".*

TABLE 1. ROSTER OF WOMEN AGED 15-49 YEARS

	A. Mukwai njebeni amashina ayakupelwa and age ayabo bonse bana mayo abakwete imyaka ya kufyalwa ikumi limo na fisano ukufika napa makumi yane na pabula abakela muli ino n'ganda for at least 4 days a week, mubikeko naba tushile muli uyu mwaka wapitile.<br><br>Ensure the number includes those who would have been living there if they didn't pass away/move away in the past 12 months.<br>*The number of women in this list should be greater or equal to the number of women in **B10**.*	B. Mumwaka wapita, mwalikwetepo ifumo ilya kokwele ukufika na kumilungu amakumi yatatu na fisano?<br><br>*That is, was (name) pregnant at least up until ~3 weeks before her estimated delivery date?*<br><br>*Instructions: Note that this includes a delivery, still birth, neonatal death, etc.at any point within the past 12 months*<br><br>YES (1)<br>NO (0)<br>DON'T KNOW (96)<br><br>*If (0) or (96), skip to next person.*	C. Uyo (*name) uwakweta ifumo acili uwamweo?<br><br>YES (1)<br>NO/DON'T KNOW (0)<br><br>*If (1), skip to E.*<br><br>**Check list and input name*	D.Kuti mwaipelesha or somebody else ukwasuka amepusho aya nono balyabo abakweta ifumo?<br><br>YES (1)<br>NO (0)<br><br>*If (0), skip to next person.*	E. Is (*name) potentially eligible to take the survey?<br><br>*If B=1 and (IF APPLICABLE) D=1, mark the box below.*<br><br>**Check list and input name*	**AFTER ALL WOMEN HAVE BEEN LISTED, TO SELECT A RESPONDENT:**<br><br>1. Roll the die<br><br>2. From the 1st checked box in Column E, count up to the rolled number, beginning again at the 1st checked box if needed until number is reached<br>3. Roll the die again<br>4. From the checked box you landed on after the 1st roll, count up to the 2nd rolled number, beginning again at the 1st checked box if needed until the 2nd number is reached<br>5. Select this woman<br>6. If woman selected is ALIVE, proceed to **Question B24**<br>7. If woman selected is DECEASED, proceed to Proxy Household Survey
B14					☐	
B15					☐	
B16					☐	
B17					☐	
B18					☐	
B19					☐	

SURVEY ID

B20					☐	
B21					☐	
B22					☐	
B23					☐	

SURVEY ID | | | | | | |

NO.	QUESTION	POTENTIAL RESPONSES	SKIP
B23A	How old is (name)?	[ ][ ]	
B24	Bushe umuntu musampwile epwali pakuti ansendemo ulubali mu kufwailikisha?	YES (1)<br>NO (0)<br>DON'T KNOW (96)	If (1), skip to consent then proceed to B27
B25	Kuti twapekanya inshita yimbi pakuti tukese limbi elyo bakabapo?	YES (1)<br>NO (0)<br>DON'T KNOW (96)	If (0) or (96), resample from potentially eligible women in TABLE 1
B26	Were you able to reschedule another time?	YES (1)<br>NO (0)	If (0), resample from potentially eligible women in TABLE 1
If you are unable to reschedule a time to come back and survey the sampled woman, go back to TABLE 1 and resample another potentially eligible woman. If you are re-visiting the household a subsequent time and the woman is now available, **proceed from Question B27**.			

INSTRUCTIONS: Make sure to obtain consent or assent (if the sampled woman is 15, 16 or 17 years old – refer to B23A), including a signature, from the sampled woman. If the woman is not able to sign, please have the woman provide a thumbprint. These questions will determine whether or not the sampled woman is eligible to proceed to the full household survey. If she is ineligible, then re-sample from Roster Table 1. If there are no more potentially eligible women to sample from, thank the woman and move on to the next household. If she is eligible, proceed to Module C.

STOP: MAKE SURE CONSENT OR ASSENT WAS OBTAINED FROM (NAME). PLACE A THIRD UNIQUE ID STICKER ON THE CONSENT FORM B – FOR THE ELIGBLE WOMAN. IF SHE HAS ALREADY CONSENTED AS THE HOUSEHOLD HEAD, NO NEED TO ISSUE A CONSENT FORM B.

INTERVIEWER: Namutasha pa kusumina ukusendamo ulubali muli uku ukufwailikisha. Nalamwipusha amepusho ayanono pa kupaapa mwakwete.

NO.	QUESTION	POTENTIAL RESPONSES	SKIP
B27	Bushe umwana mwekwete acili wamweo?	YES (1)<br>NO (0)<br>DON'T KNOW (96)	If (1), continue to Module C<br>If (96), skip to B29
B28	Nililali umwana wenu afwile?	BEFORE OR ON DAY OF DELIVERY (1)<br>WITHIN ONE MONTH AFTER DELIVERY (2)<br>OTHER (3)	If (2) or (3), continue to Module C
B29	Bushe mwa paapile bwangu ukwabula ukufika panshita iya ku paapilapo?	YES (1)<br>NO (0)<br>DON'T KNOW (96)	If (0), continue to Module C<br>If (96), end and re-sample from Roster Table 1
B30	Milungu inga yashalako ku cipimo elyo mwa paapile umwana?	<= 3 WEEKS (1)<br>>3 WEEKS (2)<br>DON'T KNOW (96)	If (1), continue to Module C<br>If (2), end and re-sample from Roster Table 1<br>If 96, end and re-sample from Roster

SURVEY ID | | | | | | |

MODULE C. DEMOGRAPHICS

INSTRUCTIONS: After eligible respondent has been randomly sampled from all eligible respondents, proceed with the instrument. Ensure that the woman selected to proceed with the survey has delivered a child **within the last year**. This section is to get basic demographics on the household and the respondent.
INTERVIEWER: "Nalamwipushako amepusho pali mwebene elyo ne ng'anda yenu"

**NO.**	**ICIPUSHO**	**AMASUKO**	**CILUKA**
C1	Bushe nimwebo mwe mutwe wa ino ng'anda?	YES (1)<br>NO (0)	If (1), skip to C9
C2	Bushe abene nangu umutwe wa ino ng'anda muli nabena shani?	SPOUSE (1)<br>CHILD (2)<br>GRANDCHILD (3)<br>NIECE (4)<br>AUNTIE/OTHER RELATIVE (5)<br>OTHER (SPECIFY) (6):	
C3	Mwalipitako ku sukulu?	YES (1)<br>NO (0)<br>DON'T KNOW (96)	If (0) or (96), skip to C5
C4	Mwapelela mu grade shani?<br><br>*Write grade level (ie: 03 for grade 3). If <1 year completed, write down 00. >12 years completed, write down 13.*	[ ][ ]<br><br>DON'T KNOW (96)	
C5	Lukuta nshi mulongana ko?	CATHOLIC (1)<br>PROTESTANT (2)<br>MUSLIM (3)<br>OTHER (SPECIFY) (4):	
C6	Muli mutundu nshi?		
C7	Bushe mwabashani palwa kulosha kukuupwa?	MARRIED/COHABITING (1)<br>DIVORCED (2)<br>SEPARATED (3)<br>WIDOWED (4)<br>NEVER-MARRIED (5)	If (2), (3), (4) or (5), skip to C9
C8	Nibanga pamo abakashi nagula abatemwikwa bapa mutima, abena mwenu bakwata?<br><br>*If don't know, write down 96.*	[ ][ ]	
C9	Bushe miku inga iyo mwaimitapo?		
C10	Bushe nibanga abana abo mwapapapo abatuntulu?		

INTERVIEWER: "Nomba lekeni tulande palwa ngánda yenu"

NO.	QUESTION	POTENTIAL RESPONSES	SKIP
C11	Bushe imenshi yakunwa mutapa kawi?	*PIPED WATER*<br>PIPED INTO DWELLING (1)<br>PIPED TO YARD/PLOT (2)	If (13), skip to C14

		PUBLIC TAP/STANDPIPE (3)<br>TUBE WELL OR BOREHOLE (4)<br>*DUG WELL*<br>PROTECTED WELL (5)<br>UNPROTETED WELL (6)<br>*WATER FROM SPRING*<br>PROTECTED SPRING (7)<br>UNPROTECTED SPRING (8)<br>RAINWATER (9)<br>TANKER TRUCK (10)<br>CART WITH SMALL TANK (11)<br>SURFACE WATER (12)<br>(RIVER/DAM/LAKE/POND/STREAM/CANAL/<br>IRRIGATION CHANNEL)<br>BOTTLED WATER (13)<br>OTHER (PLEASE SPECIFY) (14):	
C12	Incende mutapako amenshi nikwisa yabela?	IN OWN DWELLING (1)<br>IN OWN YARD/PLOT (2)<br>ELSEWHERE (3)	If (1) or (2), skip to C14
C13	Ciposa inshita ilishani ukufika apo mutapa amenshi no kubwela?	MINUTES (1):<br>DON'T KNOW (96)	
C14	Mulacitapo cimo pa kutila amenshi yabe bwino epo tamula nwa?	YES (1)<br>NO (0)<br>DON'T KNOW (96)	
C15	Ilingi line cimbusu ca musango shani ico mu bomfya pali ino ngánda	*FLUSH OR POUR FLUSH TOILET*<br>FLUSH TO PIPED SEWER SYSTEM (1)<br>FLUSH TO SEPTIC TANK (2)<br>FLUSH TO PIT LATRINE (3)<br>FLUSH TO SOMEWHERE ELSE (4)<br>FLUSH, DON'T KNOW WHERE (5)<br>*PIT LATRINE*<br>VENTILATED IMPROVED PIT LATRINE (6)<br>PIT LATRINE WITH SLAB (7)<br>PIT LATRINE WITHOUT SLAB/OPEN PIT (8)<br>COMPOSTING TOILET (9)<br>BUCKET TOILET (10)<br>HANGING TOILET/HANGING LATRINE (11)<br>NO FACILITY/BUSH/FIELD (12)<br>OTHER (SPECIFY) (13):	
C16	Mulabomfya icimbusu ne ndupwa shimbi?	YES (1)<br>NO (0)	

C17	Bushe ingánda yenu naikwa ifili pamo ngefi (item must be functioning usually):		YES (1)	NO (0)	DON'T KNOW (96)
	A	AMALAITI	☐	☐	☐
	B	AMALAITI YAKU KASUBA	☐	☐	☐
	C	GENERATOR	☐	☐	☐
	D	ILAMPI LYA PALAFINI	☐	☐	☐
	E	AKABATI KAKUTALIKA NOKUKOSHA	☐	☐	☐

	IFYAKULYA NANGU IFYA KUNWA (FULIJI)?			
F	MAIKULOWEFU	☐	☐	☐
G	ICITOFU CAMALASHA	☐	☐	☐
H	ICITOFU CANKUNI	☐	☐	☐
I	ICITOFU CAMALAITI	☐	☐	☐
J	UMUSENGELE	☐	☐	☐
K	MATELESHI	☐	☐	☐
L	ICIPUNA	☐	☐	☐
M	ITEBULO	☐	☐	☐
N	UMWAKUSUNGILA IMBALE NAFIMBI	☐	☐	☐
O	ICIPUNA ICANAKA ICABILILWA KUNSE	☐	☐	☐
P	INKOLOKO	☐	☐	☐
Q	ICAKU PUPA UMWELA	☐	☐	☐
R	MASHINI YA KU BILILAKO	☐	☐	☐
S	ISUMBU LYA BA MUN'GWINGWI	☐	☐	☐
T	INTERNET	☐	☐	☐
U	INKOLOKO YAPAKUBOKO	☐	☐	☐
V	AKAUNTI KU BANKI	☐	☐	☐
W	ULUKASU LU BOMFIWA NE NG'OMBE	☐	☐	☐
X	WILIBALA	☐	☐	☐
Y	ICAKU PELELAKO IFYA NSEKE-NSEKE?	☐	☐	☐
Z	MASEMBE YAKULIMINA	☐	☐	☐
AA	CHIKAYO	☐	☐	☐
BB	FOSHOLO	☐	☐	☐
CC	PANGA	☐	☐	☐
DD	PIKI	☐	☐	☐
EE	PAMPU YA MENSHI	☐	☐	☐
FF	IMPANGA YAKULIMAMO	☐	☐	☐
GG	IFIMUTI IFIKWATA IFITWALO	☐	☐	☐
HH	ICILIMBA	☐	☐	☐

II	UBUNKOLANYA	☐	☐	☐
JJ	LAMYA YAKU MINWE NANGULA IYA KWENDA NAYO	☐	☐	☐
KK	LAMYA YAMU NG'ANDA	☐	☐	☐
LL	COMPUTER	☐	☐	☐
MM	ICILIMBA CA KU LISHISHAPO AMA TEPU	☐	☐	☐
NN	ISELETI	☐	☐	☐
OO	INCHINGA	☐	☐	☐
PP	ICITUKUTUKU	☐	☐	☐
QQ	ICIKOTIKALA	☐	☐	☐
RR	MOTOKA ATEMWA ICIMBAYAMBAYA	☐	☐	☐
SS	UBWATO BWA MASHINI	☐	☐	☐
TT	UBWATO BWAKOBA	☐	☐	☐

C18	Ilingi line mulilo washani mubomfya ukwipikila pano pa ng'anda?	ELECTRICITY (1)<br>SOLAR POWER (2)<br>LIQUID PROPANE GAS (LPG) (3)<br>NATURAL GAS (4)<br>BIOGAS (5)<br>KEROSENE (6)<br>COAL, LIGNITE (7)<br>CHARCOAL (8)<br>WOOD (9)<br>STRAW/SHRUBS/GRASS (10)<br>AGRICULTURAL CROP (11)<br>ANIMAL DUNG (12)<br>NO FOOD COOKED IN HOUSEHOLD (13)<br>OTHER (SPECIFY) (14):	If (13), skip to C20
C19	Ilingi line, nikwisa mwipikila pali ino ng'anda?	IN THE HOUSE (1)<br>IN A SEPARATE BUILDING (2)<br>OUTDOORS (3)<br>OTHER (SPECIFY) (4):	
C20	Bushe fintunshi babomfeshe ukupangila panshi/apakunyanta muli iying'anda?<br><br>*OBSERVE THE FLOOR TO CONFIRM. (If more than one material, select the one that is "most" common)*	<u>NATURAL FLOOR</u><br>EARTH/SAND (1)<br>DUNG (2)<br><u>RUDIMENTARY FLOOR</u><br>WOOD PLANKS (3)<br>PALM/BAMBOO/REEDS (4)<br><u>FINISHED FLOOR</u><br>PARQUET/POLISHED WOOD (5)<br>VINYL (PVC) OR ASPHALT STRIPS (6)<br>CERAMIC/TERRAZZO TILES (7)<br>CONCRETE CEMENT (8)<br>CARPET (9)<br>OTHER (SPECIFY) (10):	

C21	Bushe umutenge wa ng'anda ino wapangilwa nenshi?<br><br>*OBSERVE THE ROOF TO CONFIRM.*<br>*(If more than one material, select the one that is "most" common)*	*NATURAL ROOFING*<br>NO ROOF (0)<br>THATCH/PALM LEAF (1)<br>*RUDIMENTARY ROOFING*<br>RUSTIC MAT (2)<br>PALM/BAMBOO (3)<br>WOOD PLANKS (4)<br>CARDBOARD (5)<br>*FINISHED ROOFING*<br>METAL/IRON SHEETS (6)<br>WOOD (7)<br>CALAMINE/CEMENT FIBRE (ASBESTOS) (8)<br>CERAMIC/HARVEY TILES (9)<br>CEMENT (10)<br>ROOFING SHINGLES (11)<br>MUD TILES (12)<br>OTHER (SPECIFY) (13):	
C22	Bushe fintunshi babomfeshe ukupangila icibumba cang'anda kunse?<br><br>*OBSERVE THE WALLS TO CONFIRM*<br>*(If more than one material, select the one that is "most" common)*	*NATURAL WALLS*<br>NO WALLS (0)<br>CANE/PALM/TRUNKS (1)<br>MUD (2)<br>*RUDIMENTARY WALLS*<br>BAMBOO/POLE WITH MUD (3)<br>STONE WITH MUD (4)<br>PLYWOOD (5)<br>CARDBOARD (6)<br>REUSED WOOD (7)<br>*FINISHED WALLS*<br>CEMENT (8)<br>STONE WITH LIME/CEMENT (9)<br>BRICK (10)<br>CEMENT BLOCKS (11)<br>WOOD PLANKS (12)<br>OTHER (SPECIFY) (13):	
C23	Bushe pa bekala muli ino ng'anda paliba abakwata impanga apa kulima?	YES (1)<br>NO (0)<br>DON'T KNOW (96)	If (0) or (96), skip to C25
C24	Bushe ma lima nama yeka ya mpanga ya kulima mo mwa kwata?	☐☐ ☐☐☐☐<br>LIMA (1) QUANTITY<br>ACRES (2)<br>HECTARES (3)<br>SQUARE METERS (4)<br>DON'T KNOW (96)	

C25	Fipendo finga ifya [INSERT ANIMAL] ino ingánda yenu yakwata?	NUMBER	NONE (00)	DON'T KNOW (96)
A	ING'OMBE SHA CIKAYA?	☐☐	☐	☐
B	ING'OMBE SHA CISUNGU ISHA MUKAKA	☐☐	☐	☐

	C	ING'OMBE SHA CISUNGU ISHA MUNANI		☐	☐
	D	BA KABALWE		☐	☐
	E	IMBUSHI		☐	☐
	F	IMBELELE		☐	☐
	G	INKUMBA		☐	☐
	H	INKOKO/ FIMBI IFITEKWA IFYAPALA IFYUNI		☐	☐
	I	UTULULU		☐	☐
	J	FIMBI IFITEKWA		☐	☐

NO.	QUESTION	POTENTIAL RESPONSES	SKIP
C26	Mukwai landeni uko mufunya indalama libili libili mumwaka wapiti.<br><br>*Select all that apply.*	SALARIED EMPLOYMENT (1)<br>SMALL BUSINESS, SHOP OR KIOSK (2)<br>SMALL HOUSEHOLD INCOME GENERATING ACTIVITY (3)<br>DOWRY (4)<br>SALE OF CROPS/ANIMALS (5)<br>SALE OF ASSETS (6)<br>REMITTANCES (CASH DONATIONS FROM FRIENDS/FAMILY) (7)<br>GOVERNMENT/NGO AID, GRANT OR OTHER FINANCIAL SUPPORT (8)<br>CASUAL DAILY WORK (9)<br>OTHER (SPECIFY) (10):	
C27	Ngacakuti pano pang'anda mwalefwaya ukukongola indalama ukufuma ku banki nangula kutwampani utukongwesha indalama (ukufumishako balupwa ne fibusa) bushe abekashi bapali ino ng'anda kuti bakwanisha?	NO (0)<br>PROBABLY NOT (1)<br>PROBABLY YES (2)<br>DEFINITELY YES (3)<br>DON'T KNOW (96)	
C28	Bushe bonse bana mayo nabana abali ne myaka isano no kwisa panshi balele mwi sumbu lyaba n'gwi n'gwi ubushiku bwafumineko?	YES (1)<br>NO (0)<br>DON'T KNOW (96)	
C29	Bushe muno ng'anda, mulakwanisha ukulipilila abana benu kusukulu ukubikapofye nafimbi ififwaikwa kumasukulu?	YES (1)<br>USUALLY (2)<br>SOMETIMES (3)<br>RARELY (4)<br>OTHER (SPECIFY) (5):<br>NO (0)	

C30	Mumwenshi wapiti, bushe kwaliko umuntu nangu umo mung'anda yenu uwaikele akasuba konse nobushiku ukwabula ukulya nangu cimo?	YES (1)<br>NO (0)<br>DON'T KNOW (96)	
C31	Bushe palipo umwana nangu umo muno ng'anda uwasendeme nensala mailo?	YES (1)<br>NO (0)<br>DON'T KNOW (96)	
C32	Bushe in'ganda yenu teti yonaike ku cimwela nagula imfula yamaka?	YES (1)<br>NO (0)<br>DON'T KNOW (96)	
C33	Ngakwaloka infula, bushe amenshi yalatala yakonkolokela kuncende kulya ukusendamina abana mung'anda?	YES (1)<br>NO (0)<br>DON'T KNOW (96)	

MODULE D. LAST DELIVERY/MOTHERS'SHELTER

INTERVIEWER: *"Nomba nalamwipushako amepusho pa kupaapa ukomwakwete nombaline. Pakubala tontonkanye pa kupaapa ukomwekwete nombaline. Elyo panuma twalaisa landapo pa muputule atemwa incenda umwika banacifyashi nga baya mukapaapa ku cipatala. Namuipekanya?"*

NO.	QUESTION	POTENTIAL RESPONSES	SKIP
D1	Bushiku nshi, umweshi no mwaka mwapaapile no mbaline? (DD MONTH YYYY)<br><br>*If date not know, ask for under 5 card. If no under 5 card, write 15th*	____ ____ ____ ____ ____ ____ ____ ____<br>D D M M Y Y Y Y	
D2	Munshita shafumako, bushe mwalyumfwako palwa mu putule atemwa insaka umwikala bana cifyashi?	YES (1)<br>NO (0)<br>DON'T KNOW (96)	If (0) or (96), skip to D18
D3	Bushe nikwisa/kulibanani mwaumfwile palwa fisaka fya banamayo?<br><br>*(Select all that apply)*	CHIEF (1)<br>HEADMEN (2)<br>HEALTH CARE WORKER (3)<br>SMAG (4)<br>TRADITIONAL BIRTH ATTENDANT (5)<br>FAMILY MEMBER (6)<br>ANOTHER MOTHER (7)<br>OTHER COMMUNITY MEMBER (8)<br>RADIO (9)<br>OTHER (SPECIFY) (10):	
D4	Ukutontonkanya pa kupaapa, bushe mwalikeleko ku nsaka ikalamo bana cifyashi epo	YES (1)<br>NO (0)	If (1), skip to D5

	tabalapaapa atemwa pa numa yakupapa?		
D4a	If NO, why?<br><br>*Select all that apply.*	NO MOTHERS SHELTER (1)<br>NO PERMISSION FROM HUSBAND OR FAMILY (2)<br>NO MONEY (3)<br>POOR QUALITY (4)<br>NOT CLEAN (5)<br>TOO CROWDED (6)<br>NOT CULTURALLY APPROPRIATE (7)<br>NOT SAFE (8)<br>DELAYS DELIVERY (10)<br>DIDN'T KNOW ABOUT MOTHERS SHELTER (11)<br>OTHER (12)<br>IF OTHER, SPECIFY	Skip to D18

INSTRUCTIONS: Ask the respondent for what reason(s) did she stay at a mothers' shelter, and then prompt her with the reasons listed below.

D5	Ni nshiku shinga mwaikele ku muputule atemwa insaka yabana cifyashi?		NUMBER OF NIGHTS	NONE (0)	DON'T KNOW (96)
	A	UBUSHIKU BWAKU CIPUMO UBWAKUBALILAPO		☐	☐
	B	SHIMBI INSHIKU SHACIPUMO		☐	☐
	C	ELYO MWALELOLELA UKUPAAPA		☐	☐
	D	ELYO BAMUFUNISHE MUCIPATALA NANGULA ELYO MWAPAPILEFYE		☐	☐
	E	INSHIKU SHITATU ISHA KUYA KUCIPIMO PANUMA YAKUPAPA		☐	☐
	F	INSHIKU CINE LUBALI UKUFIKA NA IKUMI NA CINE LUBALI ISHAKUYA KU CIPIMO PANUMA YA KUPAPA		☐	☐
	G	IMILUNGU MUTANDA IYA KUYA KU CIPIMO PANUMA YA KUPAPA		☐	☐
	H	FIMBI LUMBULENI		☐	☐

**NO.**	QUESTION	POTENTIAL RESPONSES	SKIP
D6	Cisakanshi cabana cifyashi epo mwaikalishe?	CHOMA DISTRICT<br>CHOMA GENERAL (801001)<br>MANGUNZA (801019)<br>MACHA MISSION (801002)	

	Confirm the longest number of nights the respondent stayed at a mothers' shelter.	MASUKU MISSION (801021)<br>MBABALA (801022)<br>MOCHIPAPA (801023)<br>SIMAKUTU (801043)<br>KALOMO DISTRICT<br>CHIFUSA HC (804023)<br>CHILALA HC (804024)<br>DIMBWE HC (804019)<br>HABULILE HC (804032)<br>KALOMO DISTRICT HOSPITAL (804002)<br>KANCHELE HC (804014)<br>MAWAYA HC (804034)<br>MOONDE HP (804042)<br>MUKWELA HC (804020)<br>SIACHITEMA HC (804013)<br>PEMBA DISTRICT<br>JEMBO (801413)<br>MUZOKA (801419)<br>NYIMBA DISTRICT<br>CHIPEMBE RHC (307010)<br>HOFMEYR ZONAL HC (307011)<br>KACHOLOLA RHC (307012)<br>MKOPEKA RHC (307016)<br>NYIMBA DISTRICT HOSPITAL (307001)<br>MANSA DISTRICT<br>FIMPULU (403017)<br>KABUNDA (403018)<br>LUBENDE (403041)<br>MANO (403026)<br>MANSA GENERAL HOSPITAL (403001)<br>MIBENGE (403029)<br>MUSAILA (403030)<br>MUTITI (403031)<br>MUWANGUNI (403032)<br>CHEMBE DISTRICT<br>KUNDAMFUMU (403023)<br>LUKOLA (403037)<br>LUNDAZI DISTRICT<br>CHIKOMENI (405026)<br>KAMSARO (305034)<br>KAPICHILA (305023)<br>LUKWISIZI (305040)<br>LUNDAZI HOSPITAL (305032)<br>LUSUNTHA (305021)<br>MWASE LUNDAZI ZONAL (305011)<br>NKHANGA (305046)<br>NYANGWE (305020)<br>PHIKAMALAZA (305031)<br>ZUMWANDA (305024)<br>OTHER (SPECIFY NAME OF HEALTH FACILITY AND DISTRICT) (47):	

INTERVIEWER: *"Nomba nalamwipushako amepusho pafyo mwaikele pansaka yaba cifyashi. Kwateni akashita kanoono nokutontonka ifyo mwaikele. Namuipekanya ukuti tutampeko?"*

SURVEY ID

D7	Elyo mwaikele pa nsaka ya bana cifyashi---	YES (1)	NO (0)	DON'T KNOW (96)
A	BUSHE KWAILI UBUSANSHI NAGULA MATELESHI EPOMWALELALA?	☐	☐	☐
B	BUSHE MWALEBOMFYA UBUSANSHI ATEMWA MATELESHI NA BANTU BAMBI?	☐	☐	☐
C	BUSHE MWALILEPO MWISUMBU LYABA N'GWI N'GWI?	☐	☐	☐
D	BUSHE BALIMILONDOLWELA AMAFUNDE NEFYAKUKONKA KU CISAKA CABANAYO ILYO MWAFIKILE?	☐	☐	☐
E	BUSHE MWALEBOMFYWA AMENSHI AYASHAKWATA IFIKO ETEMWA AYA SUSWA BWINO?	☐	☐	☐
F	BUSHE MWALEKWATA ICENGELO (AMALAITI) AKASUBA NGA KAWA?	☐	☐	☐
G	BUSHE MWALIKWETE UKWA KU SAMBILA NOKWA KU CAPILA?	☐	☐	☐
H	BUSHE KWALI INCENDE IYABENDAMA UKWAKUBIKA IFIPE FYENU NE FYA KULYA?	☐	☐	☐
I	BUSHE MWALIKWETEPO AMASAMBILISHO AYA BUMI?	☐	☐	☐

NO.	QUESTION	POTENTIAL RESPONSES	SKIP
D8	Bushe pali apakwipikila mu cisaka cabana cifyashi?	YES (1)<br>NO (0)<br>DON'T KNOW (96)	If (0) or (96), skip to D10
D9	Bushe iyi ncende yakwipikilamo yali iyafimbwa?	YES (1)<br>NO (0)<br>DON'T KNOW (96)	
D10	Bushe mwalisambiliko imikalile iyipya elyo mwali pa nsaka atemwa umuputule wabana cifyashi?	YES (1)<br>NO (0)<br>DON'T KNOW (96)	If (0) or (96), skip to D12
D11	Mikalilenshi mwa sambilileko?		
D12	Bushe balimyeba ukusonka indalama pakwikala ku cisaka ca banamayo?	YES (1)<br>NO (0)<br>DON'T KNOW (96)	If (0) or (96), skip to D14
D13	Nishinga indalama mwalipile shonse pamo?		

SURVEY ID | | | | | | |

D14	Bushe balimyebele ukusonka ifintu fimbi ukufumishako indalama pakwikala ku cisaka ca banamayo?	YES (1)<br>NO (0)<br>DON'T KNOW (96)	If (0) or (96), skip to D16
D15	Finshi mwasonkele?<br><br>*(Select all that apply)*	LABOR (1)<br>LIVESTOCK/POULTRY (2)<br>FOOD OR OTHER AGRICULTURAL RESOURCES (3)<br>OTHER IN-KIND RESOURCES (SPECIFY) (4):<br>OTHER (SPECIFY) (5):	

D16	***INTERVIEWER:*** *"Palikano kashita, nalamwipushako pamafya ayasangwa-sangwa kuli banamayo ilyo baleikala ku fisaka fya banamayo libe tabalapapa. Nganalumbula bumo ubwafya, ndemulomba munjebeko ngacakuti mwalishingwana nabo ilyo mwaleikalako, ukubikilakofye ngacakuti bwali ubwafya ubukalamba nangu ubunono kuli imwe."*					
			MAJOR PROBLEM (2)	MINOR PROBLEM (1)	NO PROBLEM (0)	UNDECIDED (96)
	A	UMUSANGO CAPANGILWAMO	☐	☐	☐	☐
	B	IMISUNGILE NE FYALESHUPA	☐	☐	☐	☐
	C	UBUSAKA	☐	☐	☐	☐
	D	UKUSANGWA KWA BABOMFI	☐	☐	☐	☐
	E	UKUSANSAMUKA KWA BABOMFI	☐	☐	☐	☐
	F	UKUBOMFYA INCENDE YAKWIPIKILA	☐	☐	☐	☐
	G	UKUFULISHA KWA BANTU	☐	☐	☐	☐
	H	IFYAKUCINGILILA	☐	☐	☐	☐
	I	ICITENDWE	☐	☐	☐	☐
	J	UKULINGANA MU NTAMBI	☐	☐	☐	☐

NO.	QUESTION	POTENTIAL RESPONSES	SKIP
D17	Bushe kuti mwatila mwalisekelamo pamikalile ya pa nsaka ya bana cifyashi?	VERY SATISFIED (1)<br>MORE OR LESS SATISFIED (2)<br>NOT SATISFIED (3)	
D17a	Do you intend to use mothers' shelter for future deliveries?	YES (1)<br>NO (0)<br>DON'T KNOW (96)	
D17b	Would you recommend using a mothers' shelter to friends or relatives?	YES (1)<br>NO (0)<br>DON'T KNOW (96)	

SURVEY ID | | | | | | |

INTERVIEWER: *"Natasha saana pa kwasuka amepusho pa mulandu wafisaka kulya kwikala bana cifyashi nga baya mu ku paapa"*

D18	Nibanani bamwafwilisheko pakupapa pamuku wakulekelesha?<br><br>*(Select all that apply)*<br><br>*If respondent says NO ONE ASSSISTED, probe to determine whether any adults were present at the delivery.*	DOCTOR/CLINICAL OFFICER (1)<br>NURSE/MIDWIFE (2)<br>OTHER HEALTH FACILITY STAFF/PERSONNEL (3)<br>TRADITIONAL BIRTH ATTENDANT (4)<br>SMAG (5)<br>RELATIVE/FRIEND/AUNTIE (6)<br>NO ONE ASSISTED (7)<br>OTHER (SPECIFY) (8):	
D18 a	Where do you intend to deliver your next baby?	YOUR HOME (1)<br>OTHER HOME (2)<br>HEALTH POST/FACILITY (3)<br>HOSPITAL (4)<br>OTHER (SPECIFY) (5):	
D18 b	Where did you intend to deliver your last delivery?	YOUR HOME (1)<br>OTHER HOME (2)<br>HEALTH POST/FACILITY (3)<br>HOSPITAL (4)<br>OTHER (SPECIFY) (5):	
D19	Nikwisa mwapapile umwana wenu uwapakulekelesha?	YOUR HOME (1)<br>OTHER HOME (2)<br>HEALTH POST/FACILITY (3)<br>HOSPITAL (4)<br>OTHER (SPECIFY) (5):	If (1), (2) or (5), skip to D31
**INSTRUCTIONS:** If respondent answers **OTHER (5) to Question D19**, probe to ensure this is not a health post/facility or hospital.			

FACILITY-BASED DELIVERY			
**NO.**	QUESTION	POTENTIAL RESPONSES	SKIP
D20	Niku chipala nshi **mwabalilepo** ukuya pakupapa kwenu ukwakulekelesha?<br><br>*(**INSTRUCTIONS:** if woman reports hospital, probe to ensure she did not first present at a health facility and was transferred to hospital.)*	CHOMA DISTRICT<br>CHOMA GENERAL (801001)<br>MANGUNZA (801019)<br>MACHA MISSION (801002)<br>MASUKU MISSION (801021)<br>MBABALA (801022)<br>MOCHIPAPA (801023)<br>SIMAKUTU (801043)<br>KALOMO DISTRICT<br>CHIFUSA HC (804023)<br>CHILALA HC (804024)<br>DIMBWE HC (804019)<br>HABULILE HC (804032)<br>KALOMO DISTRICT HOSPITAL (804002)<br>KANCHELE HC (804014)<br>MAWAYA HC (804034)<br>MOONDE HP (804042)<br>MUKWELA HC (804020)<br>SIACHITEMA HC (804013)<br>PEMBA DISTRICT<br>JEMBO (801413)	

		MUZOKA (801419)<br>NYIMBA DISTRICT<br>CHIPEMBE RHC (307010)<br>HOFMEYR ZONAL HC (307011)<br>KACHOLOLA RHC (307012)<br>MKOPEKA RHC (307016)<br>NYIMBA DISTRICT HOSPITAL (307001)<br>MANSA DISTRICT<br>FIMPULU (403017)<br>KABUNDA (403018)<br>LUBENDE (403041)<br>MANO (403026)<br>MANSA GENERAL HOSPITAL (403001)<br>MIBENGE (403029)<br>MUSAILA (403030)<br>MUTITI (403031)<br>MUWANGUNI (403032)<br>CHEMBE DISTRICT<br>KUNDAMFUMU (403023)<br>LUKOLA (403037)<br>LUNDAZI DISTRICT<br>CHIKOMENI (405026)<br>KAMSARO (305034)<br>KAPICHILA (305023)<br>LUKWISIZI (305040)<br>LUNDAZI HOSPITAL (305032)<br>LUSUNTHA (305021)<br>MWASE LUNDAZI ZONAL (305011)<br>NKHANGA (305046)<br>NYANGWE (305020)<br>PHIKAMALAZA (305031)<br>ZUMWANDA (305024)<br>OTHER (SPECIFY NAME OF HEALTH FACILITY AND DISTRICT) (47):	
D21	Ninshilanshi mwabomfeshe ukwenda pakuya fika ku chipatala pakupapa kwenu ukwakulekelesha?	WALKING (1)<br>BICYCLE (2)<br>CARRIED IN WHEELBARROW (3)<br>ANIMAL-DRAWN CART (4)<br>TAXI (5)<br>CAR (6)<br>MOTORCYCLE (7)<br>AMBULANCE (8)<br>OTHER (SPECIFY) (9):	
D22	Ukubomya talanshipoti mubomfya ukuya ku cipatala, caposele inshita ilishani pukuti mufike ku chipatala?<br><br>*Be sure to specify unit of response.*	[ ][ ] HOURS [ ][ ] MINUTES	
D23	Bushe mwapapile umwana wenu ku chipatala ico mwaileko pakubala?	YES (1)<br>NO (0)<br>DON'T KNOW (96)	If (1) or (96) skip to D27
D24	Lishina nshi lya chipatala uko mwapaapile umwana?	CHOMA DISTRICT<br>CHOMA GENERAL (801001)	

		MANGUNZA (801019)<br>MACHA MISSION (801002)<br>MASUKU MISSION (801021)<br>MBABALA (801022)<br>MOCHIPAPA (801023)<br>SIMAKUTU (801043)<br>KALOMO DISTRICT<br>CHIFUSA HC (804023)<br>CHILALA HC (804024)<br>DIMBWE HC (804019)<br>HABULILE HC (804032)<br>KALOMO DISTRICT HOSPITAL (804002)<br>KANCHELE HC (804014)<br>MAWAYA HC (804034)<br>MOONDE HP (804042)<br>MUKWELA HC (804020)<br>SIACHITEMA HC (804013)<br>PEMBA DISTRICT<br>JEMBO (801413)<br>MUZOKA (801419)<br>NYIMBA DISTRICT<br>CHIPEMBE RHC (307010)<br>HOFMEYR ZONAL HC (307011)<br>KACHOLOLA RHC (307012)<br>MKOPEKA RHC (307016)<br>NYIMBA DISTRICT HOSPITAL (307001)<br>MANSA DISTRICT<br>FIMPULU (403017)<br>KABUNDA (403018)<br>LUBENDE (403041)<br>MANO (403026)<br>MANSA GENERAL HOSPITAL (403001)<br>MIBENGE (403029)<br>MUSAILA (403030)<br>MUTITI (403031)<br>MUWANGUNI (403032)<br>CHEMBE DISTRICT<br>KUNDAMFUMU (403023)<br>LUKOLA (403037)<br>LUNDAZI DISTRICT<br>CHIKOMENI (405026)<br>KAMSARO (305034)<br>KAPICHILA (305023)<br>LUKWISIZI (305040)<br>LUNDAZI HOSPITAL (305032)<br>LUSUNTHA (305021)<br>MWASE LUNDAZI ZONAL (305011)<br>NKHANGA (305046)<br>NYANGWE (305020)<br>PHIKAMALAZA (305031)<br>ZUMWANDA (305024)<br>OTHER (SPECIFY NAME OF HEALTH FACILITY AND DISTRICT) (47):	
D25	Bushe uku kuchipatala bamituminekofye kubabomfi abangalila ubumi?	YES (1)<br>NO (0)<br>DON'T KNOW (96)	If (0) or (96), skip to D27

SURVEY ID

D26	Elenganyenyi inshita yapitilepo pakuti bafumya nokumutwala kuli ici chipatala?	LESS THAN 1 HOUR (1)<br>1 TO 2 HOURS (2)<br>MORE THAN 2 HOURS (3)	
D27	Nibanani abene-bene abapingwile ukuti mukapapile ku chipala?	YOURSELF (1)<br>HUSBAND/PARTNER (2)<br>MOTHER/MOTHER-IN-LAW (3)<br>AUNTIE (4)<br>OTHER FAMILY MEMBER (5)<br>FRIEND (6)<br>OTHER (SPECIFY) (7):	
D28	Bushe mwalikele pa chipatala ukufika napa nsa amakumi yabili na cine elyo baisa mufunya?	YES (1)<br>NO (0)<br>DON'T KNOW (96)	

D29	***INTERVIEWER:*** *"*Nalalumbulako ifintu ifingi ifyo bapela pakwafwilishako abantu ku chipatala, elo ndekabila ukwishiba ngacakuti mwalipokelelako nangu iyo kucipatala ilyo mwaile mukupapa.*"*			
		RECEIVED (1)	DID NOT RECEIVE (0)	DON'T KNOW (96)
	A Ukupapa kwenu ukwakulekelesha, bushe balimilepula? Ici cilepilibula mukweba ati bamilepula pamala elo bafumyamo umwana.	☐	☐	☐
	B Ukupapa kwenu ukwakulekelesha, bushe balimibikako umulopa?	☐	☐	☐
	C umuti/ukubikwa amenshi mumushipa	☐	☐	☐
	D Ukusambilishanya palwa konsha	☐	☐	☐
	E Ifyakutalukanya abana nayambi amasambilisho	☐	☐	☐
	F Ukwishiba ifya kubika Umwana pa lufumo nga kangalu pakuti akwate icikabilila	☐	☐	☐

D30	***INTERVIEWER:*** *"*Palikano kashita, nalamwipushako pamafya ayasangwa-sangwa kuli banamayo ilyo baleikala ku fisaka fya banamayo libe tabalapaapa. Nganalumbula bumo ubwafya, ndemulomba munjebeko ngacakuti mwalishingwana nabo ilyo mwaleikalako, ukubikilakofye ngacakuti bwali ubwafya ubukalamba nangu ubunono kuli imwe.*"*				
		MAJOR PROBLEM (2)	MINOR PROBLEM (1)	NO PROBLEM (0)	UNDECIDED (96)
	A IMIBELE YAMITI BAPELA PAKUPAPA	☐	☐	☐	☐
	B UMUCINSHI UPELWA NABA BOMFI BAMONA PA BUMI	☐	☐	☐	☐
	C UKUBA UKWAFISAMA PA KUPAPA	☐	☐	☐	☐
	D UBUSAKA BWA CIPATALA	☐	☐	☐	☐
					**After completing the facility-based delivery section, continue to MODULE E.**

HOME DELIVERIES

No.	Question	Potential responses	Skip
D31	Nibanani abene-bene abapingwile ukuti mukapapile ku ng'anda?	YOURSELF (1)<br>HUSBAND/PARTNER (2)<br>MOTHER/MOTHER-IN-LAW (3)<br>AUNTIE (4)<br>OTHER FAMILY MEMBER (5)<br>FRIEND (6)<br>OTHER (SPECIFY) (7):	
D32	Cinshi icikalamba icalengele ukuti mwipaapila ku chipatala?<br><br>*Select all that apply.*	COST TOO MUCH (1)<br>FACILITY NOT OPEN (2)<br>TOO FAR/NO TRANSPORTATION (3)<br>POOR QUALITY SERVICE/DON'T TRUST (4)<br>NO FEMALE HEALTH PROVIDER (5)<br>HUSBAND/FAMILY DIDN'T ALLOW (6)<br>SHORT LABOR (7)<br>BABY CLOTHES (8)<br>CDK (9)<br>NO MOTHERS SHELTER (10)<br>NOT NECESSARY (11)<br>NOT CUSTOMARY (12)<br>OTHER (SPECIFY) (13):	
D33	Bushe panuma ya kupapa, mwalile ku chipatala pukuya muceceta ubumi bwenu no bwa mwana nishi tapala pita insa amakumi yabili na cine?	YES (1)<br>NO (0)<br>DON'T KNOW (96)	If 0 or 96, skip to Module E

MODULE E: SPENDING AND SAVINGS

***INTERVIEWER:** "Nomba nalaipushako amepusho pa mitengo atemwa ifyo mwaposelepo pakupaapa kwenu ukwakulekelesha. Tontonkanyeni palifyonse ifyo mwaposelepo nefyo mwaipekenye"*

E1					
	***INTERVIEWER**: "Nomba nalanda naimwe pa ndalama mwaposele pa kupaapa mwakwete ukwa kulekelesha. Tontonkanyeni pa fintu mwashitile ilyo mwalepekanya ukuya mu kupaapa, ukuya ku chipatala nangula ku ngánda uko mwapapile ne nshita mwapaapile pachipatala nangula pa ngánda. Namuipekanya twambeko? "*				
	Nishinga mwaposele pali ifi:		AMOUNT (KWACHA)	NONE (0)	DON'T KNOW (96)
	In preparation:				
	A	IFYAKUPAPILAKO (ifili nga amagolofu, ifyakupukutilako, utulesa, ifipulashitiki, imiti yakwipaya utushishi nafimbi po)			
	B	IFYAKUFWALA FYA MWANA NO BULENGETI			
	On your journey:				
	C	TALANSHIPOTI UKUYA NO KUBWELA (Ngani pa ng'anda bikenipo ilyakwa namungwa)			

SURVEY ID

	D	AMILALILE PANSAKA YABANA CIFYASHI NAKUMBI UKWAKUSENDAMA ELYO MWALELOLELA UKUPAAPA			
	***At time of delivery*:**				
	E	INDALA MA ICHIPATALA CILIPILISHA			
	F	AMALIPILO YAMO AYASHILI AYA TANTIKWA			
	G	IFYA KU CINKULANA			
	H	IFISHILI TELUPIYA (ELENGANYENI UMUTENGO WAFIKO)			
	I	IMITI			
	J	IFYA KUPIMA PIMA			
	K	NAFIMBI IFYO MWALIPILEPO INDALAMA			

NO.	QUESTION	POTENTIAL RESPONSES	SKIP
E2	Bushe mwalikweteko indalama isho mwasungilefye mukuteyanya ukupapa kwenu ukwakulekelesha?	YES (1)<br>NO (0)<br>DON'T KNOW (96)	If (0) or (96), skip to E8
E3	Bushe mulemona kwati mwalisunga indalama ishingalinga ukupapa kwenu ukwakulekelesha?	YES (1)<br>NO (0)<br>DON'T KNOW (96)	
E4	Nikwisa mwabikile indalama isho mwasungile?	AT YOUR HOME (1)<br>AT A FRIEND OR FAMILY MEMBER'S HOME (2)<br>IN A BANK ACCOUNT (3)<br>OTHER (SPECIFY) (4):	
E5	Bushe kwali umuntu uli onse mung'anda yenu (limbi abena myenu) abaiketeko ku ndalama mwasungile?	YES (1)<br>NO (0)<br>DON'T KNOW (96)	
E6	Ifumo lyali ne myeshi inga ilyo mwatendeke ukusunga ulu ulupiya?	WEEKS<br>MONTHS	
E7	Nibani bambi abasangwileko ulupiya lwakupapila (mukupekanya, mu lwendo, pa nshita yakupapa)?<br><br>*(Select all that apply)*	HUSBAND/PARTNER (1)<br>YOUR CHILDREN (2)<br>PARENT/GRANDPARENT (3)<br>OTHER FAMILY MEMBER (4)<br>FRIEND (5)<br>AUNTIE (6)<br>NO ONE (7)<br>OTHER (SPECIFY) (8):	

E8	Mumitontonkanyishe yenu, bushe cacindama shani ukusunga ulupiya lwakupapila?	NOT IMPORTANT (1)<br>SLIGHTLY IMPORTANT (2)<br>MODERATELY IMPORTANT (3)<br>IMPORTANT (4)<br>VERY IMPORTANT (5)	
E9	Bushe mwalibala amusungapo indalama mucipao cakubanki?	YES (1)<br>NO (0)<br>DON'T KNOW (96)	
E10	Bushe mwalibala amutumapo indalama pali lamya?	YES (1)<br>NO (0)<br>DON'T KNOW (96)	In (0) or (96), skip to module F
E11	Bushe nibanani mwatumineko ukubomfya "mobile money"?	HUSBAND/PARTNER (1)<br>YOUR CHILDREN (2)<br>PARENT/GRANDPARENT (3)<br>OTHER FAMILY MEMBER (4)<br>FRIEND (5)<br>AUNTIE (6)<br>OTHER (SPECIFY) (7):	

MODULE F. POST-NATAL CARE

INTERVIEWER: *"Nomba kuti natemwa ukumwipushako amepusho ayanono pakutangatwa ukuli konse ukwa bumi uko imwe nomwana wenu mwapokelele panuma yakupapa kwakulekelesha."*

NO.	QUESTION	POTENTIAL RESPONSES	SKIP
F1	Bushe mwalile kuciputulwa cabumi mukucencentwa ukuli konse panuma yakupapa kwakulekelesha?	YES (1)<br>NO (0)<br>DON'T KNOW (96)	If (0) or (96) skip to F6
F2	Bushe mwalileko kuciputulwa cabumi kukumuceceta panuma yakupaapa mupepi nenshinku shitatu panuma kwakupapa kwakulekelesha?	YES (1)<br>NO (0)<br>DON'T KNOW (96)	
F3	Bushe mwalileko kuciputulwa cabumi mukucencentwa panuma yakupapa pakati kabushiku bwalenga 7 na 14 panuma yakupapa kwakulekelesha?	YES (1)<br>NO (0)<br>DON'T KNOW (96)	
F4	Bushe mwalileko kuciputulwa cabumi mupepi nemilungu 6 panuma yakupapa kwakulekelesha?	YES (1)<br>NO (0)<br>DON'T KNOW (96)	

F5	***INTERVIEWER:*** "Palikano kashita, nalamwipushako pamafya ayasangwa-sangwa kuli banamayo ilyo baya ku cipimo panuma ya kupapa. Nganalumbula bumo ubwafya, ndemulomba munjebeko ngacakuti mwalishingwana nabo ilyo mwaleikalako, ukubikilakofye ngacakuti bwali ubwafya ubukalamba nangu ubunono kuli imwe."

SURVEY ID | | | | | | |

		MAJOR PROBLEM (2)	MINOR PROBLEM (1)	NO PROBLEM (0)	UNDECIDED (96)
A	INSHITA MWALOLELE PAKUTI BAMIMONE	☐	☐	☐	☐
B	IMILANSHANISHISHE YA MAFYA NANGU IFIMISAKAMIKA PE FUMO LYENU	☐	☐	☐	☐
C	UBULONDOLOSHI MWAPOKELE PA BWAFYA NANGU UKUNDAPWA	☐	☐	☐	☐
D	UKUCINGILILWA PAKUTI BAMBI BEMONA IFYO BALEMIPIMA	☐	☐	☐	☐
E	UKUCINGILILWA PAKUTI BAMBI BEUMFWA IFYO MULELANSHANYA PAFYO MULWELE	☐	☐	☐	☐
F	UMUSAKA BWA CHIPATALA	☐	☐	☐	☐
G	IFYO ABABONFI BA BUMI BAMITANGATILEMO	☐	☐	☐	☐
H	IMILIPILILE YA KUNDAPWA	☐	☐	☐	☐

F6	Pali ndakayi, mulabomfyako inshila iliyonse iyakucelesha nangu ukulesha ukwimita?	YES, MODERN METHOD (1)<br>YES, TRADITIONAL METHOD (2)<br>NO (0)<br>N/A, CURRENTLY PREGNANT (3)<br>DON'T KNOW (96)	
F7	**INSTRUCTIONS:** Look back to question **B27** – Bushe umwana wafyelwe pakulekelesha *epoacili? Confirm with respondent.*	YES (1)<br>NO (0)<br>DON'T KNOW (96)	If (0) or (96) skip to F15
F8	Bushe mucili muleonsha akanya mwapapile umuku wakulekelesha?	YES (1)<br>NO (0)<br>DON'T KNOW (96)	If (0) or (96) skip to F10
F9	Bushe pali ndakayi mulamulishako umwana ifyakulya fimbi ukucila pa mukaka wakwi bele no muti?	YES (1)<br>NO (0)<br>DON'T KNOW (96)	
F10	Pamilungu ibili iyakulekelesha, mwalifwaileko ubwafwilisho pabumi bwa mwana wenu pamulandu uuli onse?	YES (1)<br>NO (0)<br>DON'T KNOW (96)	If (0) or (96), skip to F12
F11	Nikwisa mwaletele umwana pakubala pakusakamanwa kwa bumi?	HEALTH CARE CENTER (1)<br>HOSPITAL (2)<br>PHARMACY (3)<br>TRADITIONAL HEALER (4)<br>OTHER (SPECIFY) (5):	
F12	Bushe umwana wenu alitungwapo inyeleti ishili shonse?	YES (1)<br>NO (0)<br>DON'T KNOW (96)	If (0) or (96), skip to F15

F13	**INSTRUCTIONS:** Based on **D1**, calculate child's age.		

SURVEY ID | | | | | | |

	Specify unit of response.		
	**INSTRUCTIONS:** Ask to see the child's vaccination card. If available, use card to confirm the vaccines received and mark below. If card is unavailable, ask mother which vaccines the child has received.<br>**BASED ON CALCULATED AGE FROM F13**, ask only about **AGE APPROPRIATE** vaccines.		
F14	Confirm you have the child's vaccine card in-hand.	YES (1)<br>NO (0)	

		CONFIRMED BY VACCINE CARD		CONFIRMED BY MOTHER	
		RECEIVED	NOT RECEIVED	RECEIVED	NOT RECEIVED
	**Bushe umwana alitungilwe inyeleti ishi pakufyalwa?**				
	A BCG	☐	☐	☐	☐
	B Polio (OPV-0)	☐	☐	☐	☐
	**Bushe umwana alitungilwe inyeleti shapamilungu mutanda ishitantikwe ngefi?**				
	C Polio (OPV-1)	☐	☐	☐	☐
	D DTP-HepB-Hib-1	☐	☐	☐	☐
	E Pneumococcal (PCV)	☐	☐	☐	☐
	F Rotavirus	☐	☐	☐	☐
	**Bushe umwana alipokelele inyeleti ishi pamilungu ikumi limo na fine?**				
	G Polio (OPV-2)	☐	☐	☐	☐
	H DTP-HepB-Hib-2	☐	☐	☐	☐
	I Pneumococcal (PCV)	☐	☐	☐	☐
	J Rotavirus	☐	☐	☐	☐
	**Did your child receive the following 14-week vaccines?**				
	K Polio (OPV-3)	☐	☐	☐	☐
	L DTP-HepB-Hib-3	☐	☐	☐	☐
	M Pneumococcal (PCV)	☐	☐	☐	☐

Interviewer: *"Amepusho ayakonkelepo, mwasukefye ngacakuti mulefwaya. Yambi kuti mwapela umasuko yambi iyo."*			
F15	Mwalipimishepo HIV elyo mwali nefumo lyakulekelesha?	YES (1)<br>NO (0)<br>PREFER NOT TO ANSWER (2)<br>DON'T KNOW (96)	
F16	Imibele yenu kulyashi lyakashishi ka HIV ilishani?	INFECTED (1)<br>NOT-INFECTED (2)<br>PREFER NOT TO ANSWER (3)<br>DON'T KNOW (96)	If (2), (3), or (96), skip to F23
F17	Bushe mwalenwa umuti wama ARVs pefumo lyakulekelesha?	YES (1)<br>NO (0)<br>DON'T KNOW (96)	
F18	**INSTRUCTIONS:** Refer back to question **B27-28.** Did the respondent's baby survive beyond the day of birth?<br><br>*Confirm with respondent.*	YES (1)<br>NO (0)<br>DON'T KNOW (96)	If (0) or (96), skip to Module G
F19	Bushe umwana wenu alinwineko umuti wama ARV's	YES (1)<br>YES, BUT BABY DIED BEFORE 6 WEEKS OF AGE (2)	

	ukulingana imilungu 6 panuma yakufyalwa?	NO (0)<br>DON'T KNOW (96)	
F20	Bushe umwana alipiminwe na kashishi ka HIV?	EE (1)<br>AWE (0)<br>NSHISHIBE (96)	If (0) or (96), skip to F23
F21	Umwana wenu alinemilungu inga iyakufyalwa ilyo mwamupimishe?<br><br>*Round to nearest full number.*		
F22	Finshi fyasangilwe mucipimo ca HIV icamwana wenu?	INFECTED (1)<br>NOT INFECTED (2)<br>PREFER NOT TO ANSWER (3)<br>DON'T KNOW (96)	

F23	Panshiku shitatu ishapita, bushe pali umuntu uwikala muno ng'anda uwuli nemyaka yakufyalwa ukucila ikumi limo na isano 15 nangu imwebene abacitilepo ifi fintu nomwana?<br><br>*Select all that apply*			
		YES (1)	NO (0)	DON'T KNOW (96)
A	UKUBELENGELA UMWANA IFITABO NANGU UKULOLESHA IFIKOPE MUFITABO PAMO NOMWANA	☐	☐	☐
B	UKUSHIMIKILA UMWANA UTUSHIMI	☐	☐	☐
C	UKWIMBA/UKWIMBILA UMWANA INYMBO	☐	☐	☐
D	UKUFUMYA UMWANA PANSE YA NG'ANDA, YADI, UMUSHI NANGU UMWAFIMBIKA	☐	☐	☐
E	AMASHINA, UKUPENDA NANGU UKULENGA UTULENGO NOMWANA	☐	☐	☐

F24	***INTERVIEWER:*** *"For the following questions, please respond only if you feel comfortable doing so. Your response is optional. I am going to read you a list of problems. Please tell me how often each of these problems has happened to you in the PAST TWO WEEKS: never, once in a while, more than half the time, or almost always."*				
		NEVER (0)	ONCE IN A WHILE (1)	MORE THAN HALF THE TIME (2)	ALMOST ALWAYS (3)
A	PAMILUNGU SHIBILI/IBILI IYAPITA NSHILI UWAKUTEMWA NAGULA UWANSANSA.	☐	☐	☐	☐
B	PAMILUNGU SHIBILI/IBILI IYAPITA NSHATALA KWATAPO UKUTEMWA IFINTU, INCHITO NANGULA ABANTU.	☐	☐	☐	☐
C	PAMILUNGU SHIBILI/IBILI IYAPITA NALI UWACHILILISHI.	☐	☐	☐	☐
D	PAMILUNGU SHIBILI/IBILI IYAPITA NALI UWANKUMBABULILI, NOWAITALUSHA KU BANTU.	☐	☐	☐	☐

SURVEY ID | | | | | | |

F25	*INTERVIEWER: "Now I am going to read you a list of things that you may have experienced. Please tell me how often each of these events have happened to you in the past two weeks: never, once in a while, a few times, or many times. Again, please respond only if you feel comfortable doing so. Your response is optional."*					
			NEVER (0)	ONCE (1)	A FEW TIMES (2)	MANY TIMES (3)
	A	PAMILUNGU SHIBILI/IBILI IYAPITA MIKU INGA EYO ABENAMWENU NAGULA ABATEMWIKWA BENU NANGULA UKUMITOBA ULUPI?	☐	☐	☐	☐
	B	PAMILUNGU SHIBILI/IBILI IYAPITA MIKU INGA EYO ABENAMWENU NAGULA ABATEMWIKWA BENU BAMIPANTILEPO, UKUMIKULULA, UKUMA, UKUMIKAMA ELO UKUMYOCHA?	☐	☐	☐	☐

MODULE G. LAST PREGNANCY

INTERVIEWER: *"Nomba nalamwipushako amepusho yamo pefumo lyenu ukufika na pa kupapa kwanomba line. sendeniko akashita no kutontonkanya kunuma ilyo mwabalilepo ukwishiba ukuti muli nefumo elyo na pacipimo. Bushe mwaipekanya ukwamba?"*

NO.	QUESTION	POTENTIAL RESPONSES			SKIP
**Interviewer**: *Ask to see if antenatal care card is available for the woman's last pregnancy that led to a delivery and confirm information provided by respondent.*					
G1	Did the woman provide you with her antenatal card?	YES (1)<br>NO (0)<br>DON'T KNOW (96)			
G2	Miku inga mwatangetwe kucipimo pa ciputulwa cabumi pefumo lyakulekelesha?	NONE (0)<br>ONE TIME (1)<br>TWO TIMES (2)<br>THREE TIMES (3)<br>FOUR TIMES (4)<br>MORE THAN FOUR TIMES (5)			If (0) skip to End of Survey
G3	Bushe elyo mwaile kucipimo mwalilanshyanya palwa:	YES (1)	NO (0)	DON'T KNOW (96)	
	A Ukomukapapila umwana?	☐	☐	☐	
	B Ifyo mwingacita ngacakwebati kwabalamuka amafya pakupapa?	☐	☐	☐	
	C Ukusungisha indalama ishakubomfya libe ifumo lyabalamuka napa kupapa?	☐	☐	☐	

	D Ubushiku bwapendwa bwakupapa?	☐ ☐ ☐	
G4	Bushe muleibukisha ubushiku bwapendwa ubwakupapa ubo bamwebele?	YES (1)<br>NO (0)<br>DON'T KNOW (96)	If (0), skip to G6
G5	Bushiku nshi mwalemona ukuti kuti mwapapa? (DD MONTH YYYY)<br><br>*If EDD is on ANC card, copy it from card. If no card and date not know, enter 15th*	____ ____ ____ ____ ____ ____ ____ ____<br>D D M M Y Y Y Y	
G6	Ifumo lyali imilungu inga nelyo imyeshi inga ilyo mwaile pakubalilapo kucipimo?<br><br>*Please specify unit of response.*	WEEKS<br><br>MONTHS	

INTERVIEWER: *"Twafika ku mpela ya kufwailikisha uku. Namutasha apakalamba nganshi pa nshita mwaposa pa kwasuka amepusho.*

G7	Would you be willing to have someone come back and follow up on a few questions from the survey in the next couple of days?	YES (1)<br>NO (0)	

INTERVIEWER: " *Namutasha apakalamba. Namukwata ifyo mwingafwaya ukulunda po?"*

COMMENTS:

SURVEY ID | | | | | | |

END OF SURVEY

INSTRUMENT REVIEW

Enumerator Initials:	
Date (DD/MM/YYYY)	
Supervisor Initials:	
Date (DD/MM/YYYY)	

Data Entry Initials:	
Date (DD/MM/YYYY)	
Supervisor Initials:	
Date (DD/MM/YYYY)	
