# Supplementary material for: Characteristics of maternity waiting homes and the women who use them: Findings from a baseline cross-sectional household survey among SMGL-supported districts in Zambia
Source: PLoS One. 2018 Dec 31;13(12):e0209815. doi: 10.1371/journal.pone.0209815 (PMC6312364; doi:10.1371/journal.pone.0209815)
Supplement: S4 File — Household Survey Baseline Impact Evaluation–Tumbuka. (PDF) [file pone.0209815.s004.pdf]


SURVEY ID

Instrument ID:

The MAHMAZ Project

Baseline Impact Evaluation – Household Survey TUMBUKA

Target Audience:

Women who have delivered a child in the last 12 months, who are ≥ 15 years of age, and who live within the study facility catchment areas

SHORT SCREEN

SS1	Kasi mbalinga banakazi abo bali navilimika vyakubabika kuyambira pa 15 kuflka pa 49 abo bakukhara pankhomo linu, kusazgirako na abo balikutayika mukuti mucilimika ici cajumpha?<br><br>*Write down the number of women.*		If none, thank person and move to next household.
SS2	Kasi walipo yumoza panakazi abo uyo waka phokwa mumiyezi 12 yajumphapo (chaka chimoza) kumasinda uku, kwambura kusazgirako ivo vikacitika ku umoyo wa mwana uyo wakabambika panji ku umoyo wa banyina abo baka phokwa?	Yes (1)<br>No (0)<br>Don't know (96)	If (0) or (96), thank person and move to next household
SS3	Para mwanakazi uyu parije panyengo yino yason, kasi mungamuzgorerako mafupo ayo yakukwaska nap kati apo wakaba napo na umo wakababila?	Yes (1)<br>No (0)<br>Don't know (96)	
**INTERVIEWER: IF YOU HAVE ANSWERED YES TO SS1 AND YES TO SS2, THEN PROCEED WITH THE INFORMED CONSENTING PROCESS. PLACE THE UNIQUE ID STICKER ON THE INSTRUMENT AND ON THE HOUSEHOLD CONSENT FORM A.**			
	Confirm consent was granted<br><br>*Draw a check mark if consent was granted.*		
**IF CONSENT WAS GRANTED, PLACE A SECOND UNIQUE ID STICKER ON THE PAPER VERSION OF THE INSTRUMENT.**			

SURVEY ID

MODULE A. LOCATION

INSTRUCTIONS: Complete before administering the rest of the survey

NO.	FIELD	CODE	RESPONSE
A1	Province	EASTERN (1)<br>SOUTHERN (2)<br>LUAPULA (3)	
A2	District	CHOMA (1)<br>KALOMO (2)<br>NYIMBA (3)<br>PEMBA (4)<br>LUNDAZI (5)<br>MANSA (6)<br>CHEMBE (7)	
A3	Health Facility Catchment Area	CHOMA DISTRICT<br>CHOMA GENERAL (801001)<br>MANGUNZA (801019)<br>MACHA MISSION (801002)<br>MASUKU MISSION (801021)<br>MBABALA (801022)<br>MOCHIPAPA (801023)<br>SIMAKUTU (801043)<br>KALOMO DISTRICT<br>CHIFUSA HC (804023)<br>CHILALA HC (804024)<br>DIMBWE HC (804019)<br>HABULILE HC (804032)<br>KALOMO DISTRICT HOSPITAL (804002)<br>KANCHELE HC (804014)<br>MAWAYA HC (804034)<br>MOONDE HP (804042)<br>MUKWELA HC (804020)<br>SIACHITEMA HC (804013)<br>PEMBA DISTRICT<br>JEMBO (801413)<br>MUZOKA (801419)<br>NYIMBA DISTRICT<br>CHIPEMBE RHC (307010)<br>HOFMEYR ZONAL HC (307011)<br>KACHOLOLA RHC (307012)<br>MKOPEKA RHC (307016)<br>NYIMBA DISTRICT HOSPITAL (307001)<br>MANSA DISTRICT<br>FIMPULU (403017)<br>KABUNDA (403018)<br>LUBENDE (403041)<br>MANO (403026)<br>MANSA GENERAL HOSPITAL (403001)<br>MIBENGE (403029)<br>MUSAILA (403030)<br>MUTITI (403031)<br>MUWANGUNI (403032)<br>CHEMBE DISTRICT	

		KUNDAMFUMU (403023)<br>LUKOLA (403037)<br>LUNDAZI DISTRICT<br>CHIKOMENI (405026)<br>KAMSARO (305034)<br>KAPICHILA (305023)<br>LUKWISIZI (305040)<br>LUNDAZI HOSPITAL (305032)<br>LUSUNTHA (305021)<br>MWASE LUNDAZI ZONAL (305011)<br>NKHANGA (305046)<br>NYANGWE (305020)<br>PHIKAMALAZA (305031)<br>ZUMWANDA (305024)	
A4	Village Name<br><br>*Write in the name of the village.*		
GPS COORDINATES, TAKE 1			
A5	Latitude		
A6	Longitude		
GPS COORDINATES, TAKE 2			
A7	Latitude (decimal format)		
A8	Longitude (decimal format)		
A9	Date of Interview (DD/MM/YYYY)		
A10	Start time of interview<br>(24:00 format)		

SURVEY ID

MODULE B. HOUSEHOLD ENUMERATION

INSTRUCTIONS: Confirm that the person who you are speaking with is the head of the household or the head woman of the household.

INTERVIEWER: *"sono paliko makani yapachanya chanya nkupenja kuti nimanye pa imwe mwebenoecho banyumba na pa banthu abo imwe mukunkra nabo pankhomo linu. Mu mafumbo agha, rekani tichitore t kuti nyumba panji nkhomo mbanthu abo bakukhara pamoza mumaro yamoza, banthu abo bangaba mbahibare keniso bangaba bachibari cha, balikuzomera muthu uyo kukhara mutu wanyumba kwenisa bakwenera kuti abo bathu bakuphika naku ryera pamoza*

NO.	QUESTION	POTENTIAL RESPONSES	SKIP
B1	Kasi mukaba navilimika vilinga vyakubabika panyengo yakumasinda uku pazuba kufuma pa dazi lakukumbukira chaka chakubabika mukababikirapo?<br><br>*Unit of response in years.*		
B2	Kasi muli kusambililapo sukulu lili lose?	YES (1)<br>NO (0)<br>DON'T KNOW (96)	If (0) or (96), skip to B4
B3	Kasi mukafika mu giledi uli?<br><br>*If <1 year completed, write down 00. If >12 years completed, write down 13.*	DON'T KNOW (96)	
B4	Kasi mukusopa khu?	CATHOLIC (1)<br>PROTESTANT (2)<br>MUSLIM (3)<br>OTHER (SPECIFY) (4)	
B5	Kasi ndimwe ba mutundu uli?		
B6	Kasi ndimwe bakutora panji bakutengwe panji yayi, ndimwe chokoro panji mulikupauna munthengwa nyengo yichoko panji mulikurekana?	MARRIED/COHABITING (1)<br>DIVORCED (2)<br>SEPARATED (3)<br>WIDOWED (4)<br>NEVER-MARRIED (5)	If (2), (3), (4), or (5), skip to B8
B7	*If respondent is the male head of household:*<br>Kasi bose banakazi abo mulikutora kusazgirako na vibwezi wuwo mbaringa?<br><br>*If respondent is NOT male head of household:*<br>Kasi benecho banyumba bali balina banakazi balinga, kusazgirako na vibwezi wowo?		

SURVEY ID

B8	Kasi mbalinga Bana ba sungwana na banyamata abo bali navilimika vinkhonde (5) kwiza pasi pakhomo pinu?<br><br>*Include children who are in boarding school at the moment. If none, write down 00.*	BOYS<br>GIRLS	
B9	Kasi mbalinga Bana ba sungwana na banyamata abo bali navilimika kuyabira pa vinkhonde (5) kufika pavilimika khumi na vinayi (14) pakhomo pinu?<br><br>*Include children who are in boarding school at the moment. If none, write down 00.*	BOYS<br>GIRLS	
B10	Kasi mbalinga Banarumi nabanakazi abo bali navilimika kuyabira pakhumi limoza navinkhondi (15) kufika pa vilimika makhumi ghanayi navinkhondi navinayi pachanya (49) pakhomo pinu?	MEN<br>WOMEN	
	Kasi mbalinga Banarumi nabanakazi abo bali navilimika kuyabira pakhumi limoza navinkhondi (15) kufika pa vilimika makhumi ghanayi navinkhondi navinayi pachanya (49) pakhomo pinu? Kusazgirako na abo balikutayika mukati kati mu miyezi khumi limoza nayibli (12) yajumpapo kumasinda uku (cilimka chimoza)	MEN<br>WOMEN	
B11	Kasi mbaringa Banarumi nabanakazi abo bali navilimika kuyabira makhumi yankhondi (50) kufika pa vilimika makhumi yakhondi nakhumi lizoza na vinayi (64) pankhomo pinu	MEN<br>WOMEN	
B12	Kasi mbaringa Banarumi nabanakazi abo bali navilimika makhumi yakhondi nakhumi lizoza na vinkhondi pachanya (65) pakhomo pinu	MEN<br>WOMEN	
**INSTRUCTIONS:** Count and record the total number (B8 to B12) of household members.			

B13	Rekani sono abo bazgoranga mafumbo bapanikizgire apo imwe mukufumba fumbo ili: Kasi pankhomo pinu bathu bose pamoza bali mphendwa mwakuti (zunurani mphendwa)?	YES (1)<br>NO (0)	

SURVEY ID

INSTRUCTIONS: Ask the respondent to list the names of all women aged 15-49 in the household, including those who passed away in the last 12 months (1 year). Emphasize that you are also looking for information on individuals who have passed away in the last 12 months (1 year). Fill out column A with all names provided, and then continue to answer B-F for each person before selecting a respondent.

INTERVIEWER: *"panyengo yino yasono nkukhumba kuti munizunurire* mazina yabanakazi bose abo bali navilimika vyakubabika kuyabira pakhumi limoza navinkhondi (15) kufika pa vilimika makhumi makhumi yanayi navinkhondi navinayi pachanya (49) abo kanandi bakunkhara panyumba pano. Nabeya musazgireko namazina yabanakazi abo *balikutayika* mukati kati mu miyezi khumi limoza nayibili (12) yajumphapo kumasinda uku (cilika chimoza)."

TABLE 1. ROSTER OF WOMEN AGED 15-49 YEARS

	A. Munizunurire mazina yakwamba yabanakazi bose abo bali navilimika vyakubabika kuyabira pakhumi limoza navinkhondi (15) kufika pa vilimika makhumi y ghanayi navinkhondi navinayi pachanya (49) abo kanandi bakunkhara pankhomo pano. Kusazgirako na abo balikutayika mukati kati mu miyezi khumi limoza nayibli (12) yajumpapo kumasinda uku (cilimka chimoza<br><br>***Ensure the number includes those who would have been living there if they didn't pass away/move away in the past 12 months.*** *The number of women in this list should be greater or equal to the number of women in **B10**.*	BKasi mukat mukati kati ka miyezi khumi limoza nayibli (12) yajumpapo kumasinda uku (cilimka chimoza), uyu ngana (mumuzunure zina) wakbapo nathumbo iyo yikafikako masabata makhumi yatatu nayankhonzi (masamba 35)? Ndko kung'anamura kuti uyu ngana (mumuzunure zina) wakaba napakati panyongo yakuti kukankhara waka masabata yitatu (3) pafupi fupi kuti wababe?<br><br>*That is, was (name) pregnant at least up until ~3 weeks before her estimated delivery date?*<br><br>*Instructions: Note that this includes a delivery, still birth, neonatal death, etc.at any point within the past 12 months*<br>YES (1)<br>NO (0)<br>DON'T KNOW (96)<br><br>*If (0) or (96), skip to next person.*	C. Kasi ngana (mumuzunure zina, wachali ngwamoyo ?<br><br>YES (1)<br>NO/ DON'T KNOW (0)<br><br>*If (1), skip to E.*<br><br>**Check list and input name*	D. Kasi mungankhumba or somebody else kuzgorakomafumba ya choko paza thumbo ya ngana (mumuzunure zina) mwakumwimirirako?<br><br>YES (1)<br>NO (0)<br><br>*If (0), skip to next person.*	E. Is (*name) potentially eligible to take the survey?<br><br>*If **B=1** and (**IF APPLICABLE) D=1**, mark the box below.*<br><br>**Check list and input name*	**AFTER ALL WOMEN HAVE BEEN LISTED, TO SELECT A RESPONDENT:**<br><br>1. Roll the die<br>2. From the 1st checked box in Column E, count up to the rolled number, beginning again at the 1st checked box if needed until number is reached<br>3. Roll the die again<br>4. From the checked box you landed on after the 1st roll, count up to the 2nd rolled number, beginning again at the 1st checked box if needed until the 2nd number is reached<br>5. Select this woman<br>6. If woman selected is ALIVE, proceed to **Question B24**<br>7. If woman selected is DECEASED, proceed to Proxy Household Survey
B14					☐	
B15					☐	
B16					☐	
B17					☐	
B18					☐	
B19					☐	
B20					☐	

SURVEY ID

B21					☐	
B22					☐	
B23					☐	

NO.	QUESTION	POTENTIAL RESPONSES	SKIP
B23A	How old is (name)?		
B24	Kasi ngana (mumuzunure zina) ngwakwenera kutoramo chigaba mu chisambizgo ichi?	YES (1)<br>NO (0)<br>DON'T KNOW (96)	If (1), skip to consent then proceed to B27
B25	Kasi tingapangana kuti tiza penjaso siku panji nyengo yinyakhe tizakizeso penepapo iye wangasangikapo?	YES (1)<br>NO (0)<br>DON'T KNOW (96)	If (0) or (96), resample from potentially eligible women in TABLE 1
B26	Were you able to reschedule another time?	YES (1)<br>NO (0)	If (0), resample from potentially eligible women in TABLE 1
If you are unable to reschedule a time to come back and survey the sampled woman, go back to TABLE 1 and resample another potentially eligible woman. If you are re-visiting the household a subsequent time and the woman is now available, **proceed from Question B27**.			

INSTRUCTIONS: Make sure to obtain consent or assent (if the sampled woman is 15, 16 or 17 years old – refer to B23A), including a signature, from the sampled woman. If the woman is not able to sign, please have the woman provide a thumbprint. These questions will determine whether or not the sampled woman is eligible to proceed to the full household survey. If she is ineligible, then re-sample from Roster Table 1. If there are no more potentially eligible women to sample from, thank the woman and move on to the next household. If she is eligible, proceed to Module C.

STOP: MAKE SURE CONSENT OR ASSENT WAS OBTAINED FROM (NAME). PLACE A THIRD UNIQUE ID STICKER ON THE CONSENT FORM B – FOR THE ELIGBLE WOMAN.

INTERVIEWER: "Nawonga kuti mwazomera kuchitako chisambiriro chinthu ici. Sono niti nimu fumbaninge mafumbo gha choko pankhani ya kuphokwa uko kukamuchitirkirani sono sona apa."

NO.	QUESTION	POTENTIAL RESPONSES	SKIP
B27	Kasi mwana winu ngwamoyo?	YES (1)<br>NO (0)<br>DON'T KNOW (96)	If (1), continue to Module C<br>If (96), skip to B29
B28	Kasi mwana wakatayika pa uri?	BEFORE OR ON DAY OF DELIVERY (1)<br>WITHIN ONE MONTH AFTER DELIVERY (2)<br>OTHER (3)	If (2) or (3), continue to Module C
B29	Kasi mwana winu wakababika mabere nyengo iyo mukarindizgango kuti wababike yindafike?	YES (1)<br>NO (0)<br>DON'T KNOW (96)	If (0), continue to Module C<br>If (96), end and re-sample from Roster Table 1
B30	Kasi padazi ilo mwana wakababikira, Kukakhara masabata yalinga pa mbere nyengo iyo mukarindizganga kuti muphokwe yindafike?	<= 3 WEEKS (1)<br>>3 WEEKS (2)<br>DON'T KNOW (96)	If (1), continue to Module C<br>If (2), end and re-sample from Roster Table 1<br>If 96, end and re-sample from Roster Table 1

SURVEY ID

MODULE C. DEMOGRAPHICS

INSTRUCTIONS: After eligible respondent has been randomly sampled from all eligible respondents, proceed with the instrument. Ensure that the woman selected to proceed with the survey has delivered a child **within the last year**. This section is to get basic demographics on the household and the respondent.

INTERVIEWER: "sono niti ni mufumbaninge mafumbo pa imwe mwabenecho na pa aba bakukhala pakhomo pinu wuwo."

NO.	QUESTION	POTENTIAL RESPONSES	SKIP
C1	Kasi imwe ndimwe benecho panji mutu wa nyumba?	YES (1)<br>NO (0)	If (1), skip to C9
C2	Kasi benecho panji mutu wanyumba muli nabo uli?	SPOUSE (1)<br>CHILD (2)<br>GRANDCHILD (3)<br>NIECE (4)<br>AUNTIE/OTHER RELATIVE (5)<br>OTHER (SPECIFY) (6):	
C3	Kasi muli kurutapo kusukulu?	YES (1)<br>NO (0)<br>DON'T KNOW (96)	If (0) or (96), skip to C5
C4	Kasi ni giledi uli yapachanya chomene yeneiyo imwe mukamarizga?<br><br>*If <1 year completed, write d own 00. If >12 years completed, write down 13.*	DON'T KNOW (96)	
C5	Kasi mukusopa nkhu?	CATHOLIC (1)<br>PROTESTANT (2)<br>MUSLIM (3)<br>OTHER (SPECIFY) (4):	
C6	Ndimwe ba mutundu uli?		
C7	Kasi mulikutengwa panji mukatenwapo nthengwa yiri kumara, mulikupatukana panyengo yichoko, ndimwe chokoro, panji mundatengwepo?<br><br>*If don't know, write down 96.*	MARRIED/COHABITING (1)<br>DIVORCED (2)<br>SEPARATED (3)<br>WIDOWED (4)<br>NEVER-MARRIED (5)	If (2), (3), (4) or (5), skip to C9
C8	Kasi barumi binu bali bananakazi balinga kusazgiraka navibwezi wuwo?		
C9	Kasi mulikusangikapo kalinga napa thupi kalinga?		
C10	Kasi bana bamoyo abo muli kubabapo mbalinga?		

INTERVIEWER: "sonno rekani tidumbirane za panyumba yinu."

SURVEY ID

NO.	QUESTION	POTENTIAL RESPONSES	SKIP
C11	Kasi maji agho mukumwa pano pankhomo pano chomeneme yakufuma khu?	*PIPED WATER*<br>PIPED INTO DWELLING (1)<br>PIPED TO YARD/PLOT (2)<br>PUBLIC TAP/STANDPIPE (3)<br>TUBE WELL OR BOREHOLE (4)<br>*DUG WELL*<br>PROTECTED WELL (5)<br>UNPROTETED WELL (6)<br>*WATER FROM SPRING*<br>PROTECTED SPRING (7)<br>UNPROTECTED SPRING (8)<br>RAINWATER (9)<br>TANKER TRUCK (10)<br>CART WITH SMALL TANK (11)<br>SURFACE WATER (12)<br>(RIVER/DAM/LAKE/POND/STREAM/CANAL/<br>IRRIGATION CHANNEL)<br>BOTTLED WATER (13)<br>OTHER (PLEASE SPECIFY) (14):	If (13), skip to C14
C12	Kasi malo agho mukuneya maji yali dera nji?	IN OWN DWELLING (1)<br>IN OWN YARD/PLOT (2)<br>ELSEWHERE (3)	If (1) or (2), skip to C14
C13	Mukutora nyengo yitari uli kuya kaneya maji uko mkutora panji mukuneya, nakuraso pankhomo?	MINUTES (1): ☐☐☐☐<br>DON'T KNOW (96)	
C14	Maji agho, kasi mukuchitapo nayo vilivose kuti yakhare yambura vikho mumwe?	YES (1)<br>NO (0)<br>DON'T KNOW (96)	
C15	Kanandi kanandi, nchimbuzi chamutundu uli icho imwe pano pankhomo mukutebeteska?	*FLUSH OR POUR FLUSH TOILET*<br>FLUSH TO PIPED SEWER SYSTEM (1)<br>FLUSH TO SEPTIC TANK (2)<br>FLUSH TO PIT LATRINE (3)<br>FLUSH TO SOMEWHERE ELSE (4)<br>FLUSH, DON'T KNOW WHERE (5)<br>*PIT LATRINE*<br>VENTILATED IMPROVED PIT LATRINE (6)<br>PIT LATRINE WITH SLAB (7)<br>PIT LATRINE WITHOUT SLAB/OPEN PIT (8)<br>COMPOSTING TOILET (9)<br>BUCKET TOILET (10)<br>HANGING TOILET/HANGING LATRINE (11)<br>NO FACILITY/BUSH/FIELD (12)<br>OTHER (SPECIFY) (13):	
C16	Chimbuzi ichi kasi mukutebeteska pamoza na banthu ba mankhomo yanyakhe?	YES (1)<br>NO (0)	

C17	Kasi nyumba yinu yiliko navinthu vili vyose pavinthu ivi<br><br>*Item must be functioning usually:*		YES (1)	NO (0)	DON'T KNOW (96)
	A	MAGESI	☐	☐	☐
	B	MAGESI YOSEBESEZGELA ZUBA	☐	☐	☐

C	MUCHINI WOBUSKIRA MAGESI	☐	☐	☐
D	NYARI YOBUSKIRA NA MAFUTA	☐	☐	☐
E	MUNCHUKO PANJI CHIBIYA CHOSEBEZESKERA NAMA GESI	☐	☐	☐
F	CHONTHUKIZGIRAMO VAKURYA CHA MAGESI	☐	☐	☐
G	CHITOVU CHA CHAMALASHA	☐	☐	☐
H	CHITOVU CHAVU CHA NKHUNI	☐	☐	☐
I	ICITOFU CAMALAITI	☐	☐	☐
J	MOBILE TELEPHONE	☐	☐	☐
K	MATILESI	☐	☐	☐
L	MUPANDO	☐	☐	☐
M	THEBULO	☐	☐	☐
N	KABATI	☐	☐	☐
O	MUPANDO WOTUFUKA TOFUKA	☐	☐	☐
P	NKHOLOKO	☐	☐	☐
Q	FANI	☐	☐	☐
R	MASHINI YOTIMILAKO VYOVWALA	☐	☐	☐
S	NETI YOVIKILILAKO NYIMBU	☐	☐	☐
T	INTERNET	☐	☐	☐
U	NKHOLOKO YAPAWOKO	☐	☐	☐
V	AKAWUNTI YOBIKAMO NDALAMA KU BANKI	☐	☐	☐
W	GEJO	☐	☐	☐
X	WINIBALA	☐	☐	☐
Y	KAUCHINI KOPERERAKO	☐	☐	☐
Z	TILAKITA YOLIMILAKO	☐	☐	☐
AA	CHIGAYO	☐	☐	☐
BB	FOSHOLO	☐	☐	☐
CC	BEMBA	☐	☐	☐
DD	PIKI	☐	☐	☐
EE	PAMPU YA MAJI	☐	☐	☐

FF	THENGERE LOLIMAKO	☐	☐	☐
GG	KAKHUNI YAVIPAMBI	☐	☐	☐
HH	CHILIMBA	☐	☐	☐
II	CHILIMBA CHAVITHUZITHUZI	☐	☐	☐
JJ	PHONI YAKUMAWOKO	☐	☐	☐
KK	PHONI YAMUNYUMB	☐	☐	☐
LL	MUCHIN WAMUTUNDU WA KOMPWUTA	☐	☐	☐
MM	CHILIMBA CHAMAMA TEPU	☐	☐	☐
NN	CHILIMBA CHOFUMYA MAZGO NA VITHUZITHUZI PARA MWAVIBANYA NAVILIMBA VINYAKHE	☐	☐	☐
OO	NJIINGA	☐	☐	☐
PP	NJINGA YAMOTO	☐	☐	☐
QQ	NGORO	☐	☐	☐
RR	GALIMOTO	☐	☐	☐
SS	BOTI YA MUCHINI	☐	☐	☐
TT	BOTI YAMUTHIKO	☐	☐	☐

C18	Kanandi kanandi, nimoto wamutundu uri pankhomo pinu uwo mukusebezeska pophika?	ELECTRICITY (1)<br>SOLAR POWER (2)<br>LIQUID PROPANE GAS (LPG) (3)<br>NATURAL GAS (4)<br>BIOGAS (5)<br>KEROSENE (6)<br>COAL, LIGNITE (7)<br>CHARCOAL (8)<br>WOOD (9)<br>STRAW/SHRUBS/GRASS (10)<br>AGRICULTURAL CROP (11)<br>ANIMAL DUNG (12)<br>NO FOOD COOKED IN HOUSEHOLD (13)<br>OTHER (SPECIFY) (14):	If (13), skip to C20
C19	Kasi panyumba pano, muphikirankhu?	IN THE HOUSE (1)<br>IN A SEPARATE BUILDING (2)<br>OUTDOORS (3)<br>OTHER (SPECIFY) (4):	
C20	Pozenga nyumba yinu pasi, kasi bakasebezeska vivichi?<br><br>*OBSERVE THE FLOOR TO CONFIRM. (If more than one material, select the one that is "most" common)*	NATURAL FLOOR<br>EARTH/SAND (1)<br>DUNG (2)<br>RUDIMENTARY FLOOR<br>WOOD PLANKS (3)<br>PALM/BAMBOO/REEDS (4)<br>FINISHED FLOOR<br>PARQUET/POLISHED WOOD (5)	

		VINYL (PVC) OR ASPHALT STRIPS (6)<br>CERAMIC/TERRAZZO TILES (7)<br>CONCRETE CEMENT (8)<br>CARPET (9)<br>OTHER (SPECIFY) (10):	
C21	Nga mutenje wanyumba yinu yose bakapangila vivichi?<br><br>*OBSERVE THE ROOF TO CONFIRM. (If more than one material, select the one that is "most" common)*	*NATURAL ROOFING*<br>NO ROOF (0)<br>THATCH/PALM LEAF (1)<br>*RUDIMENTARY ROOFING*<br>RUSTIC MAT (2)<br>PALM/BAMBOO (3)<br>WOOD PLANKS (4)<br>CARDBOARD (5)<br>*FINISHED ROOFING*<br>METAL/IRON SHEETS (6)<br>WOOD (7)<br>CALAMINE/CEMENT FIBRE (ASBESTOS) (8)<br>CERAMIC/HARVEY TILES (9)<br>CEMENT (10)<br>ROOFING SHINGLES (11)<br>MUD TILES (12)<br>OTHER (SPECIFY) (13):	
C22	Kasi vithu uli ivyo ivyo bakazengela nyumba kuwalo?<br><br>*OBSERVE THE WALLS TO CONFIRM. (If more than one material, select the one that is "most" common)*	*NATURAL WALLS*<br>NO WALLS (0)<br>CANE/PALM/TRUNKS (1)<br>MUD (2)<br>*RUDIMENTARY WALLS*<br>BAMBOO/POLE WITH MUD (3)<br>STONE WITH MUD (4)<br>PLYWOOD (5)<br>CARDBOARD (6)<br>REUSED WOOD (7)<br>*FINISHED WALLS*<br>CEMENT (8)<br>STONE WITH LIME/CEMENT (9)<br>BRICK (10)<br>CEMENT BLOCKS (11)<br>WOOD PLANKS (12)<br>OTHER (SPECIFY) (13):	
C23	Kasi pali waliyose pamunyumba mwinu uyo wali nathengere lake panji munda wolorimapo	YES (1)<br>NO (0)<br>DON'T KNOW (96)	If (0) or (96), skip to C25
C24	Kasi Nthengere laulimi ndikulu kufika malima, mahekitara, ma mitazi yangati ilo bamunyumba yinu balinalo?	[ ][ ] [ ][ ][ ][ ]<br>LIMA (1) QUANTITY<br>ACRES (2)<br>HECTARES (3)<br>SQUARE METERS (4)<br>DON'T KNOW (96)	

C25	Nimphenwa uliyavibeto ivo mulinavo panyumba ynu?	NUMBER	NONE (00)	DON'T KNOW (96)

SURVEY ID

	A	NGOMBE ZACIMUZI?		☐	☐
	B	BONGWANI		☐	☐
	C	NGOMBE ZOLYAKO DENDE		☐	☐
	D	NGAMERA		☐	☐
	E	MBUZI		☐	☐
	F	MBERERE		☐	☐
	G	NKHUMBA		☐	☐
	H	NKHUKU/VIBETO VINYAKHE VAVIYUNI		☐	☐
	I	BAKALULU		☐	☐
	J	VIBETO VINYAKE		☐	☐

NO.	QUESTION	POTENTIAL RESPONSES	SKIP
C26	Rongorani nthowa izo Mukusangiramo ndarama imwe nabamunyumba yinu.<br>.<br><br>*Select all that apply.*<br>.	SALARIED EMPLOYMENT (1)<br>SMALL BUSINESS, SHOP OR KIOSK (2)<br>SMALL HOUSEHOLD INCOME GENERATING ACTIVITY (3)<br>DOWRY (4)<br>SALE OF CROPS/ANIMALS (5)<br>SALE OF ASSETS (6)<br>REMITTANCES (CASH DONATIONS FROM FRIENDS/FAMILY) (7)<br>GOVERNMENT/NGO AID, GRANT OR OTHER FINANCIAL SUPPORT (8)<br>CASUAL DAILY WORK (9)<br>OTHER (SPECIFY) (10):	
C27	Usange ba pankhomo pano bankhara na khumbiro lokweleta ndalama kubanki panji tubungwe uto tukwereteska ndalama (kufumyira ko babwezi naba bari binu), Kasi bapankhomo pano, bangaba nazo nkhongo zokweleka ndalama?	NO (0)<br>PROBABLY NOT (1)<br>PROBABLY YES (2)<br>DEFINITELY YES (3)<br>DON'T KNOW (96)	
C28	Kasi banakazi bose abo bali pakati pamoza nabana abo bali navilimika vinkhondi (5) kuruta kuchanya pogona usiku wajumpha uwu wamailo bakachvivilirako muneti yanyimbu?	YES (1)<br>NO (0)<br>DON'T KNOW (96)	
C29	Kasi pakhomo pano, mukukwaniriska	YES (1)	

	kulipira ndalama zokhumbika kumasukulu yabana navose ivo vikukupenjeka pamasukulu ghabo?	USUALLY (2)<br>SOMETIMES (3)<br>RARELY (4)<br>OTHER (SPECIFY) (5):<br>NO (0)	
C30	Mumwezi umoza uwo wajumphapo kumanyuma uku, Kasi paliko yumoza uyo wakagonerapo nanjarapo, muhanya wose nausiku wuwo kwambura kuryapo kanthu?	YES (1)<br>NO (0)<br>DON'T KNOW (96)	
C31	Kasi paliko mwana waliyose munyumba yinu uyo wakagona nayo najara mailo kwambura kurya?	YES (1)<br>NO (0)<br>DON'T KNOW (96)	
C32	Kasi nyumba yinu yingankora kuchimphepo panji civura chikuru kwambura nyumba kunangika?	YES (1)<br>NO (0)<br>DON'T KNOW (96)	
C33	Para vuru yulokwa, kasi maji Para yothonya yothonyerako dela lanyumba uko bana bogonera?	YES (1)<br>NO (0)<br>DON'T KNOW (96)	

MODULE D. LAST DELIVERY/MOTHERS' SHELTER

INTERVIEWER: "Sono nimufumbaninge mafumbo yokhwaskana pa khani yophokwa panyego yifupifupi iyo mupaphokwerapo kumasinda uko. chakwamba, mutore kanyengo muganizirepo panyengo yira pambere mundaphokwe kweniso na pakuphokwa kwenecho. Pamanyuma, tidumbiraneke pa za cipanda odi cha bamama abo balipakati kulindizgira kuphokwa. Mwanozgeka kuti tiyambe?"

NO.	QUESTION	POTENTIAL RESPONSES	SKIP
D1	Munyengo yajuphapo, kasi nidazi uli ilo mukaphokwerapo? (DD MM YYYY)<br><br>*If date not know, ask for under 5 card. If no under 5 card, write 15th*	____ ____ ____ ____ ____ ____ ____ ____<br>D D M M Y Y Y Y	
D2	Kufumirako nyengo yino yasono, kasi kumanyuma uku muliporikapo za chipanda odi cha bamama abo bali pakati balindizga kuwombokwa?	YES (1)<br>NO (0)<br>DON'T KNOW (96)	If (0) or (96), skip to D18
D3	Nikhu/khawnjani uko imwe mukapurikapo za chipanda odi cha bamama abo balipakati bakulindizga kuphokwa?<br><br>*(Select all that apply)*	CHIEF (1)<br>HEADMEN (2)<br>HEALTH CARE WORKER (3)<br>SMAG (4)<br>TRADITIONAL BIRTH ATTENDANT (5)<br>FAMILY MEMBER (6)<br>ANOTHER MOTHER (7)<br>OTHER COMMUNITY MEMBER (8)<br>RADIO (9)	

SURVEY ID			

		OTHER (SPECIFY) (10):	
D4	Ghanaghanani pa nyengo ya uvyazi uwo tikudumbapo, kasi mukankharapo mu chipanda odi chabamama abo balipakiti pachifukwa chiri chose pambere munda phokwe panji pamasinda yophokwa?	YES (1)<br>NO (0)	If (1), skip to D5
D4a	If NO, why?<br><br>*Select all that apply.*	NO MOTHERS SHELTER (1)<br>NO PERMISSION FROM HUSBAND OR FAMILY (2)<br>NO MONEY (3)<br>POOR QUALITY (4)<br>NOT CLEAN (5)<br>TOO CROWDED (6)<br>NOT CULTURALLY APPROPRIATE (7)<br>NOT SAFE (8)<br>DELAYS DELIVERY (10)<br>DIDN'T KNOW ABOUT MOTHERS SHELTER (11)<br>OTHER (12)<br>IF OTHER, SPECIFY	Skip to D18

INSTRUCTIONS: Ask the respondent for what reason(s) did she stay at a mothers' shelter, and then prompt her with the reasons listed below.

D5		Kasi mukuganizira kuti mukankharamo masiku ghalinga muchipanda odi chabama abo bali pakati, pachifukwa chiri chose ico mungawonga pa ivi vili apa?	NUMBER OF NIGHTS	NONE (0)	DON'T KNOW (96)
	A	KUCHIPIMO CHAKWAMBA	☐☐	☐	☐
	B	VIPIMO VINYAKHE	☐☐	☐	☐
	C	PAKULINDIZGA NYENGO YOPHOKWERAPO	☐☐	☐	☐
	D	PAMASINDA YANYENGO IYO NKHAFUMIRA MUCHIPATARA/ PANJI APO NKHATI PHOKWE WAKA	☐☐	☐	☐
	E	PAMAZUBA YATATU (3) APO NKHARUTA KUCHIPIMO PAMASINDA YANYENGO NKHATI NA PHOKWA	☐☐	☐	☐
	F	KUYAMBIRA MUSABATA YIMOZA KUFIKA MUMASABATA YABIL (7-14) APO NKHARUTA KUCHIPIMO PAMASINDA YOPHOKWA	☐☐	☐	☐

G	MASAMBATA YAKHONDE NA YIMOZA PACHANYA (6) YACHIPIMO ICHO NKHARUTAKO PAMASINDA YOPHOKWA KALE	[ ][ ]	☐	☐
H	YINYAKHE (ZUNURNI)	[ ][ ]	☐	☐

NO.	QUESTION	POTENTIAL RESPONSES	SKIP
D6	Kasi nchipanda odi chankhuti icho imwe mukankharapo nyengo yitari chomene apo mkati mwaruta kunkhara kuchibanda odi muku wakwamba?<br><br>*Confirm the longest number of nights the respondent stayed at a mothers' shelter.*	CHOMA DISTRICT<br>CHOMA GENERAL (1)<br>MANGUNZA (2)<br>MACHA MISSION (3)<br>MASUKU MISSION (4)<br>MBABALA (5)<br>MOCHIPAPA (6)<br>SIMAKUTU (7)<br>KALOMO DISTRICT<br>CHIFUSA HC (8)<br>CHILALA HC (9)<br>DIMBWE HC (10)<br>HABULILE HC (11)<br>KALOMO DISTRICT HOSPITAL (12)<br>KANCHELE HC (13)<br>MAWAYA HC (14)<br>MOONDE HP (15)<br>MUKWELA HC (16)<br>SIACHITEMA HC (17)<br>PEMBA DISTRICT<br>JEMBO (18)<br>MUZOKA (19)<br>NYIMBA DISTRICT<br>CHIPEMBE RHC (20)<br>HOFMEYR ZONAL HC (21)<br>KACHOLOLA RHC (22)<br>MKOPEKA RHC (23)<br>NYIMBA DISTRICT HOSPITAL (24)<br>MANSA DISTRICT<br>FIMPULU (25)<br>KABUNDA (26)<br>LUBENDE (27)<br>MANO (28)<br>MANSA GENERAL HOSPITAL (29)<br>MIBENGE (30)<br>MUSAILA (31)<br>MUTITI (32)<br>MUWANGUNI (33)<br>CHEMBE DISTRICT<br>KUNDAMFUMU (34)<br>LUKOLA (35)<br>LUNDAZI DISTRICT<br>CHIKOMENI (36)<br>KAMSARO (37)<br>KAPICHILA (38)<br>LUKWISIZI (39)<br>LUNDAZI HOSPITAL (40)	

		LUSUNTHA (41)<br>MWASE LUNDAZI ZONAL (42)<br>NKHANGA (43)<br>NYANGWE (44)<br>PHIKAMALAZA (45)<br>ZUMWANDA (46)<br>OTHER (SPECIFY NAME OF HEALTH FACILITY AND DISTRICT) (47):	

***INTERVIEWER**: "Sono nifumbaninge umo mukakhaliranga kuchiPanda odi chabamama bapakati. Torani kanyengo muganizire umo mukankhariranga kura. Kasi Mwanozgeka Kuti tiyambe?"*

D7	Muwunkhaliro winu panyengo yakuchipanda odi kasi……		YES (1)	NO (0)	DON'T KNOW (96)
	A	KUKAPIKAKO KWA IMWE BEDI PANJI MATILESI	☐	☐	☐
	B	MUKAGONANGA PA BEDI PANJI MATILESI NA BATHU BANJ PANYENGO YILI YOSE	☐	☐	☐
	C	KSI PANYENGO YIRI YOSE, MUKAGONAPO MUKATI MU NET YOVIKILILA NYIMBU USIKU	☐	☐	☐
	D	KASI PARA MUKATI MWAFIKA, PAKUMPOKERERANI BAKAMURONGOSOLERANIPO MALAMULO NA NDODOMEKO YACHIBANDA ODI CHABAMAMA BA PAKATI	☐	☐	☐
	E	KASI AGHO MUKANEYANGA GHAKABA GHAWEMI	☐	☐	☐
	F	MUKATEBETESKANGAKO CILICHOSE KUBUSKA KUTI MUWONERENGEKO USIKU PARA KWAFIPA	☐	☐	☐
	G	CHIGEZERO NAPO CHAPHIRA PAKABAPO	☐	☐	☐
	H	MARO GHAWEMI APO MUKABIKANGA KATUNDU YINU PAMOZA NAVAKULYA YAKABAKO	☐	☐	☐
	I	MUSAMBIRAKO VISAMBIZGO PA UDOKO	☐	☐	☐

NO.	QUESTION	POTENTIAL RESPONSES	SKIP
D8	Chipembo chikabapo pachipanda odi chabamama bapakai?	Yes (1)<br>No (2)<br>Don't know (96)	If (0) or (96), skip to D10
D9	Agha malo ya chipembo yakabanga yakuvikiririka	Yes (1)<br>No (2)<br>Don't know (96)	
D10	Kasi mukasambirirako ruso	Yes (1)	If (0) or (96), skip to

	SURVEY ID		

	lulilose apo mukakharanga Kuchipanda odi chabamama abo bali pakai?	No (2)<br>Don't know (96)	D12
D11	Ni ruso uli mwasambililako?		
D12	Kasi baka muphalilanipo kuti mulipile ndalama kuti imwe munkhare kuchipanda old?	Yes (1)<br>No (2)<br>Don't know (96)	If (0) or (96), skip to D14
D13	Nizilinga ndalama (mumakwacha) mukalipila?		
D14	Kasi bakamuphalilani kubadalako kanthu kalikose kufumyako ndalama kuti imwe munkhare kuchipanda odi?	Yes (1)<br>No (2)<br>Don't know (96)	If (0) or (96), skip to D16
D15	Ni vithu uli ivo imwe mukabadala?<br><br>*(Select all that apply)*	LABOR (1)<br>LIVESTOCK/POULTRY (2)<br>FOOD OR OTHER AGRICULTURAL RESOURCES (3)<br>OTHER IN-KIND RESOURCES (SPECIFY) (4)<br>OTHER (SPECIFY) (5):	

D16	***INTERVIEWER:*** *"Sono nifumbaninge pamasuzgo gha anyengo nayengo agho bawomama bakusana nayo kuchipanda odi bankara nayo pambere bakulindizge nyengo yakuwombokwa. Apo nkhuzunura chimoza chimoza, munipharire para chimoza pa ivi nkhuzunura chikaba ni suzgo kwaime para mukakharanga kuchipanda odi chabamama abo bali pakati, ndipo para chiri ntheura, imwe muyerezgere ukulu wa suzgo ilo pakuni pharira kuti likaba lirara suzgo panji likaba lichoko suzgo kwa imwe."*				
		MAJOR PROBLEM (2)	MINOR PROBLEM (1)	NO PROBLEM (0)	UNDECIDED (96)
	A UWEMI UMO BAKACHIPAKIRA	☐	☐	☐	☐
	B KUPHERERERKA NAKA SUNGIRO	☐	☐	☐	☐
	C ODONGO	☐	☐	☐	☐
	D UBAPO WABA TENETI	☐	☐	☐	☐
	E CHIKUMBUMUTIMA CHABATEBETI	☐	☐	☐	☐
	F KUZOMEREZGA KUTEBETESKA CHIPEMBO	☐	☐	☐	☐
	G CHIMPHWIRIKITI CHABANTHU	☐	☐	☐	☐
	H NCHIRIKIZGO YA BAKUKHARAPO	☐	☐	☐	☐
	I MPHUKWA	☐	☐	☐	☐
	J KULINGANA NA MWAMBO WACHIKHARORO CHITHU	☐	☐	☐	☐

NO.	QUESTION	POTENTIAL RESPONSES	SKIP
D17	Parara munga	VERY SATISFIED (1)<br>MORE OR LESS SATISFIED (2)	

	yowoyapachisani sani, Kasi ndimwe bakukhutirana nayo uli makhariro ghapa chipanda odi cha bamama abo pali pakati bakulindizga nyengo yophokwa?	NOT SATISFIED (3)	
D17a	Do you intend to use mothers' shelter for future deliveries?	YES (1)<br>NO (0)<br>DON'T KNOW (96)	
D17b	Would you recommend using a mothers' shelter to friends or relatives?	YES (1)<br>NO (0)<br>DON'T KNOW (96)	

INTERVIEWER: *"Namuwongani pakuzgora mafumboyokwaskana na nkhani za chipanda odi chabamama abo bali pakati. sono tidumbirane pa za kuphokwa kwinu panyengo yajumpha sono sono kumasinda uku"*

D18	Mbanjani bakamovwirani pakuphokwa soso sono apa?<br><br>*(Select all that apply)*<br><br>*If respondent says NO ONE ASSSISTED, probe to determine whether any adults were present at the delivery. wakababilanga.*	DOCTOR/CLINICAL OFFICER (1)<br>NURSE/MIDWIFE (2)<br>OTHER HEALTH FACILITY STAFF/PERSONNEL (3)<br>TRADITIONAL BIRTH ATTENDANT (4)<br>SMAG (5)<br>RELATIVE/FRIEND/AUNTIE (6)<br>NO ONE ASSISTED (7)<br>OTHER (SPECIFY) (8):	
D18a	Where do you intend to deliver your next baby?	YOUR HOME (1)<br>OTHER HOME (2)<br>HEALTH POST/FACILITY (3)<br>HOSPITAL (4)<br>OTHER (SPECIFY) (5):	
D18b	Where did you intend to deliver for your last delivery?	YOUR HOME (1)<br>OTHER HOME (2)<br>HEALTH POST/FACILITY (3)<br>HOSPITAL (4)<br>OTHER (SPECIFY) (5):	
D19	Nikhu uko imwe mukaphokwera pa wauvyazi wasosososono kumanyuma uku?	YOUR HOME (1)<br>OTHER HOME (2)<br>HEALTH POST/FACILITY (3)<br>HOSPITAL (4)<br>OTHER (SPECIFY) (5):	If (1), (2) or (5), skip to D31
**INSTRUCTIONS:** If respondent answers **OTHER (5) to Question D19**, probe to ensure this is not a health post/facility or hospital.			

FACILITY-BASED DELIVERY			
**NO.**	**QUESTION**	**POTENTIAL RESPONSES**	**SKIP**
D20	Nkhuchipatala nchi uku muyambirapo kuruta pau vyzi winu wakuwumaliro kumanyuma uku?	CHOMA DISTRICT<br>CHOMA GENERAL (801001)<br>MANGUNZA (801019)<br>MACHA MISSION (801002)<br>MASUKU MISSION (801021)	

SURVEY ID

	(*INSTRUCTIONS: if woman reports hospital, probe to ensure she did not first present at a health facility and was transferred to hospital.)*	MBABALA (801022)<br>MOCHIPAPA (801023)<br>SIMAKUTU (801043)<br>KALOMO DISTRICT<br>CHIFUSA HC (804023)<br>CHILALA HC (804024)<br>DIMBWE HC (804019)<br>HABULILE HC (804032)<br>KALOMO DISTRICT HOSPITAL (804002)<br>KANCHELE HC (804014)<br>MAWAYA HC (804034)<br>MOONDE HP (804042)<br>MUKWELA HC (804020)<br>SIACHITEMA HC (804013)<br>PEMBA DISTRICT<br>JEMBO (801413)<br>MUZOKA (801419)<br>NYIMBA DISTRICT<br>CHIPEMBE RHC (307010)<br>HOFMEYR ZONAL HC (307011)<br>KACHOLOLA RHC (307012)<br>MKOPEKA RHC (307016)<br>NYIMBA DISTRICT HOSPITAL (307001)<br>MANSA DISTRICT<br>FIMPULU (403017)<br>KABUNDA (403018)<br>LUBENDE (403041)<br>MANO (403026)<br>MANSA GENERAL HOSPITAL (403001)<br>MIBENGE (403029)<br>MUSAILA (403030)<br>MUTITI (403031)<br>MUWANGUNI (403032)<br>CHEMBE DISTRICT<br>KUNDAMFUMU (403023)<br>LUKOLA (403037)<br>LUNDAZI DISTRICT<br>CHIKOMENI (405026)<br>KAMSARO (305034)<br>KAPICHILA (305023)<br>LUKWISIZI (305040)<br>LUNDAZI HOSPITAL (305032)<br>LUSUNTHA (305021)<br>MWASE LUNDAZI ZONAL (305011)<br>NKHANGA (305046)<br>NYANGWE (305020)<br>PHIKAMALAZA (305031)<br>ZUMWANDA (305024)<br>OTHER (SPECIFY NAME OF HEALTH FACILITY AND DISTRICT) (47):	

SURVEY ID

D21	Ninthowa uli yakendero mukatebeteska paruta Kumalo (muzunure zina la chipatala chidoko) uko mukababila panji kuwombokwera pamphapo iyo mababanayo sonoson	WALKING (1)<br>BICYCLE (2)<br>CARRIED IN WHEELBARROW (3)<br>ANIMAL-DRAWN CART (4)<br>TAXI (5)<br>CAR (6)<br>MOTORCYCLE (7)<br>AMBULANCE (8)<br>OTHER (SPECIFY) (9):	
D22	Apo mukatebeteska *[zunurani transipoti]*, Mukende nyengo yitali uli kuti mufike kuchipata la cidoko icho?<br><br>*Be sure to specify unit of response.*	HOURS MINUTES	
D23	Kasi mwana mukamubbila kuchipatala chidoko ico mukayambilirapo kurtako?	YES (1)<br>NO (0)<br>DON'T KNOW (96)	If (1) or (96) skip to D27
D24	Zunulani zina la chipatala chikulu panji chidoko ichoimwe mukababilako mwana?	CHOMA DISTRICT<br>CHOMA GENERAL (801001)<br>MANGUNZA (801019)<br>MACHA MISSION (801002)<br>MASUKU MISSION (801021)<br>MBABALA (801022)<br>MOCHIPAPA (801023)<br>SIMAKUTU (801043)<br>KALOMO DISTRICT<br>CHIFUSA HC (804023)<br>CHILALA HC (804024)<br>DIMBWE HC (804019)<br>HABULILE HC (804032)<br>KALOMO DISTRICT HOSPITAL (804002)<br>KANCHELE HC (804014)<br>MAWAYA HC (804034)<br>MOONDE HP (804042)<br>MUKWELA HC (804020)<br>SIACHITEMA HC (804013)<br>PEMBA DISTRICT<br>JEMBO (801413)<br>MUZOKA (801419)<br>NYIMBA DISTRICT<br>CHIPEMBE RHC (307010)<br>HOFMEYR ZONAL HC (307011)<br>KACHOLOLA RHC (307012)<br>MKOPEKA RHC (307016)<br>NYIMBA DISTRICT HOSPITAL (307001)<br>MANSA DISTRICT<br>FIMPULU (403017)<br>KABUNDA (403018)<br>LUBENDE (403041)<br>MANO (403026)<br>MANSA GENERAL HOSPITAL (403001)<br>MIBENGE (403029)<br>MUSAILA (403030)<br>MUTITI (403031)	

SURVEY ID

		MUWANGUNI (403032)<br>CHEMBE DISTRICT<br>KUNDAMFUMU (403023)<br>LUKOLA (403037)<br>LUNDAZI DISTRICT<br>CHIKOMENI (405026)<br>KAMSARO (305034)<br>KAPICHILA (305023)<br>LUKWISIZI (305040)<br>LUNDAZI HOSPITAL (305032)<br>LUSUNTHA (305021)<br>MWASE LUNDAZI ZONAL (305011)<br>NKHANGA (305046)<br>NYANGWE (305020)<br>PHIKAMALAZA (305031)<br>ZUMWANDA (305024)<br>OTHER (SPECIFY NAME OF HEALTH FACILITY AND DISTRICT) (47):	
D25	Kasi kuc chipatala ichi, bakamutumaniko waka mbatebeti abo bakuna pa va umoyo?	YES (1)<br>NO (0)<br>DON'T KNOW (96)	If (0) or (96), skip to D27
D26	Mukuyerezgera waka chikatora nyengo yitali uli ku bamutumani ku chipatala ichi?	LESS THAN 1 HOUR (1)<br>1 TO 2 HOURS (2)<br>MORE THAN 2 HOURS (3)	
D27	Mbanjani chomene chomene abo bakagamura kuti mukababile kuchipatala?	YOURSELF (1)<br>HUSBAND/PARTNER (2)<br>MOTHER/MOTHER-IN-LAW (3)<br>AUNTIE (4)<br>OTHER FAMILY MEMBER (5)<br>FRIEND (6)<br>OTHER (SPECIFY) (7):	
D28	Kasi Mukankhariskako kuchipatala pamasinda yo baba kufika paziba limoza (**at least 24 hours**) apo ndiko kuti bandamufumyani muchipatala?	YES (1)<br>NO (0)<br>DON'T KNOW (96)	

D29	***INTERVIEWER***: *"nizunulenge mautebeti wavyaumoyo yakupamabana pamba, ndipo nkukhumba kumanya para mukapokerera mautebeti agha panji yayi apo imwe makaba munyengo yo baba*			
		RECEIVED (1)	DID NOT RECEIVE (0)	DON'T KNOW (96)
	A Pathumbo yanosono yawumaliro kumasinda uku, Kasi bakamutumburani (kurepura pandumba nafufumiyamo mwana notimaso).	☐	☐	☐
	B Pobaba kwaumaliro kuasinda uku, bakubikanimo ndopa zinyakheso?	☐	☐	☐
	C Munkwara/nakubika majimumizipe (IV dilipu)	☐	☐	☐
	D Chisambiszgo umo tokonkheskera mwana	☐	☐	☐

	E	Katundu wakuvwira kujumphiska mphapo/chisambizgo pakureka kutumbirizga bana	☐	☐	☐
	F	Chisambizgo pakuphrererera mwana yura wababika sono	☐	☐	☐

D30	*INTERVIEWER:* panyengo iyi, nimufumbaninge pamasuzgo agho bamama bakusangika nagho mu vipatala panyungo yobaba. Para nazunura suzgo limoza limoza, nkhuromba kuti muyowoye para suzgo ilo namweso mukasangana nalo panyengo yobaba kura kuchupatala, para nitheura, munipharire kuti suzgo ilo likaba lirara panji likaba lichoko				
		MAJOR PROBLEM (2)	MINOR PROBLEM (1)	NO PROBLEM (0)	UNDECIDED (96)
	A MUTUNDU WAMINKWARA BAKUPOKELERA MUNYENGO YOBABA	☐	☐	☐	☐
	B NCHINDO IZO BATEBTEI BAMIVIPATALA BAKULONGOZGA	☐	☐	☐	☐
	C KUKUBA KWAKUBISIKA MAKORA UKO BOBABISKIRA	☐	☐	☐	☐
	D UDONGO WACHIPATALA	☐	☐	☐	☐
					After completing the facility-based delivery section, continue to MODULE E.

HOME DELIVERIES

No.	Question	Potential responses	Skip
D31	Mbanjani chomene mene abo baka gamura kuti imwe mubabile panyumba?	YOURSELF (1)<br>HUSBAND/PARTNER (2)<br>MOTHER/MOTHER-IN-LAW (3)<br>AUNTIE (4)<br>OTHER FAMILY MEMBER (5)<br>FRIEND (6)<br>OTHER (SPECIFY) (7):	
D32	Nchifukwa uli chikuru icho imwe mukababila kuchipatala yayi?<br><br>*Select all that apply.*	COST TOO MUCH (1)<br>FACILITY NOT OPEN (2)<br>TOO FAR/NO TRANSPORTATION (3)<br>POOR QUALITY SERVICE/DON'T TRUST (4)<br>NO FEMALE HEALTH PROVIDER (5)<br>HUSBAND/FAMILY DIDN'T ALLOW (6)<br>SHORT LABOR (7)<br>BABY CLOTHES (8)<br>CDK (9)<br>NO MOTHERS SHELTER (10)<br>NOT NECESSARY (11)<br>NOT CUSTOMARY (12)<br>OTHER (SPECIFY) (13):	
D33	Kasi mukaruta kuchipatara kuti bakamuwonani umoyo umo mulili/panji kupereka mwana kuti bakamuwon umoyo wakhe pepapo zuba limoza lindapite (24 hours) kufuma nyengo iyo mwababilapo?	YES (1)<br>NO (0)<br>DON'T KNOW (96)	

SURVEY ID

MODULE E: SPENDING AND SAVINGS

INTERVIEWER: "sono nimufumbaninge pandalama izo imwe mukasebezeska panthumbo na mphapo yinu. Muganizile kumasind ndalama izo mukasebezeska kukwaskana na zanthumbo yinu na uvyazi pamphapo yamwana wasono sono kumasinda uku, naumo imwe mukanozgekera ndlama"

E1					
	INTERVIEWER: "Sono tunkhumba kuti tidumbirane paunandi wandala izo zili kusebezeske pa pmahpo wa mwana wasoso sono kumasinda uku. Muganizire pavithu nga ni ivo mukaguranga kunozgekere mphapo yamwa, za ndalama ya mendero kukuma kunyumba kuruta kuchipatala no weraso, panji kuruta kunyumba uko imwe muka babila. Mwanozgeka tiyambe?"				
	Mukuganizira kuti mukasebezeska zingati ndalama pa:		AMOUNT (KWACHA)	NONE (0)	DON'T KNOW (96)
	ponozgekera:				
	A	KATUTU (Kusazgirak na vobabiskirat, magulovu, nyreti, mapulastic, tuneza, munkwala wosukirako, navinyakhe.)			
	B	VYOVWALA VYAMWANA/BULANGETI			
	Pa mendero:				
	C	Mendero panji tilansipoti kupita no wera (para mukababila panyumba pinu nubiko 0)			
	D	KUCHIBANDA ODI CHA BAMAMA ABO BALI PAKATI /MAGONERO APO MUKALINDIZGANGA KUTI MUBABE			
	Panyengo yobaba:				
	E	BAZAMBA PANJI BATEBETI/IZO BAKAMULIPILISKA KUCHIPATALA			
	F	TUNYAKKHE TOLIPILA TODOKOTODOKO TWAPAMPHEPETE			
	G	TIPS			
	H	MALIPIRO GHA NYAKHE AYO MUKALIPIRA MUNDALAMA CHA KWENI MUVINTHU (sinthani mundalama)			

SURVEY ID

	I	MINKWARA			
	J	CHIPIMO			
	K	MALIPIRO GHANYAKE			

NO.	QUESTION	POTENTIAL RESPONSES	SKIP
E2	Kasi mukasungako ndalama pamphepete ponozekera mphapo yamwana uyo makalekerazga kubaba soso sono kumasinda uku?	YES (1)<br>NO (0)<br>DON'T KNOW (96)	If (0) or (96), skip to E8
E3	Kasi mukuwona kuti ndalama izo mukasungu zikaba zokukwanira makora (kunozgekera, zamendero, nai mukatebeseska pazuba lakuphokwa?	YES (1)<br>NO (0)<br>DON'T KNOW (96)	
E4	Kasi izo ndalama mukasunganga, mukasungilanga nkhu?	AT YOUR HOME (1)<br>AT A FRIEND OR FAMILY MEMBER'S HOME (2)<br>IN A BANK ACCOUNT (3)<br>OTHER (SPECIFY) (4):	
E5	Kasi pakabako muthu waliyose pankhomo pinu (panji barumi binu) uyo wakaziwona nakukorapo pandarama izo mukasunganga?	YES (1)<br>NO (0)<br>DON'T KNOW (96)	
E6	Kasi muyamba kusaka ndalama izi ndiko kuti muli napakati miyezi yilinga?	[ ][ ] WEEKS<br>[ ][ ] MONTHS	
E7	Mbanjani banyakhe, para bangabapo Who else, abo bakamovwiraniko pa szgiapo kusanga ndalamo zapauvyazi (kunozgekera kose, pakendero, na po babay)?<br><br>*(Select all that apply)*	HUSBAND/PARTNER (1)<br>YOUR CHILDREN (2)<br>PARENT/GRANDPARENT (3)<br>OTHER FAMILY MEMBER (4)<br>FRIEND (5)<br>AUNTIE (6)<br>NO ONE (7)<br>OTHER (SPECIFY) (8):	
E8	Kasi nchakuzirwa munthowa wuri kusunga ndala kunozgekera kuphokwa?	NOT IMPORTANT (1)<br>SLIGHTLY IMPORTANT (2)<br>MODERATELY IMPORTANT (3)<br>IMPORTANT (4)<br>VERY IMPORTANT (5)	
E9	Kasi muli sungapo ndalama ku akaunti kubanki?	YES (1)<br>NO (0)	

		DON'T KNOW (96)	
E10	Kusi muli tumapo ndalama kusesezeska lamya yapamawoko ("mucizung bakuti mobile money")?	YES (1)<br>NO (0)<br>DON'T KNOW (96)	In (0) or (96), skip to module F
E11	Kasi mbanjani bauth ubo mukatumilako ndaramo kutebeteska lamya yapamawoko ("mobile money")?	HUSBAND/PARTNER (1)<br>YOUR CHILDREN (2)<br>PARENT/GRANDPARENT (3)<br>OTHER FAMILY MEMBER (4)<br>FRIEND (5)<br>AUNTIE (6)<br>OTHER (SPECIFY) (7):	

MODULE F. POST-NATAL CARE

INTERVIEWER: "sono nkhukhumba kuti nimufumbani para muli rutako kuchipimo kuti bakamuwonani imwe pamoza namwana kuchipatala kufuma pazuba ilo mukaphokwera."

NO.	QUESTION	POTENTIAL RESPONSES	SKIP
F1	Kasi muli kurutako kuchipatala chirichose kuti mukapimike pa vilivose kufuma pasiku mukaphokwera malinga ndiko kuti pajumphapo zuba limoza **(first 24 hours**)?	YES (1)<br>NO (0)<br>DON'T KNOW (96)	If (0) or (96) skip to F6
F2	Kasi muli kurutako kuchipatala chirichose kuti mukapimike pakati pajumphapo mazuba ghatatu(3days) kufuma pasiku mukaphokwera?	YES (1)<br>NO (0)<br>DON'T KNOW (96)	
F3	Kasi muli kurutako kuchipatala chirichose kuti mukapimike pakati pajumphapo mazuba kuyambira ghankhondi na gha bili(7days) kufika pamazuba khumi limoza na mazuba ghanayi(14days) kufuma pasiku mukaphokwera?	YES (1)<br>NO (0)<br>DON'T KNOW (96)	
F4	Kasi muli kurutako kuchipatala chirichose kuti mukapimike pakati pajumphapo masabata ghankhondi na yimoza pahanya(6weeks) kufuma pasiku mukaphokwera?	YES (1)<br>NO (0)<br>DON'T KNOW (96)	

F5	*INTERVIEWER:* "panyengo yino yasono, nimufumbaninge pamasuzgo yanyengo nanyengo agho bawomama bakusangikana nagho para ba phokwa sono barutaso Kuchipimo.Para nazunura suzgo limoza limoza, nkhuromba kuti muyowoye para suzgo ilo namweso mukasangikana nalo panyeko mukautako kuchipimo pamanyuma yakuphokwa, para nitheura, munipharire kuti suzgo ilo likaba lirara

SURVEY ID

	panji likaba lichoko					
			MAJOR PROBLEM (2)	MINOR PROBLEM (1)	NO PROBLEM (0)	UNDECIDED (96)
	A	NYENGO MUKALINDIZGANGA KUTI BAMUWONANI	☐	☐	☐	☐
	B	NTHOWA IYO MUKADUMBISKIRANAMO PAMASUZGO PANJI VYAKUMUTIMA PA VA NTHUMBO YINU	☐	☐	☐	☐
	C	KURONGOSORA MUKAPOKERA PASUZGO ILO NA WOVWIRI MUKAPOKERA	☐	☐	☐	☐
	D	KUBISIKA APO BAKAMUPIMANINGE KUTI BANJI BALEKE KUTAMBWAKO	☐	☐	☐	☐
	E	KUBISIKA KU BANYAKHE BANTHU BALEKU KUPURIKAKO IVO IMWE MUKADUMBIRANANA NA BATEBETI BEKUCHIPTALA	☐	☐	☐	☐
	F	UDONGO WACHIPATALA	☐	☐	☐	☐
	G	UMO IBO BATEMBETI BOWONA PA VAUMOYO KU CHIPTARA BAKAMUPOKELELELANI	☐	☐	☐	☐
	H	NDALAMA ISO ZIKANKHUMBIKANGA PANJI IZO MUKALIPIRA KUTI BAMUTHANGATANI	☐	☐	☐	☐

F6	Mazuba ghano, kasi mukutebetekako nthowa yoli yose kuchelweska kuba na pakati mwalibiro or panji kutumbirizga kamwana?	**YES, MODERN METHOD (1)**<br>**YES, TRADITIONAL METHOD (2)**<br>**NO (0)**<br>**N/A, CURRENTLY PREGNANT (3)**<br>**DON'T KNOW (96)**	
F7	**INSTRUCTIONS:** Look back to question **B27** – Kasi uyo mwana wakababika sono sono wachali ngwamoyo?<br><br>*Confirm with respondent.*	YES (1)<br>NO (0)<br>DON'T KNOW (96)	If (0) or (96) skip to F15
F8	Kasi muchali muwokheska na bele mwana uyu mukababa mphapo yasono sono kumasinda uku?	YES (1)<br>NO (0)<br>DON'T KNOW (96)	If (0) or (96) skip to F10
F9	Kasi panyengo yino yasono, mwna uyo mukupako vakurya vinyakhe Kuzgirapo mukaka wakubere nau munkwara?	YES (1)<br>NO (0)<br>DON'T KNOW (96)	
F10	Pamasabata<br>yabiri (2weeks) yajumphapo kumanyuma uku, Kasi mwana mulirutapo nayo kuchipatala pavifukwa vili vose kuti wakathangatike vaumoyo	YES (1)<br>NO (0)<br>DON'T KNOW (96)	If (0) or (96), skip to F12
F11	Nimalo wuri agho imwe mudakha	HEALTH CARE CENTER (1)	

	kuperekaka mwana kuti bakamuthangate pa vyaumoyo?	HOSPITAL (2)<br>PHARMACY (3)<br>TRADITIONAL HEALER (4)<br>OTHER (SPECIFY) (5):	
F12	Kasi mwana winu wlilasikapo nyeleti yili yose yakatemera?	YES (1)<br>NO (0)<br>DON'T KNOW (96)	If (0) or (96), skip to F15

F13	**INSTRUCTIONS:** Based on **D1**, calculate child's age.<br><br>*Specify unit of response.*				
	**INSTRUCTIONS:** Ask to see the child's vaccination card. If available, use card to confirm the vaccines received and mark below. If card is unavailable, ask mother which vaccines the child has received. **BASED ON CALCULATED AGE FROM F13**, ask only about **AGE APPROPRIATE** vaccines.				
F14	Confirm you have the child's vaccine card in-hand.	YES (1)<br>NO (0)			
		CONFIRMED BY VACCINE CARD		CONFIRMED BY MOTHER	
		RECEIVED	NOT RECEIVED	RECEIVED	NOT RECEIVED
	**Wakati babika waka, Kasi mwana winu wkapokera katemera uyu?**				
	A BCG	☐	☐	☐	☐
	B Polio (OPV-0)	☐	☐	☐	☐
	**Kasi mwana winu wakapokra katemra uyu pakati paitapo masabat ghankhondi nalimoza (6-week vaccines)?**				
	C Polio (OPV-1)	☐	☐	☐	☐
	D DTP-HepB-Hib-1	☐	☐	☐	☐
	E Pneumococcal (PCV)	☐	☐	☐	☐
	F Rotavirus	☐	☐	☐	☐
	**Kasi mwana winu wakapokra katemra uyu pakati pajumpapo masabata Khumi limoza (10-week vaccines)?**				
	G Polio (OPV-2)	☐	☐	☐	☐
	H DTP-HepB-Hib-2	☐	☐	☐	☐
	I Pneumococcal (PCV)	☐	☐	☐	☐
	J Rotavirus	☐	☐	☐	☐
	**Kasi mwana winu wakapokra katemra uyu pakati paijumphapo masabata khumi lomoza namasabata ghanayi (14-week vaccines)?**				
	K Polio (OPV-3)	☐	☐	☐	☐
	L DTP-HepB-Hib-3	☐	☐	☐	☐
	M Pneumococcal (PCV)	☐	☐	☐	☐

Uyu wakufumba: *"*Mafu yokokhapo, imwe mungazgora para yamukondwereskani. *Yanykhe mungazgora paramupenga kweni yanyakhe mungazgora yayi para mureka kupenja "*			
F15	Kasi mulipimkapo matenda yaka doyo ka HIV apo mukaba nanthumbo yasosno sono	YES (1)<br>NO (0)<br>PREFER NOT TO ANSWER (2)<br>DON'T KNOW (96)	

	kumasinda uku?		
F16	Kabiro kunu pankhani yakadoyo ka HIV/AIDS yiri uli?	INFECTED (1)<br>NOT-INFECTED (2)<br>PREFER NOT TO ANSWER (3)<br>DON'T KNOW (96)	If (2), (3), or (96), skip to F23
F17	Kasi mukamwanga mapilisi gha ma ARVs apo mukaba nathumbo ysonosono kumanyuma uku?	YES (1)<br>NO (0)<br>DON'T KNOW (96)	
F18	**INSTRUCTIONS:** Refer back to question **B27-28**. Did the respondent's baby survive beyond the day of birth?<br><br>*Confirm with respondent.*	YES (1)<br>NO (0)<br>DON'T KNOW (96)	If (0) or (96), skip to Module G
F19	Kasi mwana winu wakamwanga mukwara wama ARVs pamasabata ghankhondi nayimoza pachanya (6 weeks) yakubabika kwake?	YES (1)<br>YES, BUT BABY DIED BEFORE 6 WEEKS OF AGE (2)<br>NO (0)<br>DON'T KNOW (96)	
F20	Kasi mwana winuwakapimika matenda yakadoyo ka HIV?	YES (1)<br>NO (0)<br>DON'T KNOW (96)	If (0) or (96), skip to F23
F21	Kasi mwana winu ndko kuti wali nama sabata ghaligha gha ku babika panyengo iyo imwe mukamupiskanga?<br><br>*Round to nearest full number.*		
F22	Kasi nvivihi ivo vitumbukamo apo bakati mupima mwana?	INFECTED (1)<br>NOT INFECTED (2)<br>PREFER NOT TO ANSWER (3)<br>DON'T KNOW (96)	

F23	Pamazuba ghatatu ghazumhapo kumasinda uku, kasi imwe panji waliyose wpanyumba yinu malinga walinjumpha vilimika nkumu navinkhondi(15years) waliyezgapo kuseberaku nayo mwana munthowa nga ni izi zili apa<br><br>*Select all that apply*				
			YES (1)	NO (0)	DON'T KNOW (96)
	A	KUMUBAGHIRA PANJI KUMUBERENGRERA MWA NABUKU NAKUTAMBWA NAYO VITHUZITHUZI MUMABUKU	☐	☐	☐
	B	KUMUPHARIRA MWANA VISIMI	☐	☐	☐
	C	KUMUYIMBIRA A NYIMBO NAKUMURURUTIRA MWANA	☐	☐	☐
	D	KUMUNYAMURAKO MWANA, KUMUFUMYAKO PAWARO YA NYUMBA NAKUMUTANDAZGAKO PANJI KWENDAKO YENDAKO NAYO MUCHIKAYA	☐	☐	☐

	E	KUCHEMAKO MAZINA YABANTHU BANYAKHE, KUYEZGERERA KUPENDA NAKULELEMBALEMBA PAMOZA NA MWANA	☐	☐	☐

F24	*INTERVIEWER: "For the following questions, please respond only if you feel comfortable doing so. Your response is optional. I am going to read you a list of problems. Please tell me how often each of these problems has happened to you in the PAST TWO WEEKS: never, once in a while, more than half the time, or almost always."*					
			NEVER (0)	ONCE IN A WHILE (1)	MORE THAN HALF THE TIME (2)	ALMOST ALWAYS (3)
	A	MA SABATA YABILI YAKUMASINDA, KIKABA OKWIYA PANYAKHE KWAMBULA KUKONDWA	☐	☐	☐	☐
	B	MA SABATA YABILI YAKUMASINDA, NIKABA WAMBULA KUKHUMBA PA VINTHU (VANCHITO VINAVACHE, NCHITHO WAKA, BANTHU)	☐	☐	☐	☐
	C	MA SABATA YABILI YAKUMASINDA, NANGU LILA	☐	☐	☐	☐
	D	MA SABATA YABILI YAKUMASINDA, NIKABA OPHUKWA	☐	☐	☐	☐

F25	*INTERVIEWER: "Now I am going to read you a list of things that you may have experienced. Please tell me how often each of these events have happened to you in the past two weeks: never, once in a while, a few times, or many times. Again, please respond only if you feel comfortable doing so. Your response is optional."*						
			NEVER (0)	ONCE (1)	A FEW TIMES (2)	MANY TIMES (3)	N/A (4)
	A	MA SABATA YABILI YAKUMASINDA, NIPANYENGO ZILINGA KASI ASWENI BINU, ABWENZI BINU ANALUME BAKAKU SUNKHANIPO PANYAKHE KUKUMENYANIPO MBAMA?	☐	☐	☐	☐	☐
	B	MA SABATA YABILI YAKUMASINDA, NIPANYENGO ZILINGA KASI ASWENI BINU, ABWEZI BINU ANALUME BAKAKUCHAYANI CHIBAKHA, KUKUDOSANI DOSANI, KUKUCHAYANI, KUKUKOLANI PAMKOSI PANYAKHE KUMUSHOKANIPO?	☐	☐	☐	☐	☐

MODULE G. LAST PREGNANCY

INTERVIEWER: *"Sono nimufumbaninge mafumbo pa nthumbo iyo imwe mukalekelezgerapo kuba nayo kufika napanyenge iyo imwe mukababila panji kuwombokwa. Muwezgere maghanoghano kumasinda*

nakuganiza panyengo apo imwe mukamanyira ku sono mwankhara napathupi, kweneso muganizire umo mukasungikiranga na panyengo yachipimo apo imwe mukabananthumbo?"

NO.	QUESTION	POTENTIAL RESPONSES			SKIP
**Interviewer**: *Ask to see if antenatal care card is available for the woman's last pregnancy that led to a delivery and confirm information provided by respondent.*					
G1	Did the woman provide you with her antenatal card?	YES (1)<br>NO (0)<br>DON'T KNOW (96)			
G2	Mukarautako miku yingati kuchipimo Kuchipatara panyengo iyo mukaba napathupi pakulekerezgere kumasinda uku?	NONE (0)<br>ONE TIME (1)<br>TWO TIMES (2)<br>THREE TIMES (3)<br>FOUR TIMES (4)<br>MORE THAN FOUR TIMES (5)			If (0) skip to End of Survey
G3	Apo mukaruta kuchipimo, kasi mukadumbisiskanapo pamakani nga ni agha?	YES (1)	NO (0)	YES (1)	
	A Kumalo uko mwamukuphokwera?	☐	☐	☐	
	B Ivo mungachita para pakati pauka mwama buchibuchi?	☐	☐	☐	
	C Kusaungako Kandalama pamphepete kovwirako panyengo yapakati napa nyengo yakuphokwa para yafika?	☐	☐	☐	
	D dazi ilo baka ilo ibo bakaphara k aba la kuphokwarapo?	☐	☐	☐	
G4	Kasi mukukumbuka zuba ilo mukampharirika kuti ndilo zuba mangazobabilapo?	YES (1)<br>NO (0)<br>DON'T KNOW (96)			If (0), skip to G6
G5	Nga imwe mwabenecho nidazi uli ilo makaganiziranga kuti munga mungaphokwerapo panji? (dazi mwezi Chilimika)<br><br>*If EDD is on ANC card, copy it from card. If no card and date not know, enter 15th*	___ ___ ___ ___ ___ ___ ___ ___<br>D D M M Y Y Y Y			
G6	Mukaba napakati miyezi panji masaba yalinga apomukaruko kuchipimo muku **WAKWAMBA**?<br><br>*Please specify unit of response.*	☐☐<br>WEEKS			

SURVEY ID

		MONTHS	

INTERVIEWER: *"sono tafika kuwumaliro wachisambizgo chithuy. Nawonga kuti mwazgora mafumbo wose. Panji mungabako na vyakusazghirapo vinyakhe vapadera ivo mukhumba kusazhirapo?"*

G7	Would you be willing to have someone come back and follow up on a few questions from the survey in the next couple of days?	YES (1)<br>NO (0)	

COMMENTS:

END OF SURVEY

INSTRUMENT REVIEW

Enumerator Initials:	
Date (DD/MM/YYYY)	
Supervisor Initials:	
Date (DD/MM/YYYY)	

Data Entry Initials:	
Date (DD/MM/YYYY)	
Supervisor Initials:	
Date (DD/MM/YYYY)	
