# Supplementary material for: Characteristics of maternity waiting homes and the women who use them: Findings from a baseline cross-sectional household survey among SMGL-supported districts in Zambia
Source: PLoS One. 2018 Dec 31;13(12):e0209815. doi: 10.1371/journal.pone.0209815 (PMC6312364; doi:10.1371/journal.pone.0209815)
Supplement: S5 File — Household Survey Baseline Impact Evaluation–Nyanja. (PDF) [file pone.0209815.s005.pdf]

SURVEY ID | | | | | | | | | |

**Instrument ID:**

# The MAHMAZ Project
# Baseline Impact Evaluation – Household Survey **NYANJA**

**Target Audience:**

*Women who have delivered a child in the last 12 months, who are ≥ 15 years of age, and who live within the study facility catchment areas*

## SHORT SCREEN

| | | | |
|---|---|---|---|
| SS1 | Kodi ndi angati azikazi azaka khumi ndi zisanu (15) kufika pa zaka makhumi anayi zisanu ndi zinayi (49) amakhala pakhomo lino, kuphatikizapo ndi amene anamwalira kapena kuchokapo mu miyezi khumi ndi ziwiri zapitazi?<br><br>*Write down the number of women.* | | If none, thank person and move to next household. |
| SS2 | Kodi kuli akazi anabereka mwana mu myezi khumi ndi ziwili (12) yapita (chaka chimodzi), kosaganizira zolondola pa umoyo wa amayi kapena mwana? | YES (1)<br>NO (0)<br>DON'T KNOW (96) | If (0) or (96), thank person and move to next household |
| SS3 | Ngati mkazi wabanja lino palibe, kodi mungayanke mafunso ya mimba ndi ubeleki wake? | YES (1)<br>NO (0)<br>DON'T KNOW (96) | |
| **INTERVIEWER: IF YOU HAVE ANSWERED YES TO SS1 AND YES TO SS2, THEN PROCEED WITH THE INFORMED CONSENTING PROCESS. PLACE THE UNIQUE ID STICKER ON THE INSTRUMENT AND ON THE HOUSEHOLD CONSENT FORM A.** | | | |
| | ***Confirm consent was granted***<br><br>*Draw a check mark if consent was granted.* | | |
| **IF CONSENT WAS GRANTED, PLACE A SECOND UNIQUE ID STICKER ON THE PAPER VERSION OF THE INSTRUMENT.** | | | |

SURVEY ID | | | | | | | | | |

# MODULE A. LOCATION

**INSTRUCTIONS:** Complete before administering the rest of the survey

| NO. | FIELD | CODE | RESPONSE |
|---|---|---|---|
| A1 | Province | EASTERN (1)<br>SOUTHERN (2)<br>LUAPULA (3) | |
| A2 | District | CHOMA (1)<br>KALOMO (2)<br>PEMBA (3)<br>LUNDAZI (4)<br>NYIMBA (5)<br>MANSA (6)<br>CHEMBE (7) | |
| A3 | Health Facility Catchment Area | CHOMA DISTRICT<br>CHOMA GENERAL (801001)<br>MANGUNZA (801019)<br>MACHA MISSION (801002)<br>MASUKU MISSION (801021)<br>MBABALA (801022)<br>MOCHIPAPA (801023)<br>SIMAKUTU (801043)<br>KALOMO DISTRICT<br>CHIFUSA HC (804023)<br>CHILALA HC (804024)<br>DIMBWE HC (804019)<br>HABULILE HC (804032)<br>KALOMO DISTRICT HOSPITAL (804002)<br>KANCHELE HC (804014)<br>MAWAYA HC (804034)<br>MOONDE HP (804042)<br>MUKWELA HC (804020)<br>SIACHITEMA HC (804013)<br>PEMBA DISTRICT<br>JEMBO (801413)<br>MUZOKA (801419)<br>NYIMBA DISTRICT<br>CHIPEMBE RHC (307010)<br>HOFMEYR ZONAL HC (307011)<br>KACHOLOLA RHC (307012)<br>MKOPEKA RHC (307016)<br>NYIMBA DISTRICT HOSPITAL (307001)<br>MANSA DISTRICT<br>FIMPULU (403017)<br>KABUNDA (403018)<br>LUBENDE (403041)<br>MANO (403026)<br>MANSA GENERAL HOSPITAL (403001)<br>MIBENGE (403029)<br>MUSAILA (403030)<br>MUTITI (403031)<br>MUWANGUNI (403032)<br>CHEMBE DISTRICT | |

| | | | |
|---|---|---|---|
| | | KUNDAMFUMU (403023)<br>LUKOLA (403037)<br>LUNDAZI DISTRICT<br>CHIKOMENI (405026)<br>KAMSARO (305034)<br>KAPICHILA (305023)<br>LUKWISIZI (305040)<br>LUNDAZI HOSPITAL (305032)<br>LUSUNTHA (305021)<br>MWASE LUNDAZI ZONAL (305011)<br>NKHANGA (305046)<br>NYANGWE (305020)<br>PHIKAMALAZA (305031)<br>ZUMWANDA (305024) | |
| A4 | Village Name<br><br>*Write in the name of the village.* | | |
| **GPS COORDINATES, TAKE 1** | | | |
| A5 | Latitude | | |
| A6 | Longitude | | |
| **GPS COORDINATES, TAKE 2** | | | |
| A7 | Latitude (decimal format) | | |
| A8 | Longitude (decimal format) | | |
| A9 | Date of Interview (DD/MM/YYYY) | | |
| A10 | Start time of interview<br>(24:00 format) | | |
| A11 | ***Confirm consent was granted***<br><br>*Draw a check mark if consent was granted.* | | |

SURVEY ID [ ][ ][ ][ ][ ][ ][ ][ ][ ]

## MODULE B. HOUSEHOLD ENUMERATION

**INSTRUCTIONS:** Confirm that the person who you are speaking with is the head of the household or the head woman of the household.

**INTERVIEWER**: *"Tsopano ndiza kufunsani mafunso okhudza inu ndi anthu amene mukhala nao panyumba ino. Kulingana ndi funso ili, titaunzire kuti banja inanthu amene akhala pamodzi, kuphika pamodzi komanso kudyela limonzi ndiponso amayang'anila pa munthu umadzi kukhala mutu wa banjalo."*

| NO. | QUESTION | POTENTIAL RESPONSES | SKIP |
|---|---|---|---|
| B1 | Kufikira pachikumukiro cha tsiku lanu laku badwa lotsiriza, munali ndi zaka zingati zakubadwa?<br><br>*Unit of response in years.* | | |
| B2 | Kodi unapitako ku sukulu? | YES (1)<br>NO (0)<br>DON'T KNOW (96) | If (0) or (96), skip to B4 |
| B3 | Kodi ni grade bwanji yapamwamba yamene munasiliza?<br><br>*If <1 year completed, write down 00. If >12 years completed, write down 13.* | [ ][ ]<br><br>DON'T KNOW (96) | |
| B4 | Kodi chipembedzo chanu nichiti? | CATHOLIC (1)<br>PROTESTANT (2)<br>MUSLIM (3)<br>OTHER (SPECIFY) (4) | |
| B5 | Ndimwe atundu bwanji? | | |
| B6 | Kodi udindo wazachikwati wanu uli bwanji? | MARRIED/COHABITING (1)<br>DIVORCED (2)<br>SEPARATED (3)<br>WIDOWED (4)<br>NEVER-MARRIED (5) | If (2), (3), (4), or (5), skip to B8 |
| B7 | *If respondent is the male head of household:*<br>Ni chiwerengelo bwanji cha akazi amene muli nawo?<br><br>*If respondent is NOT male head of household:*<br>Ni chiwerengelo bwanji cha akazi chamene amuna a banja lino ali nawo? | | |
| B8 | Kodi ni ambiri bwanji anyamata ndi atsikana osakwana zaka zisanu (5) amene ankala pakhomo lanu kawirikawiri? | [ ][ ] BOYS<br><br>[ ][ ] GIRLS | |

| | | | |
|---|---|---|---|
| | *Include children who are in boarding school at the moment. If none, write down 00.* | | |
| B9 | Kodi ni ambiri bwanji anyamata ndi atsikana azaka zapakati pa zisanu (5) ndi khumi ndi zinayi (14) amene ankala pakhomo lanu kawirikawiri?<br><br>*Include children who are in boarding school at the moment. If none, write down 00.* | BOYS<br>GIRLS | |
| B10 | Kuikilapo ndi inu, kodi ni ambiri bwanji amuna ndi akazi azaka zapakati pa khumi ndi zisanu (15) ndi makhumi anayi zisanu ndi zinayi (49) amene akhala pakhomo lanu kawirikawiri? | MEN<br>WOMEN | |
| | Kuikilapo ndi inu, kodi ni ambiri bwanji amuna ndi akazi azaka zapakati pa khumi azisanu (15) ndi makhumi anayi zisanu ndi zinayi (49) amene ankala pakhomo lanu kawirikawiri, kuphatikizapo amene ana mwalila mu myezi yokwana khumi ndi ziwili (12) zapita? | MEN<br>WOMEN | |
| B11 | Kuikilapo ndi inu, kodi ni ambiri bwanji amuna ndi akazi azaka zapakati pa makumi asanu (50) ndi pa nkhumi asanu ndi limodzi ndi zinayi (64) amene akhala pakhomo lanu kawirikawiri? | MEN<br>WOMEN | |
| B12 | Kuikilapo ndi inu, kodi ni ambiri bwanji amuna ndi akazi azaka makhumi asanu ndi limodzi ndi zinai (65) kapena kwambiri amene ankala pakhomo lanu? | MEN<br>WOMEN | |
| **INSTRUCTIONS:** Count and record the total number (B8 to B12) of household members. | | | |
| B13 | Tsimikizani ndi woyankha: Kotero kuli okwana (chiwerengero) anthu amu banja lanu? | YES (1)<br>NO (0) | |

SURVEY ID | | | | | | | | | |

**INSTRUCTIONS:** Ask the respondent to list the names of all women aged 15-49 in the household, including those who passed away in the last 12 months (1 year). Emphasize that you are also looking for information on individuals who have passed away in the last 12 months (1 year). Fill out column A with all names provided, and then continue to answer B-F for each person before selecting a respondent.

**INTERVIEWER:** *"Tsopano ine ndikufuna inu kuti mundiuzeko mazina a akazi onse amene kawirikawiri amakhala pakhomo lino ammene ali ndizaka zapakati pa zaka khumi ndi zisanu (15) ndi zaka makhumi anayi zisanu ndi zinayi (49). Nipempa mundiuzenso mazina ya akazi omwe ana mwarira myezi zapita zokwana khumi ndi ziwili (12) (chaka chimodzi). "*

**TABLE 1.** ROSTER OF WOMEN AGED 15-49 YEARS

| | A.Nipempa muniuze madzina loyamba ndi zaka ya akazi onse a zaka zapakati pa khumi ndi zisanu (15) ndi azaka makhumi anayi zisanu ndi zinayi (49) amene nthawi zambiri amakhala pakhomo iyi kwa masiku anai a musondo umodzi, kuphatikizapo amene ana mwalila mu myezi yokwana khumi ndi ziwili (12) zapita.<br><br>Ensure the number includes those who would have been living there if they didn't pass away/move away in the past 12 months.<br>*The number of women in this list should be greater or equal to the number of women in* ***B10****.* | B. Mu myezi zapita khumi ndi ziwili (12), kodi anakhalapo ndipakati po pyola masabata makhumi atatu ndi asnu (35)?<br><br>YES (1)<br>NO (0)<br>DON'T KNOW (96)<br><br>*If (0) or (96), skip to next person.* | C. kodi (dzina) ali moyo?<br><br>YES (1)<br>NO/DON'T KNOW (0)<br><br>*If (1), skip to E.* | D. Kodi muli wokonzeka kapena wina wachekuyankha mafunso ya zamimba ya (*name)?<br><br>YES (1)<br>NO (0)<br><br>*If (0), skip to next person.* | E. Is (name) potentially eligible to take the survey?<br><br>*If* ***B=1*** *and (****IF APPLICABLE) D=1,*** *mark the box below.* | **AFTER ALL WOMEN HAVE BEEN LISTED, TO SELECT A RESPONDENT:**<br><br>1. Roll the die<br>2. From the 1st checked box in Column E, count up to the rolled number, beginning again at the 1st checked box if needed until number is reached<br>3. Roll the die again<br>4. From the checked box you landed on after the 1st roll, count up to the 2nd rolled number, beginning again at the 1st checked box if needed until the 2nd number is reached<br>5. Select this woman<br>6. If woman selected is ALIVE, proceed to **Question B24**<br>7. If woman selected is DECEASED, proceed to Proxy Household Survey |
|---|---|---|---|---|---|---|
| B14 | | | | | ☐ | |
| B15 | | | | | ☐ | |
| B16 | | | | | ☐ | |
| B17 | | | | | ☐ | |
| B18 | | | | | ☐ | |
| B19 | | | | | ☐ | |
| B20 | | | | | ☐ | |
| B21 | | | | | ☐ | |

SURVEY ID | | | | | | | | | |

| | | | | | | |
|---|---|---|---|---|---|---|
| B22 | | | | | ☐ | |
| B23 | | | | | ☐ | |

SURVEY ID | | | | | | | | | |

| NO. | QUESTION | POTENTIAL RESPONSES | SKIP |
|---|---|---|---|
| B23A | Kodi (*name) ali ndi zaka zokubadwa zingati?<br><br>*Input name* | \| \| \| | |
| B24 | Kodi (*name) alipo kuti angayanke mafunso?<br><br>*Input name* | YES (1)<br>NO (0)<br>DON'T KNOW (96) | If (1), skip to consent then proceed to B27 |
| B25 | Kodi ife tingakonze nthawi ina ya kubwera pamene iye adzakhala alipo? | YES (1)<br>NO (0)<br>DON'T KNOW (96) | If (0) or (96), resample from potentially eligible women in TABLE 1 |
| B26 | Were you able to reschedule another time? | YES (1)<br>NO (0) | If (0), resample from potentially eligible women in TABLE 1 |
| If you are unable to reschedule a time to come back and survey the sampled woman, go back to TABLE 1 and resample another potentially eligible woman. If you are re-visiting the household a subsequent time and the woman is now available, **proceed from Question B27**. | | | |

**INSTRUCTIONS:** Make sure to obtain consent or assent (if the sampled woman is 15, 16 or 17 years old – refer to B23A), including a signature, from the sampled woman. If the woman is not able to sign, please have the woman provide a thumbprint. These questions will determine whether or not the sampled woman is eligible to proceed to the full household survey. If she is ineligible, then re-sample from Roster Table 1. If there are no more potentially eligible women to sample from, thank the woman and move on to the next household. If she is eligible, proceed to Module C.

**STOP: MAKE SURE CONSENT OR ASSENT WAS OBTAINED FROM (NAME). PLACE A THIRD UNIQUE ID STICKER ON THE CONSENT FORM B – FOR THE ELIGBLE WOMAN.**

**INTERVIEWER:** *"Zikomo kwambili pa kutengako mbali pa kafukufuku wathu. Tsopano ine ndikuti ndikufunseni inu mafunso ya ubeleki wa mwana anabadwa posachedwapa."*

| NO. | QUESTION | POTENTIAL RESPONSES | SKIP |
|---|---|---|---|
| B27 | Kodi mwana wanu ali moyo?<br><br>*Soften the question by asking: Is your baby around?* | YES (1)<br>NO (0)<br>DON'T KNOW (96) | If (1), continue to Module C<br>If (96), skip to B29 |
| B28 | Kodi mwana yanu anamwalira liti? | BEFORE OR ON DAY OF DELIVERY (1)<br>WITHIN ONE MONTH AFTER DELIVERY (2)<br>OTHER (3) | If (2) or (3), continue to Module C |
| B29 | Kodi munabeleka mwana kamusanga kosakwana masiku yamene munali kuyembekezeleka? | YES (1)<br>NO (0)<br>DON'T KNOW (96) | If (0), continue to Module C<br>If (96), end and re-sample from Roster Table 1 |

| | | | |
|---|---|---|---|
| B30 | Kodi mwana anabadwa kutatsala ma sabata angati pa tsiku iomwe munali kuyenela kuchilirapo? | <= 3 WEEKS (1)<br>>3 WEEKS (2)<br>DON'T KNOW (96) | If (1), continue to Module C<br>If (2), end and re-sample from Roster Table 1<br>If 96, end and re-sample from Roster Table 1 |

## MODULE C. DEMOGRAPHICS

**INSTRUCTIONS:** After eligible respondent has been randomly sampled from all eligible respondents, proceed with the instrument. Ensure that the woman selected to proceed with the survey has delivered a child **within the last year**. This section is to get basic demographics on the household and the respondent.
***INTERVIEWER:*** *"Ine tsopano ndikuti ndikufunseni inu mafunso ena okhudza inuyo ndi banja lanu."*

| NO. | QUESTION | POTENTIAL RESPONSES | SKIP |
|---|---|---|---|
| C1 | Kodi ndinu mutu wabanja? | YES (1)<br>NO (0) | If (1), skip to C9 |
| C2 | Ni cibale cotani cilipo pakati pa imwe na wamutu wabanja? | SPOUSE (1)<br>CHILD (2)<br>GRANDCHILD (3)<br>NIECE (4)<br>AUNTIE/OTHER RELATIVE (5)<br>OTHER (SPECIFY) (6): | |
| C3 | Kodi munaphunzilako sukulu? | YES (1)<br>NO (0)<br>DON'T KNOW (96) | If (0) or (96), skip to C5 |
| C4 | Kodi maphunzilo yanu yanafika pati?<br><br>*If <1 year completed, write down 00. If >12 years completed, write down 13.* | DON'T KNOW (96) | |
| C5 | Kodi ndimwe wa chipembedzo cabwanji? | CATHOLIC (1)<br>PROTESTANT (2)<br>MUSLIM (3)<br>OTHER (SPECIFY) (4): | |
| C6 | Kodi ndinu amutundu bwanji? | | |
| C7 | Kodi ukwati wanu ni wotani? | MARRIED/COHABITING (1)<br>DIVORCED (2)<br>SEPARATED (3)<br>WIDOWED (4)<br>NEVER-MARRIED (5) | If (2), (3), (4) or (5), skip to C9 |

| | | | |
|---|---|---|---|
| C8 | Ndi chiwelengelo chotani cha azimai kuwonjeza cisumbali cymene amuna anu alinacho?<br><br>*If don't know, write down 96.* | | |
| C9 | Muna khalapo na mimba kangati? | | |
| C10 | Kodi munabalapo ana amoyo angati? | | |

***INTERVIEWER***: *"Tsopano tikambe za nyumbayanu."*

| NO. | QUESTION | POTENTIAL RESPONSES | SKIP |
|---|---|---|---|
| C11 | Kodi banja lanu **kawirikawiri** imatunga kuti manzi yamene mukumwa? | *PIPED WATER*<br>PIPED INTO DWELLING (1)<br>PIPED TO YARD/PLOT (2)<br>PUBLIC TAP/STANDPIPE (3)<br>TUBE WELL OR BOREHOLE (4)<br>*DUG WELL*<br>PROTECTED WELL (5)<br>UNPROTETED WELL (6)<br>*WATER FROM SPRING*<br>PROTECTED SPRING (7)<br>UNPROTECTED SPRING (8)<br>RAINWATER (9)<br>TANKER TRUCK (10)<br>CART WITH SMALL TANK (11)<br>SURFACE WATER (12)<br>(RIVER/DAM/LAKE/POND/STREAM/CANAL/<br>IRRIGATION CHANNEL)<br>BOTTLED WATER (13)<br>OTHER (PLEASE SPECIFY) (14): | If (13), skip to C14 |
| C12 | Madzi mutenga kuti? | IN OWN DWELLING (1)<br>IN OWN YARD/PLOT (2)<br>ELSEWHERE (3) | If (1) or (2), skip to C14 |
| C13 | Mumatenga mpindi zingati kupita ndi kubwela poka tenga madzi? | MINUTES (1):<br><br>DON'T KNOW (96) | |
| C14 | Kodi mumachita chilichonse kumadzi kuti yankale ambwino kumwa? | YES (1)<br>NO (0)<br>DON'T KNOW (96) | |
| C15 | Ndi cimbudzi cotani camene inu ndi banja lanu mugwiritsa nchito nthawi zambili? | *FLUSH OR POUR FLUSH TOILET*<br>FLUSH TO PIPED SEWER SYSTEM (1)<br>FLUSH TO SEPTIC TANK (2)<br>FLUSH TO PIT LATRINE (3)<br>FLUSH TO SOMEWHERE ELSE (4)<br>FLUSH, DON'T KNOW WHERE (5)<br>*PIT LATRINE*<br>VENTILATED IMPROVED PIT LATRINE (6)<br>PIT LATRINE WITH SLAB (7)<br>PIT LATRINE WITHOUT SLAB/OPEN PIT (8)<br>COMPOSTING TOILET (9)<br>BUCKET TOILET (10)<br>HANGING TOILET/HANGING LATRINE (11)<br>NO FACILITY/BUSH/FIELD (12)<br>OTHER (SPECIFY) (13): | |

SURVEY ID | | | | | | | | | |

| | | | |
|---|---|---|---|
| C16 | Kodi musebenzesa cimbudzi chimodzi ndi banja ena? | YES (1)<br>NO (0) | |

| C17 | Kodi banja lanu liri ndi mbali zotsatirazi *(item must be functioning usually):* | | **YES (1)** | **NO (0)** | **DON'T KNOW (96)** |
|---|---|---|---|---|---|
| | A | MAGESI | ☐ | ☐ | ☐ |
| | B | MAGESI YAPANGIDWA NDI ZUWA | ☐ | ☐ | ☐ |
| | C | MAGESI YA MUCHINI | ☐ | ☐ | ☐ |
| | D | NYALE YA MAFUTA YA PALAFINI | ☐ | ☐ | ☐ |
| | E | FILIJI | ☐ | ☐ | ☐ |
| | F | MICROWAVE | ☐ | ☐ | ☐ |
| | G | MBAULA YA MALASHA | ☐ | ☐ | ☐ |
| | H | MBAULA YA NKHUNI | ☐ | ☐ | ☐ |
| | I | CHITOFU CHA MAGESI | ☐ | ☐ | ☐ |
| | J | BEDI | ☐ | ☐ | ☐ |
| | K | MATILESI | ☐ | ☐ | ☐ |
| | L | MPANDO | ☐ | ☐ | ☐ |
| | M | THEBULO | ☐ | ☐ | ☐ |
| | N | KABATI | ☐ | ☐ | ☐ |
| | O | MIPANDO YA SOFA | ☐ | ☐ | ☐ |
| | P | KOLOKO | ☐ | ☐ | ☐ |
| | Q | FANI | ☐ | ☐ | ☐ |
| | R | MASHINI YOSOKELA | ☐ | ☐ | ☐ |
| | S | KOMBE YA UDZUZU | ☐ | ☐ | ☐ |
| | T | INTERNET | ☐ | ☐ | ☐ |
| | U | NKOLOKO YA PA MANJA | ☐ | ☐ | ☐ |
| | V | BUKU LA BANKI /SAVINGS ACCOUNT | ☐ | ☐ | ☐ |
| | W | KHASU LOLIMILA NDI NGOMBE | ☐ | ☐ | ☐ |
| | X | WILIBALA | ☐ | ☐ | ☐ |
| | Y | CHOGAILA NDI MANJA | ☐ | ☐ | ☐ |
| | Z | TALAKITA | ☐ | ☐ | ☐ |
| | AA | CHIGAYO | ☐ | ☐ | ☐ |
| | BB | FOSHOLO | ☐ | ☐ | ☐ |
| | CC | MACHETE | ☐ | ☐ | ☐ |

| | | | | |
|---|---|---|---|---|
| DD | PIKI / NKHWANGWA | ☐ | ☐ | ☐ |
| EE | MUCHINI WOPOMPA MADZI | ☐ | ☐ | ☐ |
| FF | MALO OLIMAPO | ☐ | ☐ | ☐ |
| GG | MITENGO YOBELEKA ZIPATSO | ☐ | ☐ | ☐ |
| HH | WAYALESI | ☐ | ☐ | ☐ |
| II | WAYALESI YA KANEMA | ☐ | ☐ | ☐ |
| JJ | TELEFONI YAPA THUMBA | ☐ | ☐ | ☐ |
| KK | TELEFONI YA MUNYUMBA | ☐ | ☐ | ☐ |
| LL | COMPUTER | ☐ | ☐ | ☐ |
| MM | CASSETTE PLAYER | ☐ | ☐ | ☐ |
| NN | VCR/DVD | ☐ | ☐ | ☐ |
| OO | NJINGA | ☐ | ☐ | ☐ |
| PP | MTHUTHUTHU/SKUTA | ☐ | ☐ | ☐ |
| QQ | KOCHIKALA | ☐ | ☐ | ☐ |
| RR | GALIMOTO YONYAMULILAMO ANTHU/ KATUNDU | ☐ | ☐ | ☐ |
| SS | BWATO LOYENDA NDI MPHAMVU ZA MUCHINI | ☐ | ☐ | ☐ |
| TT | BWATO LOYENDETSEWA NDI MPHAMVU ZA ANTHU | ☐ | ☐ | ☐ |

| | | | |
|---|---|---|---|
| C18 | Kodi pophika mumagwiritsa nchito ciani? | ELECTRICITY (1)<br>SOLAR POWER (2)<br>LIQUID PROPANE GAS (LPG) (3)<br>NATURAL GAS (4)<br>BIOGAS (5)<br>KEROSENE (6)<br>COAL, LIGNITE (7)<br>CHARCOAL (8)<br>WOOD (9)<br>STRAW/SHRUBS/GRASS (10)<br>AGRICULTURAL CROP (11)<br>ANIMAL DUNG (12)<br>NO FOOD COOKED IN HOUSEHOLD (13)<br>OTHER (SPECIFY) (14): | If (13), skip to C20 |
| C19 | Kodi muma phikira kuti pa nyumba pano? | IN THE HOUSE (1)<br>IN A SEPARATE BUILDING (2)<br>OUTDOORS (3)<br>OTHER (SPECIFY) (4): | |
| C20 | Kodi pansi panyumba yanuyi mpopangidwa ndi ciani? | NATURAL FLOOR<br>EARTH/SAND (1)<br>DUNG (2)<br>RUDIMENTARY FLOOR<br>WOOD PLANKS (3) | |

| | | | |
|---|---|---|---|
| | *OBSERVE THE FLOOR TO CONFIRM. (If more than one material, select the one that is "most" common)* | PALM/BAMBOO/REEDS (4)<br>FINISHED FLOOR<br>PARQUET/POLISHED WOOD (5)<br>VINYL (PVC) OR ASPHALT STRIPS (6)<br>CERAMIC/TERRAZZO TILES (7)<br>CONCRETE CEMENT (8)<br>CARPET (9)<br>OTHER (SPECIFY) (10): | |
| C21 | Kodi mtenje wa nyumba yanuyi ndiopangidwa ndi ciani?<br><br>*OBSERVE THE ROOF TO CONFIRM. (If more than one material, select the one that is "most" common)* | *NATURAL ROOFING*<br>NO ROOF (0)<br>THATCH/PALM LEAF (1)<br>*RUDIMENTARY ROOFING*<br>RUSTIC MAT (2)<br>PALM/BAMBOO (3)<br>WOOD PLANKS (4)<br>CARDBOARD (5)<br>*FINISHED ROOFING*<br>METAL/IRON SHEETS (6)<br>WOOD (7)<br>CALAMINE/CEMENT FIBRE (ASBESTOS) (8)<br>CERAMIC/HARVEY TILES (9)<br>CEMENT (10)<br>ROOFING SHINGLES (11)<br>MUD TILES (12)<br>OTHER (SPECIFY) (13): | |
| C22 | Kodi nyumbayi inapangidwa ndi ciani mu zipupa za kunja?<br><br>*OBSERVE THE WALLS TO CONFIRM. (If more than one material, select the one that is "most" common)* | *NATURAL WALLS*<br>NO WALLS (0)<br>CANE/PALM/TRUNKS (1)<br>MUD (2)<br>*RUDIMENTARY WALLS*<br>BAMBOO/POLE WITH MUD (3)<br>STONE WITH MUD (4)<br>PLYWOOD (5)<br>CARDBOARD (6)<br>REUSED WOOD (7)<br>*FINISHED WALLS*<br>CEMENT (8)<br>STONE WITH LIME/CEMENT (9)<br>BRICK (10)<br>CEMENT BLOCKS (11)<br>WOOD PLANKS (12)<br>OTHER (SPECIFY) (13): | |
| C23 | Kodi alimo wina aliyense mbanja mwanu amene ali ndi malo ocitilapo nchito ya za ulimi? | YES (1)<br>NO (0)<br>DON'T KNOW (96) | If (0) or (96), skip to C25 |
| C24 | Ndi ma yekala kapena malo ya akulu bwanji amene banja lanu lilanao? | LIMA (1) QUANTITY<br>ACRES (2)<br>HECTARES (3)<br>SQUARE METERS (4)<br>DON'T KNOW (96) | |

SURVEY ID

| C25 | Kodi ndizingati mwa ziwetozi banja lanu lirinazo? | | NUMBER | NONE (00) | DON'T KNOW (96) |
|---|---|---|---|---|---|
| | A | NGOMBE ZA KUMUDZI | | ☐ | ☐ |
| | B | NGOMBE ZA MKAKA | | ☐ | ☐ |
| | C | NGOMBE | | ☐ | ☐ |
| | D | HORSES KAPENA DONKEYS KAPENA MULES | | ☐ | ☐ |
| | E | MBUZI | | ☐ | ☐ |
| | F | MBELELE | | ☐ | ☐ |
| | G | NKHUMBA | | ☐ | ☐ |
| | H | NKHUKU/ KAPENA ZINA ZA MIYENDO IWIRI | | ☐ | ☐ |
| | I | KALULU | | ☐ | ☐ |
| | J | ZIWETO ZINA | | ☐ | ☐ |

| NO. | QUESTION | POTENTIAL RESPONSES | SKIP |
|---|---|---|---|
| C26 | Musonyeze zonse njira zamene mupedzelamo ndalama pano pakhomo mu myezi yokwana khumi ndi ziwili (12) zapita.<br><br>*Select all that apply.* | SALARIED EMPLOYMENT (1)<br>SMALL BUSINESS, SHOP OR KIOSK (2)<br>SMALL HOUSEHOLD INCOME GENERATING ACTIVITY (3)<br>DOWRY (4)<br>SALE OF CROPS/ANIMALS (5)<br>SALE OF ASSETS (6)<br>REMITTANCES (CASH DONATIONS FROM FRIENDS/FAMILY) (7)<br>GOVERNMENT/NGO AID, GRANT OR OTHER FINANCIAL SUPPORT (8)<br>CASUAL DAILY WORK (9)<br>OTHER (SPECIFY) (10): | |
| C27 | Mutafuna kukongola ndalama kucokela ku banki kapena bungwe lina lililonse (kucoselako abwenzi ndi abale), kodi banja lanu linga kwanise kukongola ndalama? | NO (0)<br>PROBABLY NOT (1)<br>PROBABLY YES (2)<br>DEFINITELY YES (3)<br>DON'T KNOW (96) | |
| C28 | Kodi akazi ali napakati ndi ana azaka zochepekera zisanu (5) anagona mu kombe la udzuzu usiku watha? | YES (1)<br>NO (0)<br>DON'T KNOW (96) | |

| | | | |
|---|---|---|---|
| C29 | Mumakwanilisa kulipira zonse zofunika za ana kusukulu, ndalama zolipiliwa ndi zofunika zina? | YES (1)<br>USUALLY (2)<br>SOMETIMES (3)<br>RARELY (4)<br>OTHER (SPECIFY) (5):<br>NO (0) | |
| C30 | Mumwezi wathawu, paliko bena amene anakhala tsiku lathunthu ndi usiku kosadya cakudya ciliconse? | YES (1)<br>NO (0)<br>DON'T KNOW (96) | |
| C31 | Kodi paliko ana aliwonse anagona njala usiku wathawu? | YES (1)<br>NO (0)<br>DON'T KNOW (96) | |
| C32 | Kodi nuumba yanu itha kupirira mphepo kapena mvula yamablogu popanda kuwononga? | YES (1)<br>NO (0)<br>DON'T KNOW (96) | |
| C33 | Ngati kwagwa mvula, mtenje wanyumbayi kumbali yomwe kugona ana siyathonya madzi? | YES (1)<br>NO (0)<br>DON'T KNOW (96) | |

## MODULE D. LAST DELIVERY/MOTHERS' SHELTER

***INTERVIEWER:*** *"Tsopano ndizakufunsani mafunso okhuza ubeleki wanu wa catsopano apa. Talingalilani panthaui ya pafupi ndi kubeleka kumanso kubeleka, potsalira tilankhulane pa za chipanda odi ca azimai apakati. Mwakonzekera? "*

| NO. | QUESTION | POTENTIAL RESPONSES | SKIP |
|---|---|---|---|
| D1 | Munabeleka liti mwana wa last kupela? (DD MONTH YYYY) | ___ ___ ___ ___ ___ ___ ___ ___<br>D D M M Y Y Y Y | |
| D2 | Kwatsopano apa, kodi munamvapo za apanda odi ca azimai apakati? | YES (1)<br>NO (0)<br>DON'T KNOW (96) | If (0) or (96), skip to D18 |
| D3 | Kodi munamvapo kwayani pa aya manyumba?<br><br>Muyankhe zonse zopezekapo | CHIEF (1)<br>HEADMEN (2)<br>HEALTH CARE WORKER (3)<br>SMAG (4)<br>TRADITIONAL BIRTH ATTENDANT (5)<br>FAMILY MEMBER (6)<br>ANOTHER MOTHER (7)<br>OTHER COMMUNITY MEMBER (8)<br>RADIO (9)<br>OTHER (SPECIFY) (10): | |
| D4 | Pakuganizira pa zaubeleki takhala tikuyankhulapo, kodi munakhalapo muzi-panda odi izi mukalibe kubeleka kapena mutabeleka kale? | YES (1)<br>NO (0) | If (1), skip to D5 |
| D4a | If NO, why? | NO MOTHERS SHELTER (1) | Skip to D18 |

| | | | |
|---|---|---|---|
| | *Select all that apply.* | NO PERMISSION FROM HUSBAND OR FAMILY (2)<br>NO MONEY (3)<br>POOR QUALITY (4)<br>NOT CLEAN (5)<br>TOO CROWDED (6)<br>NOT CULTURALLY APPROPRIATE (7)<br>NOT SAFE (8)<br>DELAYS DELIVERY (10)<br>DIDN'T KNOW ABOUT MOTHERS SHELTER (11)<br>OTHER (SPECIFY) (12): | |

**INSTRUCTIONS:** Ask the respondent for what reason(s) did she stay at a mothers' shelter, and then prompt her with the reasons listed below.

| D5 | Masiku yangati mudakhala ku zipanda odi komanso pa zifukwa zotani? | | NUMBER OF NIGHTS | NONE (0) | DON'T KNOW (96) |
|---|---|---|---|---|---|
| | A | ULENDO WA ANC WOYAMBA | ☐☐ | ☐ | ☐ |
| | B | MAULENDO YA ANC ENA | ☐☐ | ☐ | ☐ |
| | C | PAMENE TIKUYEMBEKEZERA MIMBA YOBELEKA | ☐☐ | ☐ | ☐ |
| | D | PAMBUYO POCHOSEDWA MUCHIPATALA KAPENA PAMBUYO POBELEKA | ☐☐ | ☐ | ☐ |
| | E | SIKU LACHITATU (3) PAMBUYO PA ULENDO WA POST NATAL | ☐☐ | ☐ | ☐ |
| | F | SIKU LOKWANA KUYAMBA PA ZISANU NDI ZIWIRI (7) KUFIKA PA KHUMI NDI ZINAYI (14) PAMBUYO PA ULENDO WA POST NATAL | ☐☐ | ☐ | ☐ |
| | G | MILUNGU zisanu ndi chimodzi (6) PAMBUYO WA ULENDO WA POST NATAL | ☐☐ | ☐ | ☐ |
| | H | ZINA (MUTCHULE) | ☐☐ | ☐ | ☐ |

| NO. | QUESTION | POTENTIAL RESPONSES | SKIP |
|---|---|---|---|
| D6 | Nipati pa cipanda odi pamene inu munankala kwanthawi yaitali? | CHOMA DISTRICT<br>CHOMA GENERAL (801001)<br>MANGUNZA (801019)<br>MACHA MISSION (801002)<br>MASUKU MISSION (801021) | |

| | | | |
|---|---|---|---|
| | *Confirm the longest number of nights the respondent stayed at a mothers' shelter.* | MBABALA (801022)<br>MOCHIPAPA (801023)<br>SIMAKUTU (801043)<br>KALOMO DISTRICT<br>CHIFUSA HC (804023)<br>CHILALA HC (804024)<br>DIMBWE HC (804019)<br>HABULILE HC (804032)<br>KALOMO DISTRICT HOSPITAL (804002)<br>KANCHELE HC (804014)<br>MAWAYA HC (804034)<br>MOONDE HP (804042)<br>MUKWELA HC (804020)<br>SIACHITEMA HC (804013)<br>PEMBA DISTRICT<br>JEMBO (801413)<br>MUZOKA (801419)<br>NYIMBA DISTRICT<br>CHIPEMBE RHC (307010)<br>HOFMEYR ZONAL HC (307011)<br>KACHOLOLA RHC (307012)<br>MKOPEKA RHC (307016)<br>NYIMBA DISTRICT HOSPITAL (307001)<br>MANSA DISTRICT<br>FIMPULU (403017)<br>KABUNDA (403018)<br>LUBENDE (403041)<br>MANO (403026)<br>MANSA GENERAL HOSPITAL (403001)<br>MIBENGE (403029)<br>MUSAILA (403030)<br>MUTITI (403031)<br>MUWANGUNI (403032)<br>CHEMBE DISTRICT<br>KUNDAMFUMU (403023)<br>LUKOLA (403037)<br>LUNDAZI DISTRICT<br>CHIKOMENI (405026)<br>KAMSARO (305034)<br>KAPICHILA (305023)<br>LUKWISIZI (305040)<br>LUNDAZI HOSPITAL (305032)<br>LUSUNTHA (305021)<br>MWASE LUNDAZI ZONAL (305011)<br>NKHANGA (305046)<br>NYANGWE (305020)<br>PHIKAMALAZA (305031)<br>ZUMWANDA (305024)<br>OTHER (SPECIFY NAME OF HEALTH FACILITY AND DISTRICT) (47): | |

***INTERVIEWER***: *"Tsopano ine ndikuti ndikufunseni inu zo munapitamo pamene munali kucipanda odi. Patulani nthawi kuganizira nkalidwe yanu panthawi iyo. Mwakonzeka kuyamba? "*

| | | | | |
|---|---|---|---|---|
| D7 | Pamene munali kunkala kucipanda odi amayi ... | **YES (1)** | **NO (0)** | **DON'T KNOW (96)** |

| | | | | | |
|---|---|---|---|---|---|
| | A | PADALI BEDI KAPENA MATIRESI YOGONAPO INU? | ☐ | ☐ | ☐ |
| | B | KODI MUNAGABANA BEDI KAPENA MATIRESI NDI MUNTHU WINA PA NTHAWI ILIYONSE | ☐ | ☐ | ☐ |
| | C | KODI INU MUNAGONAPO MU MOSQUITO NET NTHAWI YA USIKU | ☐ | ☐ | ☐ |
| | D | ANA KUDZIWISANI PA ZA MUKHALIDWE KAPENA MALAMULO A MUCHIPANDA ODI PAMENE MULANGO FIKA PAMALO AWA? | ☐ | ☐ | ☐ |
| | E | KODI MUNALI NDI DANGA LAKUGWIRIKA NCHITO MADZI AUKHONDO | ☐ | ☐ | ☐ |
| | F | KODI MUNALI NDI NYALI LA KUUNIKA PAMBUYO PA KU LOWA DZUWA | ☐ | ☐ | ☐ |
| | G | KODI KUNALI MALO OSAMBILA KAPENA YO WASHILA | ☐ | ☐ | ☐ |
| | H | KODI PANALI MALO ABWINO OYIKHA ZINTHU ZANU NDI CHAKUDYA | ☐ | ☐ | ☐ |
| | I | KODI MUNANKHALAPO NDI MAPUNZILO AZAUMOYO | ☐ | ☐ | ☐ |

| NO. | QUESTION | POTENTIAL RESPONSES | SKIP |
|---|---|---|---|
| D8 | Kodi panali malo opikila panyumba yachipanda odi? | YES (1)<br>NO (0)<br>DON'T KNOW (96) | If (0) or (96), skip to D10 |
| D9 | Kodi malo yophikilayo yanali ndi mutenje kapena ai? | YES (1)<br>NO (0)<br>DON'T KNOW (96) | |
| D10 | Kodi inu munapezako luso latsopano pamene munali kuchipanda odi? | YES (1)<br>NO (0)<br>DON'T KNOW (96) | If (0) or (96), skip to D12 |
| D11 | luso lotani limene munatenga? | | |

SURVEY ID

| | | | |
|---|---|---|---|
| D12 | Kodi anakufunsani kuti mulipile ndalama iliyonse kuti mukhalepo panyumbai? | YES (1)<br>NO (0)<br>DON'T KNOW (96) | If (0) or (96), skip to D14 |
| D13 | Nenani ni ndalama zingati zomwe munalipila zonse pamozi? | | |
| D14 | Kodi anakufunsani kulipila kanthu kena kalikonse paku khalapa nyumbai? | YES (1)<br>NO (0)<br>DON'T KNOW (96) | If (0) or (96), skip to D16 |
| D15 | Munapelekanji?<br><br>*(Select all that apply)* | LABOR (1)<br>LIVESTOCK/POULTRY (2)<br>FOOD OR OTHER AGRICULTURAL RESOURCES (3)<br>OTHER IN-KIND RESOURCES (SPECIFY) (4):<br>OTHER (SPECIFY) (5): | |

| | | | | | | |
|---|---|---|---|---|---|---|
| D16 | ***OFUNSA MAFUNSO:*** *"*Lomba nizakufunsani mafunso pamavuto yoziwika yomwe azima amapeza pa nthawi yokhalamumanyumba yoyembekezela azimai akalibe ucila. Pali zonse izi zati nichule, conde inu niuzeni ngati munapezako/upezamo bvuto, pakakhalidwe kanu munyumba ya azimai zoyembekezela ndiponso munene ngati inali vuto lalikulu kapena ai.*"* | | | | | |
| | | | **MAJOR PROBLEM (2)** | **MINOR PROBLEM (1)** | **NO PROBLEM (0)** | **UNDECIDED (96)** |
| | A | ZONSE MUNYUMBAI NI ZA MTENGO WAPATALI | ☐ | ☐ | ☐ | ☐ |
| | B | KASUNGIDWE KA MALO NDI ZOLAKWIKA | ☐ | ☐ | ☐ | ☐ |
| | C | UKHONDO | ☐ | ☐ | ☐ | ☐ |
| | D | KUPEZEKA KWA ANYANCHITO | ☐ | ☐ | ☐ | ☐ |
| | E | UBWENZI WA ANYANCHITO | ☐ | ☐ | ☐ | ☐ |
| | F | UFULU WOFIKA KUMALO OPHIKIRA | ☐ | ☐ | ☐ | ☐ |
| | G | KUMAPEZEKA ANTHU AMBIRI | ☐ | ☐ | ☐ | ☐ |
| | H | ZA CITETEZO | ☐ | ☐ | ☐ | ☐ |
| | I | KUNVESA ULESI | ☐ | ☐ | ☐ | ☐ |
| | J | MIYAMBO YOYENELA | ☐ | ☐ | ☐ | ☐ |

| NO. | FUNSO | YANKHO YOYENERA | PITANI |
|---|---|---|---|
| D17 | Mkalidwe waa'panda odi unali okondwelesa bwanji? | KWAMBIRI ZOKWANILITSIDWA (1)<br>KAPENA ZOCHEPA ZOKWANILITSIDWA (2)<br>SAKUKHUTITSIDWA (3) | |
| D17a | Kodi muli ndiganizo yoza yembekezela kuchipanda odi pa ubeleki wa msogolo? | YES (1)<br>NO (0)<br>DON'T KNOW (96) | |

| | | | |
|---|---|---|---|
| D17b | Kodi mungalibikitse abale kapena abwenzi kusebenzetsa zipanda odi? | YES (1)<br>NO (0)<br>DON'T KNOW (96) | |

***INTERVIEWER:** "Zikomo pa kuyankha mafunso ya zipanda odi. Tsopano ine ndikufuna kukambirana nanu pa za ubeleka wanu wa posachedwapa kachiwiri. "*

| | | | |
|---|---|---|---|
| D18 | Nindani anakuthandizani pakucila nthawi yathai?<br><br>*Select all that apply.*<br><br>*If respondent says NO ONE ASSSISTED, probe to determine whether any adults were present at the delivery.* | DOCTOR/CLINICAL OFFICER (1)<br>NURSE/MIDWIFE (2)<br>OTHER HEALTH FACILITY STAFF/PERSONNEL (3)<br>TRADITIONAL BIRTH ATTENDANT (4)<br>SMAG (5)<br>RELATIVE/FRIEND/AUNTIE (6)<br>NO ONE ASSISTED (7)<br>OTHER (SPECIFY) (8): | |
| D18a | Munali kufuna kakacilira kuti? | YOUR HOME (1)<br>OTHER HOME (2)<br>HEALTH POST/FACILITY (3)<br>HOSPITAL (4)<br>OTHER (SPECIFY) (5): | |
| D18b | Munali kufuna kukachilira kuti pa ubeleki wa mtsogoro? | YOUR HOME (1)<br>OTHER HOME (2)<br>HEALTH POST/FACILITY (3)<br>HOSPITAL (4)<br>OTHER (SPECIFY) (5): | |
| D19 | Kodi muna belekela kuti mwana wanu wotsilizila? | YOUR HOME (1)<br>OTHER HOME (2)<br>HEALTH POST/FACILITY (3)<br>HOSPITAL (4)<br>OTHER (SPECIFY) (5): | If (1), (2) or (5), skip to D31 |
| **INSTRUCTIONS:** If respondent answers **OTHER (5) to Question D19**, probe to ensure this is not a health post/facility or hospital. | | | |

| **FACILITY-BASED DELIVERY** | | | |
|---|---|---|---|
| **NO.** | **QUESTION** | **POTENTIAL RESPONSES** | **SKIP** |
| D20 | Nicipatala citi coyamba camene munapitako kuti mubeleke nthawi yathai?<br><br>*(**INSTRUCTIONS:** if woman reports hospital, probe to ensure she did not first present at a health facility and was transferred to hospital.)* | CHOMA DISTRICT<br>CHOMA GENERAL (801001)<br>MANGUNZA (801019)<br>MACHA MISSION (801002)<br>MASUKU MISSION (801021)<br>MBABALA (801022)<br>MOCHIPAPA (801023)<br>SIMAKUTU (801043)<br>KALOMO DISTRICT<br>CHIFUSA HC (804023)<br>CHILALA HC (804024)<br>DIMBWE HC (804019)<br>HABULILE HC (804032)<br>KALOMO DISTRICT HOSPITAL (804002)<br>KANCHELE HC (804014) | |

| | | | |
|---|---|---|---|
| | | MAWAYA HC (804034)<br>MOONDE HP (804042)<br>MUKWELA HC (804020)<br>SIACHITEMA HC (804013)<br>PEMBA DISTRICT<br>JEMBO (801413)<br>MUZOKA (801419)<br>NYIMBA DISTRICT<br>CHIPEMBE RHC (307010)<br>HOFMEYR ZONAL HC (307011)<br>KACHOLOLA RHC (307012)<br>MKOPEKA RHC (307016)<br>NYIMBA DISTRICT HOSPITAL (307001)<br>MANSA DISTRICT<br>FIMPULU (403017)<br>KABUNDA (403018)<br>LUBENDE (403041)<br>MANO (403026)<br>MANSA GENERAL HOSPITAL (403001)<br>MIBENGE (403029)<br>MUSAILA (403030)<br>MUTITI (403031)<br>MUWANGUNI (403032)<br>CHEMBE DISTRICT<br>KUNDAMFUMU (403023)<br>LUKOLA (403037)<br>LUNDAZI DISTRICT<br>CHIKOMENI (405026)<br>KAMSARO (305034)<br>KAPICHILA (305023)<br>LUKWISIZI (305040)<br>LUNDAZI HOSPITAL (305032)<br>LUSUNTHA (305021)<br>MWASE LUNDAZI ZONAL (305011)<br>NKHANGA (305046)<br>NYANGWE (305020)<br>PHIKAMALAZA (305031)<br>ZUMWANDA (305024)<br>OTHER (SPECIFY NAME OF HEALTH FACILITY AND DISTRICT) (47): | |
| D21 | Kodi popita kucipatala munapita bwanji? | WALKING (1)<br>BICYCLE (2)<br>CARRIED IN WHEELBARROW (3)<br>ANIMAL-DRAWN CART (4)<br>TAXI (5)<br>CAR (6)<br>MOTORCYCLE (7)<br>AMBULANCE (8)<br>OTHER (SPECIFY) (9): | |
| D22 | Munatenga maola angati kukafika kucipatala?<br><br>*Be sure to specify unit of response.* | HOURS MINUTES | |

| | | | |
|---|---|---|---|
| D23 | Kodi munabelekela kucipatala komwe mumabelekela ana anu kwanthawi zonse? | YES (1)<br>NO (0)<br>DON'T KNOW (96) | If (1) or (96) skip to D27 |
| D24 | Kodi ndi dzina lotani la chipatala mudabereka mwana wanu? | CHOMA DISTRICT<br>CHOMA GENERAL (801001)<br>MANGUNZA (801019)<br>MACHA MISSION (801002)<br>MASUKU MISSION (801021)<br>MBABALA (801022)<br>MOCHIPAPA (801023)<br>SIMAKUTU (801043)<br>KALOMO DISTRICT<br>CHIFUSA HC (804023)<br>CHILALA HC (804024)<br>DIMBWE HC (804019)<br>HABULILE HC (804032)<br>KALOMO DISTRICT HOSPITAL (804002)<br>KANCHELE HC (804014)<br>MAWAYA HC (804034)<br>MOONDE HP (804042)<br>MUKWELA HC (804020)<br>SIACHITEMA HC (804013)<br>PEMBA DISTRICT<br>JEMBO (801413)<br>MUZOKA (801419)<br>NYIMBA DISTRICT<br>CHIPEMBE RHC (307010)<br>HOFMEYR ZONAL HC (307011)<br>KACHOLOLA RHC (307012)<br>MKOPEKA RHC (307016)<br>NYIMBA DISTRICT HOSPITAL (307001)<br>MANSA DISTRICT<br>FIMPULU (403017)<br>KABUNDA (403018)<br>LUBENDE (403041)<br>MANO (403026)<br>MANSA GENERAL HOSPITAL (403001)<br>MIBENGE (403029)<br>MUSAILA (403030)<br>MUTITI (403031)<br>MUWANGUNI (403032)<br>CHEMBE DISTRICT<br>KUNDAMFUMU (403023)<br>LUKOLA (403037)<br>LUNDAZI DISTRICT<br>CHIKOMENI (405026)<br>KAMSARO (305034)<br>KAPICHILA (305023)<br>LUKWISIZI (305040)<br>LUNDAZI HOSPITAL (305032)<br>LUSUNTHA (305021)<br>MWASE LUNDAZI ZONAL (305011)<br>NKHANGA (305046)<br>NYANGWE (305020)<br>PHIKAMALAZA (305031)<br>ZUMWANDA (305024)<br>OTHER (SPECIFY NAME OF HEALTH FACILITY AND DISTRICT) (47): | |

| | | | |
|---|---|---|---|
| D25 | Kodi anakutumani kapena kukupelekani kucipatalaci anchito zacipatala? | YES (1)<br>NO (0)<br>DON'T KNOW (96) | If (0) or (96), skip to D27 |
| D26 | Panapita nthawi yayi tali bwanji pamene inu munapelekedwa kuchipatala? | LESS THAN 1 HOUR (1)<br>1 TO 2 HOURS (2)<br>MORE THAN 2 HOURS (3) | |
| D27 | Kodi anapanga ganizo loka belekela kucipatalaci ndani? | YOURSELF (1)<br>HUSBAND/PARTNER (2)<br>MOTHER/MOTHER-IN-LAW (3)<br>AUNTIE (4)<br>OTHER FAMILY MEMBER (5)<br>FRIEND (6)<br>OTHER (SPECIFY) (7): | |
| D28 | Kodi munankhala kucipatala kwa maola osacepekela makhumi yabili ndi anai (24) pambuyo pobeleka musanatulutsidwe? | YES (1)<br>NO (0)<br>DON'T KNOW (96) | |

| D29 | ***INTERVIEWER:*** *"*Apa ndizacula zinthu zothandizila anthu zomwe acipatala amacitandi lingo lofuna kudziwa ngati munalandilako zina mwa izi pomwe munapita kukabeleka nthawi yathai.*"* | | | |
|---|---|---|---|---|
| | | **RECEIVED (1)** | **DID NOT RECEIVE (0)** | **DON'T KNOW (96)** |
| A | Kodi mwana wanu wotsiriza anabadwa munjira ya opareshoni? Ici citanthauza kutsegula kwa pamimba ndi kucotsamo mwana ndi kutsekanso? | ☐ | ☐ | ☐ |
| B | Kodi pomwe munabeleka anakuikanikoni magazi? | ☐ | ☐ | ☐ |
| C | Munkhwala wama Antibiotics kapenanso intravenous (IV) drip-kuikiwa madzi mthupi | ☐ | ☐ | ☐ |
| D | Uphunzitsiwa pa kuyamwisa mkaka mwana | ☐ | ☐ | ☐ |
| E | Kukonzekera mankhwala ya cilezi kapena uphungu | ☐ | ☐ | ☐ |
| F | Uphungu osamala mwana ngati kangaroo (khungu kwa khungu) amasamalila mwana wake | ☐ | ☐ | ☐ |

| D30 | ***INTERVIEWER:*** *"*Apa lomba nizafunsa paza mavuto yomwe azimai amapitamo kuja kucipatala pa nthawi yobeleka. Ndiza chula vuto imodzi-imodzi, ndiye munene ngati inalipodi vuto yoteleyi ndiponso munene ukuluwake wavutoli kwa inu kulingana ndi zamene munapitamo.*"* | | | | |
|---|---|---|---|---|---|
| | | **VUTO LALIKULU (2)** | **VUTO LALING'ONO (1)** | **KULIBE VUTO (0)** | **SADZIWA (96)** |
| A | MTENGO NDI UBWINO WA MOMWE ANAKUSAMALILANI ACIPATALA POBELEKA | ☐ | ☐ | ☐ | ☐ |
| B | ULEMU OMWE ANAKUPASANI ANCHITO ZACIPATALA | ☐ | ☐ | ☐ | ☐ |

| | | | | | | |
|---|---|---|---|---|---|---|
| | C | KUBELEKELA MUCIPINDA COBISIKA | ☐ | ☐ | ☐ | ☐ |
| | D | UKHONDO KAPENA ODONGO WA PACIPATALA | ☐ | ☐ | ☐ | ☐ |
| After completing the facility-based delivery section, continue to MODULE E. | | | | | | |

| HOME DELIVERIES | | | |
|---|---|---|---|
| **No.** | **Question** | **Potential responses** | **Skip** |
| D31 | Nindani analamulila kuti inu mubelekele kunyumba? | YOURSELF (1)<br>HUSBAND/PARTNER (2)<br>MOTHER/MOTHER-IN-LAW (3)<br>AUNTIE (4)<br>OTHER FAMILY MEMBER (5)<br>FRIEND (6)<br>OTHER (SPECIFY) (7): | |
| D32 | Nicifukwa chotani chamene inu simuna belekele kucipatala?<br><br>*Select all that apply.* | COST TOO MUCH (1)<br>FACILITY NOT OPEN (2)<br>TOO FAR/NO TRANSPORTATION (3)<br>POOR QUALITY SERVICE/DON'T TRUST (4)<br>NO FEMALE HEALTH PROVIDER (5)<br>HUSBAND/FAMILY DIDN'T ALLOW (6)<br>SHORT LABOR (7)<br>BABY CLOTHES (8)<br>CDK (9)<br>NO MOTHERS SHELTER (10)<br>NOT NECESSARY (11)<br>NOT CUSTOMARY (12)<br>OTHER (SPECIFY) (13): | |
| D33 | Kodi inu munapita kuti chipatala kukayesedwa thanzi lanu ndi thanzi la mwana wanu pasanathe maola 24 atabeleka? | YES (1)<br>NO (0)<br>DON'T KNOW (96) | If 0 or 96, skip to Module E |

## MODULE E: SPENDING AND SAVINGS

***INTERVIEWER:*** *"Tsopano ine ndati ndikufunseni inu za ndalama za mimba yanu yomaliza ndi za kubeleka. Taganizirani za ndalama kugwirizana ndi mimba yanu ndi za kubeleka ndi mmene munakonzekela ndalama zimenezo. "*

| E1 | ***OFUNSA MAFUNSO:*** *"Tsopano tikufuna kulankhula nkani za ndalama zina gwilisidwa nchito pakubeleka wotsiriza. Ganizilani zinthu zimene munagula pokonzekera nkhani yanu, ulendo wanu kuchipatala kapena kunyumba kumene inu anapulumutsidwa, ndipo pa nthawi yakubeleka anu kucipatala kapena kunyumba kumene inu anabelekela. Mwakonzeka kuyamba? "* | | | | |
|---|---|---|---|---|---|
| | Kodi ni ndalama zingati zimene munasebenzesa pa: | | **AMOUNT (KWACHA)** | **NONE (0)** | **DON'T KNOW (96)** |
| | ***POKONZEKELA:*** | | | | |
| | A | ZOFUNIKILA (kuphatikizapo zida zofunika kuti mubeleke moyenera, magolovesi, | | | |

| | | | | | |
|---|---|---|---|---|---|
| | | syringes, mapepala apulasitiki, tocingiliza matenda, etc.) | | | |
| | B | ZOVALA ZA MWANA KAPENA MA BULANGETI | | | |
| | **Pa ulendo wanu:** | | | | |
| | C | MAYENDEDWE (ngati ninyumba yanu, lembani 0) | | | |
| | D | CIPANDA ODI KAPENA MALO ENA POYEMBEKEZA KUBELEKA | | | |
| | Pa nthawi yobeleka: | | | | |
| | E | MALIPILO YA OGWIRA NCHITO KAPENA YACIPATALA | | | |
| | F | MALIPIRO YACIBWENZI | | | |
| | G | KANGACEPE | | | |
| | H | MALIPILO ENA OSATI NDALAMA (pimani tandiso mu kwacha) | | | |
| | I | MANKHWALA | | | |
| | J | OYESA MATENDA | | | |
| | K | ZOLIPIRA ZINA | | | |

| NO. | QUESTION | POTENTIAL RESPONSES | SKIP |
|---|---|---|---|
| E2 | Kodi munasungapo ndalama zogwiritsa nchito nthawi yokacila? | YES (1)<br>NO (0)<br>DON'T KNOW (96) | If (0) or (96), skip to E8 |
| E3 | Kodu muganizira kuti munasunga ndalama zokwanila pa ubeleki wanu othela (pacikonzekelo ca ulendo wakupita kukacila)? | YES (1)<br>NO (0)<br>DON'T KNOW (96) | |
| E4 | Kodi muna sungila kuti ndalamazi? | AT YOUR HOME (1)<br>AT A FRIEND OR FAMILY MEMBER'S HOME (2)<br>IN A BANK ACCOUNT (3)<br>OTHER (SPECIFY) (4): | |
| E5 | Kodi kuliko wina aliyense (mwa cinsanzo amuna anu) amene anali kuziwa malo munasungila ndalama? | YES (1)<br>NO (0)<br>DON'T KNOW (96) | |

SURVEY ID | | | | | | | | | |

| | | | |
|---|---|---|---|
| E6 | Kodi mimba yanu inali ya ikulu bwanji pamane munayamba ku sunga ndalama zobelekela? | WEEKS<br>MONTHS | |
| E7 | Kodi ndani wina anatandizila ndalama zobelekela (pokonzekela, pa ulendo, pa nthawi yobeleka)?<br><br>*(Select all that apply)* | HUSBAND/PARTNER (1)<br>YOUR CHILDREN (2)<br>PARENT/GRANDPARENT (3)<br>OTHER FAMILY MEMBER (4)<br>FRIEND (5)<br>AUNTIE (6)<br>NO ONE (7)<br>OTHER (SPECIFY) (8): | |
| E8 | Mumaganizo anu, n'chifukwa chiyani tiyenera kusunga ndalama zobelekela? | NOT IMPORTANT (1)<br>SLIGHTLY IMPORTANT (2)<br>MODERATELY IMPORTANT (3)<br>IMPORTANT (4)<br>VERY IMPORTANT (5) | |
| E9 | Kodi munasungisapo ndalama ku banki? | YES (1)<br>NO (0)<br>DON'T KNOW (96) | |
| E10 | Kodi munatumizapo kapena kutumiziwa ndalama pa lamia ("ndalama zapa lamia")? | YES (1)<br>NO (0)<br>DON'T KNOW (96) | In (0) or (96), skip to module F |
| E11 | Kodi muna tumila ndani “mobile money”? | HUSBAND/PARTNER (1)<br>YOUR CHILDREN (2)<br>PARENT/GRANDPARENT (3)<br>OTHER FAMILY MEMBER (4)<br>FRIEND (5)<br>AUNTIE (6)<br>OTHER (SPECIFY) (7): | |

## MODULE F. POST-NATAL CARE

***INTERVIEWER:*** *"Tsopano ine ndikufuna kuti ndikufunseni inu mafunso angapo za chisamaliro za umoyo wanu ndi mwana wanu analandira pambuyo paubeleki wanu wotsiriza."*

| NO. | QUESTION | POTENTIAL RESPONSES | SKIP |
|---|---|---|---|
| F1 | Kodi inu munapita kucipatala CILI CONSE ca postnatal pambuyo pa maola khumi ndi zinayi pambuyo pakubeleka komaliza? | YES (1)<br>NO (0)<br>DON'T KNOW (96) | If (0) or (96) skip to F6 |
| F2 | Kodi inu munapita kucipatala ca postnatal pambuyo pama siku | YES (1)<br>NO (0)<br>DON'T KNOW (96) | |

| | | | |
|---|---|---|---|
| | yatatu yapambuyo pakubeleka komaliza? | | |
| F3 | Kodi inu munapita kucipatala cili conse ca postnatal pakati pama siku asanu ndi awiri (7) ndi khumi ndizinayi (14) pambuyo pakubeleka komaliza? | YES (1)<br>NO (0)<br>DON'T KNOW (96) | |
| F4 | Kodi inu munapita ku kucipatala CILI CONSE cha postnatal masabata asanu ndi imodzi (6) mutabeleka komaliza? | YES (1)<br>NO (0)<br>DON'T KNOW (96) | |

| F5 | ***INTERVIEWER:*** *"*Lomba nizakufunsani mavuto yoziwika yomwe azimai apitamo kuja kucipatala pokalandila thandizo lopelekewa ku mzimai ndi pambuyo pobeleka. Pali zomwe ndiza culazi inu munene ngati munazipeza kukhala zokuvutani pomwe munapita kukaonewa pambuyo pobeleka mwana nthawi yathayi ndiponso munene ngati inali vuto lalikulu kapena ayi.*"* | | | | | |
|---|---|---|---|---|---|---|
| | | | **MAJOR PROBLEM (2)** | **MINOR PROBLEM (1)** | **NO PROBLEM (0)** | **UNDECIDED (96)** |
| | A | NTHAWI YOMWE MUNA YEMBEKEZELA KUTI MUONANE NDI ACIPATALA | ☐ | ☐ | ☐ | ☐ |
| | B | MPATA WOKAMBA PA ZAMAVUTO KAPENA ZODESA NKHAWA ZOKHUZA PAKATI | ☐ | ☐ | ☐ | ☐ |
| | C | YANKHO YOMWE MUNAPATSIDWA KAPENA ZOMWE ANAKUCITILANI PAZA VUTOLIJA | ☐ | ☐ | ☐ | ☐ |
| | D | KUPIMIWA MU MALO OBISIKA KOMWE ENA SIANGAONEKO | ☐ | ☐ | ☐ | ☐ |
| | E | KUKAMBITSANA MWA CISINSI KULUBE ENA ALIWONSE OMVELAKO ZOKAMBIDWAZO | ☐ | ☐ | ☐ | ☐ |
| | F | UKHONDO WAKE WAPACIPATALAPO | ☐ | ☐ | ☐ | ☐ |
| | G | ZOMWE ANCHITO ZACIPATALA ANAKUCITILANI | ☐ | ☐ | ☐ | ☐ |
| | H | MTENGO WA THANDIZO KAPENA MKHWALA OMWE MUNAPASIDWA | ☐ | ☐ | ☐ | ☐ |

| | | | |
|---|---|---|---|
| F6 | Kodi pali zomwe mugwilitsa ncito, kapena zomwe muyesa zocedwetsa kuima kapena inu kusatenga pathupi? | YES, MODERN METHOD (1)<br>YES, TRADITIONAL METHOD (2)<br>NO (0)<br>N/A, CURRENTLY PREGNANT (3)<br>DON'T KNOW (96) | |
| F7 | **INSTRUCTIONS:** Look back to question **B27** – Kodi mwana wobeleka komaliza ali moyo?<br><br>*Pempani yankho ku oyankha.* | YES (1)<br>NO (0)<br>DON'T KNOW (96) | If (0) or (96) skip to F15 |

SURVEY ID | | | | | | | | | |

| | | | |
|---|---|---|---|
| F8 | Kodi mukali kumuyamwisa mabele mwana kuchokela pamene anabadwa? | YES (1)<br>NO (0)<br>DON'T KNOW (96) | If (0) or (96) skip to F10 |
| F9 | Kodi akudya zakudaya zina mwana mosakaniza ndi mkaka wamawele ndi mankhwala? | YES (1)<br>NO (0)<br>DON'T KNOW (96) | |
| F10 | Mu masabata yabili yomaliza, kodi munafuna kusamalira za umoyo za mwana wanu cifukwa ca ciliconse? | YES (1)<br>NO (0)<br>DON'T KNOW (96) | If (0) or (96), skip to F12 |
| F11 | Kodi inu poyamba muna peleka kuti mwana wanu kufufuza zau moyo? | HEALTH CARE CENTER (1)<br>HOSPITAL (2)<br>PHARMACY (3)<br>TRADITIONAL HEALER (4)<br>OTHER (SPECIFY) (5): | |
| F12 | Kodi mwana wanu analandilapo katemela uliwonse? | YES (1)<br>NO (0)<br>DON'T KNOW (96) | If (0) or (96), skip to F15 |

| | | | | | |
|---|---|---|---|---|---|
| F13 | **INSTRUCTIONS:** Based on **D1**, calculate child's age.<br><br>*Specify unit of response.* | | | | |
| | **INSTRUCTIONS:** Ask to see the child's vaccination card. If available, use card to confirm the vaccines received and mark below. If card is unavailable, ask mother which vaccines the child has received. **BASED ON CALCULATED AGE FROM F13**, ask only about **AGE APPROPRIATE** vaccines. | | | | |
| F14 | Confirm you have the child's vaccine card in-hand. | YES (1)<br>NO (0) | | | |
| | | **CONFIRMED BY VACCINE CARD** | | **CONFIRMED BY MOTHER** | |
| | | RECEIVED | NOT RECEIVED | RECEIVED | NOT RECEIVED |
| | **Kodi mwana wanu analandila katemera wotsatira pakubadwa?** | | | | |
| | A BCG | ☐ | ☐ | ☐ | ☐ |
| | B Polio (OPV-0) | ☐ | ☐ | ☐ | ☐ |
| | **Kodi mwana wanu analandila katemera wotsatira wapa sabata asanu ndi imodzi?** | | | | |
| | C Polio (OPV-1) | ☐ | ☐ | ☐ | ☐ |
| | D DTP-HepB-Hib-1 | ☐ | ☐ | ☐ | ☐ |
| | E Pneumococcal (PCV) | ☐ | ☐ | ☐ | ☐ |
| | F Rotavirus | ☐ | ☐ | ☐ | ☐ |
| | **Kodi mwana wanu analandila katemera wotsatira wapa sabata kumi?** | | | | |
| | G Polio (OPV-2) | ☐ | ☐ | ☐ | ☐ |
| | H DTP-HepB-Hib-2 | ☐ | ☐ | ☐ | ☐ |
| | I Pneumococcal (PCV) | ☐ | ☐ | ☐ | ☐ |
| | J Rotavirus | ☐ | ☐ | ☐ | ☐ |
| | | | | | |

SURVEY ID

| | Kodi mwana wanu analandila katemera wotsatira wapa sabata kumi ndi anayi? | | | | |
|---|---|---|---|---|---|
| | K Polio (OPV-3) | ☐ | ☐ | ☐ | ☐ |
| | L DTP-HepB-Hib-3 | ☐ | ☐ | ☐ | ☐ |
| | M Pneumococcal (PCV) | ☐ | ☐ | ☐ | ☐ |

| ***Interviewer:*** *"*Pamafunso yotsatilawa, conde muyankhe ngati mukumva bwino kuyankha. Mungasankhe kuyankha kapenanso ai." | | | |
|---|---|---|---|
| F15 | Kodi anakupimani paza tilombo twa HIV pamimba yathai? | YES (1)<br>NO (0)<br>PREFER NOT TO ANSWER (2)<br>DON'T KNOW (96) | |
| F16 | Munene ngati muli ndi tilombo twa HIV kapena ayi? | INFECTED (1)<br>NOT-INFECTED (2)<br>PREFER NOT TO ANSWER (3)<br>DON'T KNOW (96) | If (2), (3), or (96), skip to F23 |
| F17 | Kodi munamwa mankhwala ya ARVs pamimba yatha? | YES (1)<br>NO (0)<br>DON'T KNOW (96) | |
| F18 | **INSTRUCTIONS:** Refer back to question **B27-28**. Did the respondent's baby survive beyond the day of birth?<br><br>*Confirm with respondent.* | YES (1)<br>NO (0)<br>DON'T KNOW (96) | If (0) or (96), skip to Module G |
| F19 | Kodi mwana wanu anamwa mankwala yama ARVs osachepera masabata 6 pambuyo yobadwa? | YES (1)<br>YES, BUT BABY DIED BEFORE 6 WEEKS OF AGE (2)<br>NO (0)<br>DON'T KNOW (96) | |
| F20 | Kodi mwana wanu anapimiwapo za matenda ya HIV? | YES (1)<br>NO (0)<br>DON'T KNOW (96) | If (0) or (96), skip to F23 |
| F21 | Anakwanisa ma sabata yangati pamene mwana anapimiwa pa tulombo twa HIV?<br><br>*Round to nearest full number.* | | |
| F22 | Kodi atapimiwa utu tulombo tuna pezeka kapena ayi? | INFECTED (1)<br>NOT INFECTED (2)<br>PREFER NOT TO ANSWER (3)<br>DON'T KNOW (96) | |

| F23 | Mumasiku atatu apitawa, kodi inu kapena wina aliyense munyumba mwanumu munamuchitilako zochulidwazi mwana wanu?<br><br>*Select all that apply* | | | |
|---|---|---|---|---|
| | | **YES (1)** | **NO (0)** | **DON'T KNOW (96)** |
| | A KUMUWELENGELA NTHANO MU MABUKU KAPENA KUMUWONETSA ZIKOPE/PIKICA | ☐ | ☐ | ☐ |

| | | | | | |
|---|---|---|---|---|---|
| | B | KUMUUZA TU NTHANO | ☐ | ☐ | ☐ |
| | C | KUYIMBA NYIMBO KAPENA TUNYIMBO TOYIMBA MWAPANSI KUPANGA MWANA KUGONA | ☐ | ☐ | ☐ |
| | D | KUMUPELEKA MWANA PANJA PANYUMBA, KOMBONI, YADI, KAPENA MMALO OCINGILIZIWA | ☐ | ☐ | ☐ |
| | E | MAINA, KUPENDA, KAPENA KUDROWINGA VINTHU NDI MWANA | ☐ | ☐ | ☐ |

| F24 | ***INTERVIEWER:*** *"For the following questions, please respond only if you feel comfortable doing so. Your response is optional. I am going to read you a list of problems. Please tell me how often each of these problems has happened to you in the PAST TWO WEEKS: never, once in a while, more than half the time, or almost always."* | | | | | |
|---|---|---|---|---|---|---|
| | | | **NEVER (0)** | **ONCE IN A WHILE (1)** | **MORE THAN HALF THE TIME (2)** | **ALMOST ALWAYS (3)** |
| | A | MUMILUNGU IWIRI YAPITAYI, NDINALI OKALIPA KAPENA OSAKONDWELA | ☐ | ☐ | ☐ | ☐ |
| | B | MUMILUNGUIWIRI YAPITAYI, SINDINAKHALE NDICHOFUNA MUZOCHITIKA, KUGWIRA NCHITO, NGAKHALE KU ANTHU | ☐ | ☐ | ☐ | ☐ |
| | C | MUMILUNGU IWIRI YAPITAYI, NDINALIRA | ☐ | ☐ | ☐ | ☐ |
| | D | MUMILUNGU IWIRI YAPITAYI, NDIMALI MAUWEKHA KOMANSO OTAYIKA | ☐ | ☐ | ☐ | ☐ |

| F25 | ***INTERVIEWER:*** *"Now I am going to read you a list of things that you may have experienced. Please tell me how often each of these events have happened to you in the past two weeks: never, once in a while, a few times, or many times. Again, please respond only if you feel comfortable doing so. Your response is optional."* | | | | | | |
|---|---|---|---|---|---|---|---|
| | | | **NEVER (0)** | **ONCE (1)** | **A FEW TIMES (2)** | **MANY TIMES (3)** | **N/A (4)** |
| | A | MUMILUNGU IWIRI YAPITAYI, NDIKANGATI KAMENE AMUNA KAPENA OKONDEDWA ANU ANAKUKANKHANI KAPENA KUKUPANDANI KUTSAYA | ☐ | ☐ | ☐ | ☐ | ☐ |
| | B | MUMILUNGU IWIRI YAPITAYI, NDIKANGATI KAMENE AMUNA ANU KAPENA OKONDEDWA ADAKUKOKOTANI, KUKUPANDANI, KUKUGWIRANI PAKHOSI KAPENA KUKUTENTHANI?? | ☐ | ☐ | ☐ | ☐ | ☐ |

SURVEY ID

## MODULE G. LAST PREGNANCY

***INTERVIEWER:*** *"Tsopano ine ndati ndikufunseni inu mafunso ena amene ali enieni pa mimba yanu kufika kwa kubeleka posachedwa. Ganizilani zimene zinachitika pamene inu munadziwa kuti mulina mimba ndi zokhudza chithandizo chaku antenatal. Mwakonzeka kuyamba? "*

| NO. | QUESTION | POTENTIAL RESPONSES | | | SKIP |
|---|---|---|---|---|---|
| **Interviewer**: *Ask to see if antenatal care card is available for the woman's last pregnancy that led to a delivery and confirm information provided by respondent.* | | | | | |
| G1 | Did the woman provide you with her antenatal card? | YES (1)<br>NO (0)<br>DON'T KNOW (96) | | | |
| G2 | Kodi nikangati komwe inu munalandila thandizo la antenatal pamene munali ndi pakati? | NONE (0)<br>ONE TIME (1)<br>TWO TIMES (2)<br>THREE TIMES (3)<br>FOUR TIMES (4)<br>MORE THAN FOUR TIMES (5) | | | If (0) skip to End of Survey |
| G3 | Pamene munali kukatemela, munakambisanako pa izi: | **YES (1)** | **NO (0)** | **DON'T KNOW (96)** | |
| | A Mungacilile kuti ikakwana nthawi? | ☐ | ☐ | ☐ | |
| | B Munga citenji ngati mwa peza vuto ndi mimba? | ☐ | ☐ | ☐ | |
| | C Kusunga ndalama zolipira mimba ikauka ndi paku beleka? | ☐ | ☐ | ☐ | |
| | D Kodi muganizila kubeleka siku liti? | ☐ | ☐ | ☐ | |
| G4 | Kodi mukumbukira zimene munauzidwa za siku yobeleka? | YES (1)<br>NO (0)<br>DON'T KNOW (96) | | | If (0), skip to G6 |
| G5 | Munali kuyembekezela kubeleka liti? (DD MONTH YYYY)<br><br>*If EDD is on ANC card, copy it from card. If no card and date not know, enter 15th* | ____ ____ ____ ____ ____ ____ ____ ____<br>D D M M Y Y Y Y | | | |
| G6 | Paulendo wanu **WOYAMBA** waku ANC, munali ndi pakati pa ma sabata kapena myezi ingati?<br><br>*Chonde, sankani mopendela yankho.* | WEEKS<br><br>MONTHS | | | |

SURVEY ID | | | | | | | | | |

**INTERVIEWER:** *"Ife tafika ku mapeto a kafukufukuyu. Tikukuthokozani chifukwa chopatula nthawi kuyankha mafunso awa. Kodi muli ndi ndemanga zoonjezera mungakonde kuwonjezera? "*

| | | | |
|---|---|---|---|
| G7 | Would you be willing to have someone come back and follow up on a few questions from the survey in the next couple of days? | YES (1)<br>NO (0) | |

**COMMENTS:**

# END OF SURVEY

## INSTRUMENT REVIEW

| | |
|---|---|
| Enumerator Initials: | |
| Date (DD/MM/YYYY) | |
| Supervisor Initials: | |
| Date (DD/MM/YYYY) | |

| | |
|---|---|
| Data Entry Initials: | |
| Date (DD/MM/YYYY) | |
| Supervisor Initials: | |
| Date (DD/MM/YYYY) | |
